# Programmed guest confinement *via* hierarchical cage to cage transformations†

**DOI:** 10.1039/d3sc01368e

**Published:** 2023-07-04

**Authors:** Federico Begato, Giulia Licini, Cristiano Zonta

**Affiliations:** a Department of Chemical Sciences, University of Padova *via* Marzolo 1 35131 Padova Italy cristiano.zonta@unipd.it

## Abstract

Taking inspiration from Nature, where (bio)molecular geometry variations are exploited to tune a large variety of functions, supramolecular chemistry has continuously developed novel systems in which, as a consequence of a specific stimulus, structural changes occur. Among the different architectures, supramolecular cages have been continuously investigated for their capability to act as functional hosts where guests can be released in a controlled fashion. In this paper, a novel methodology based on the use of phenanthrenequinone is applied to selectively change the binding properties of a tris(2-pyridylmethyl)amine TPMA-based cage. In particular, subcomponent substitution has been used to change structural cage features thus controlling the inclusion ratio of competing guests differing in size or chirality.

## Abstract 2

A novel methodology based on the use of phenanthrenequinone is applied to selectively change the subcomponent of TPMA-based cages, thus controlling inclusion ratio of competing guests differing in size or chirality.

## Graphical abstract

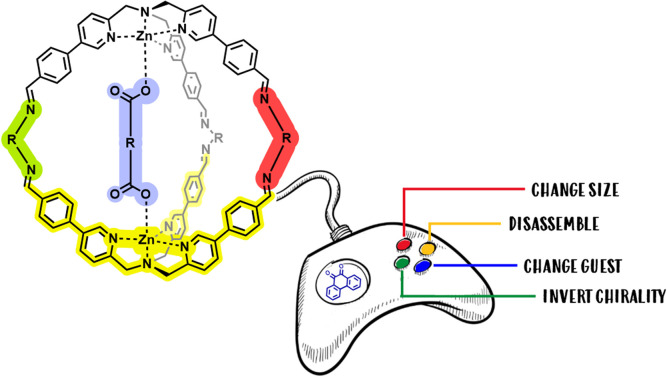

## Introduction

Uptake and release of guests using external stimuli is one of the emerging areas in the field of functional supramolecules.^1–3^ Within this context, supramolecular cages have been continuously challenged for their intrinsic capability to act as functional hosts where guests can be released in a controllable fashion.^4–9^ Due to the dynamic nature of their bonds, assembled cages can transform between geometrically distinct structures formed from the same set of components, giving opportunity to alter selectively their molecular structure as well as their functions.^10–13^ The possibility to regulate guest uptake and release can open in principle novel properties in storage,^14,15^ sensing,^16–18^ and catalysis.^19,20^ Common strategies for release of a guest from cages are based on: (i) the presence of a stronger binder in solution which substitutes the guest,^21^ (ii) disassembly of the host,^22,23^ modification of the (iii) guest (*viz.* protonation/deprotonation, oxidation/reduction, light)^24–28^ or the (iv) host by external factors (*viz.* ligand or subcomponent exchange).^29–31^ The final goal is to mimic the biological complexity where chemical signals regulate activity *via* well-defined transformation of biomolecules.^32–34^

In the recent years we have been interested in the phenomena related to confinement in supramolecular cages.^35^ In particular, within the task of guest release, we reported a delivery strategy that took advantage of the guest size to trigger its release by hydrolysis of a tris(2-pyridylmethyl)amine TPMA-based supramolecular cage.^36^ In this case, the architecture was built up using imine based Dynamic Covalent Chemistry (DCC) and hydrolyzed using water. However, hydrolysis performed using an excess of water does not allow tuning the extent of guest release or coming back to the initial structure.

In the quest to gain additional control of guest release/uptake, the addition of a quinone was planned to offer the possibility to sequestrate diamine cage subcomponents allowing for further transformations. In this article is reported a novel approach based on the capability of 9,10-phenanthrenequinone (4) to react with diamine subcomponents which allows their selective removal or replacement leading to cage disassembly or cage-to-cage conversions. In particular, the developed methodology enabled the selective switch of the cage binding properties towards competing guests present in solution. This strategy was successfully optimized to achieve: (i) reversible assembly and disassembly of the cage with release and uptake of the guest, (ii) hierarchical cage size transformation with differential release and uptake of competing guests in solution differing only in molecular size and (iii) cage chirality inversion which allows alternative enclosure in the cage of two enantiomeric diacids competing in solution.

## Results and discussion

The molecular system under study is based on TPMA supramolecular cages we studied in recent years.^36^ These architectures assemble in solution taking advantage of the capability of aldehydic subcomponent 1 to bind dicarboxylates and pre-organize the system for the assembly.^37^ The system is highly stable and, as reported recently, it can also form in the presence of natural matrixes.^38,39^ The supramolecular architecture is built up using diamines which can be varied to finely tailor recognition properties of the final architecture.^37^

### Cage assembly/disassembly/assembly (case I)

**Fig. 1:**
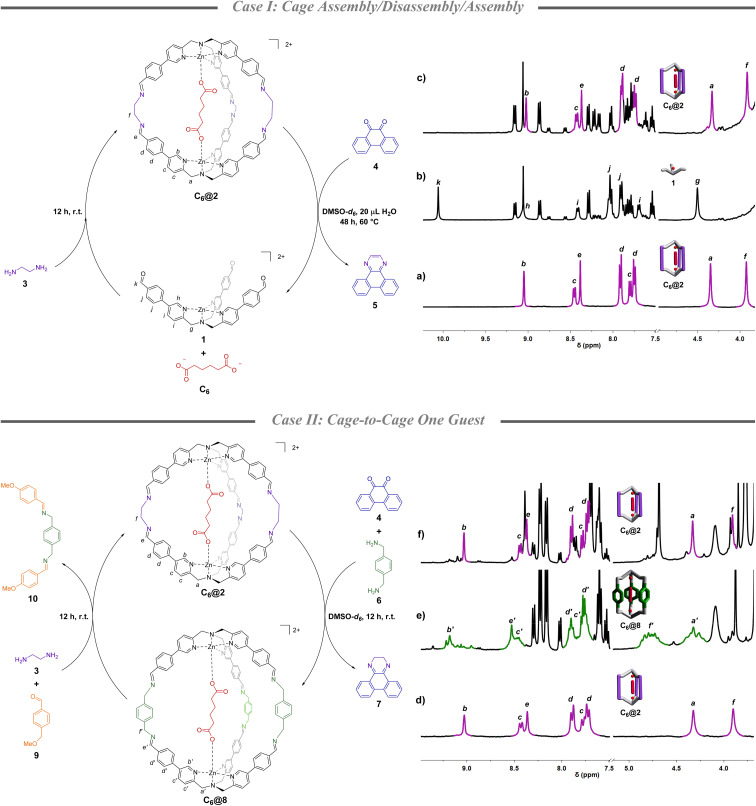
Case I: partial ^1^H-NMR (DMSO-*d*_6_, 400 MHz) of (a) C_6_@2, (b) complex 1 obtained from the cage disassembly using quinone 4, and (c) C_6_@2 re-formed after the addition of ethylenediamine 3 to the mixture. 1,3,5-Trimethoxy benzene is used as the internal standard. Case II: partial ^1^H-NMR (DMSO-*d*_6_, 300 MHz) of (d) C_6_@2, (e) C_6_@8 obtained after the addition of quinone 4 and *p*-xylylenediamine 6 to C_6_@2, and (f) C_6_@2 obtained after the addition of ethylenediamine 3 and *p*-anisaldehyde 9 to C_6_@8. *p*-Xylene is used as the internal standard.

Beginning with the knowledge that quinone 4 is known to form a stable adduct with ethylenediamine (3),^40^ its disassembly capability was initially tested in the presence of a formed cage containing adipate C_6_. More in detail, starting from cage C_6_@2 (Fig. 1a), which was formed in DMSO-*d*_6_ mixing complex 1 (1 eq.), ethylenediamine 3 (2.5 eq.), and C_6_ (0.5 eq.), heating the solution at 60 °C after the addition of a stoichiometric amount of quinone 4 and 20 μL of water resulted in the complete disassembly of the initial architecture after 2 days (Fig. 1b).^41^ This is evidenced by the disappearance of the imine peak at 8.4 ppm and the concomitant formation of the aldehyde peak of 1 at 10 ppm. Ethylenediamine 3 was then added leading to the re-assembly of C_6_@2 in almost quantitative yield (90% in respect to the initial cage, Fig. 1c). The whole process generates a cycle in which the dicarboxylate guest is caught and released from solution. While initially the C_6_ guest is bound to the cage (4 : 1 ratio of caged and in solution), the subsequent disassembly resulted in complete release of the guest. Uptake of the C_6_ guest is achieved in the final re-assembly (2.5 : 1 ratio of caged and in solution) (Section S3†).

The lower inclusion value in the final step is influenced by the dilution of the system which occurs during the additions and by the incomplete reformation of the initial cage. ^1^H NMR spectra also reveal that quinone 4 evolved into the aromatic pyrazine 5 (Fig. S1†).

### Cage-to-cage one guest (case II)

The capability of quinone 4 to promote cage disassembly opened to the investigation of a second chemical cycle in which two diamines differing in length, ethylenediamine 3 and *p*-xylylenediamine 6, are introduced in the cycle with the purpose of switching the cage size. A chemically programmed change in cage size should lead to an increased metal–metal distance, thus to a different binding capability of the system toward the same guest (calculated structures in Section S8†). In this second experiment, after the C_6_@2 formation (Fig. 1d), *p*-xylylenediamine 6 (2.5 eq. in respect to the initial aldehyde 1) was added simultaneously with quinone 4 (2.5 eq.). After 12 h, ^1^H-NMR spectra indicated a preferential conversion to the “elongated” cage C_6_@8 (Fig. 1e) which was confirmed by ESI-MS. This analysis also showed a minor amount of the architecture in which two *p*-xylylenediamine groups and one ethylenediamine are linking the TPMA units (Fig. S9†). Re-conversion to the initial cage was possible with addition of *p*-anisaldehyde 9 (2.5 eq.) and ethylenediamine 3 (2.5 eq.) with a 76% yield (Fig. 1f). In this cycle, at the beginning the C_6_ guest is bound to the cage as in Case I (4 : 1 ratio of caged and in solution), but the subsequent cage-to-cage conversion resulted in the release of the guest (1.2 : 1 ratio of caged and in solution). Also in this case, the non-quantitative yield in the re-formation of the initial structure together with the dilution resulted in a smaller uptake of C_6_ from solution (2 : 1 ratio of caged and in solution) (Section S4†). It should be highlighted that while the first part of the cycle is driven by the preference of quinone 4 to irreversibly bind ethylenediamine 3 instead of *p*-xylylenediamine 6, the following part takes advantage of the higher thermodynamic stability of the guest within cage 2.

### Competing guests (case III)

**Fig. 2:**
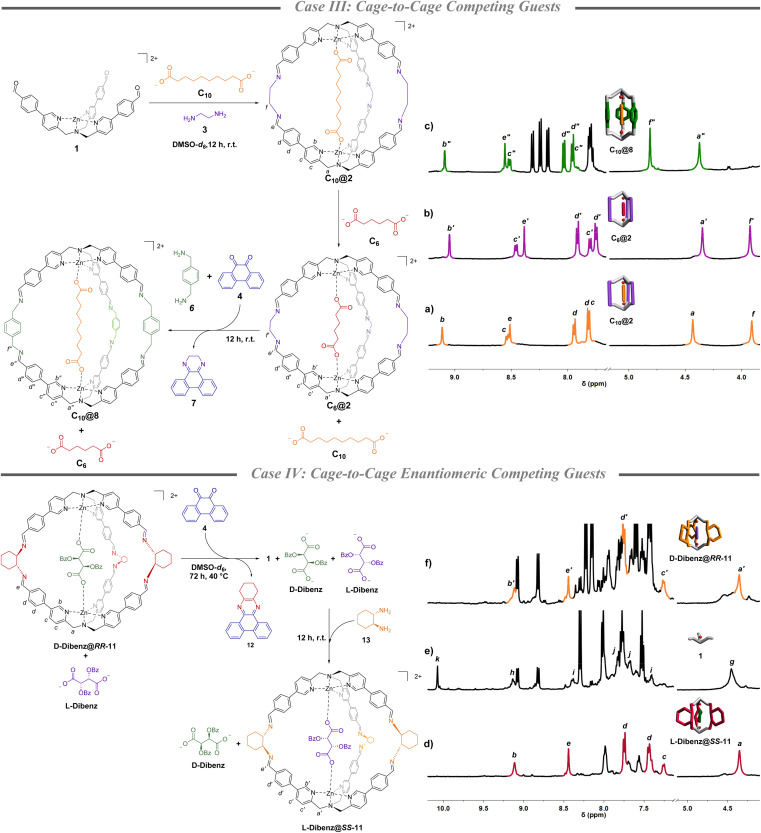
Case III: partial ^1^H-NMR (DMSO-*d*_6_, 500 MHz) of (a) C_10_@2, (b) C_6_@2 obtained after the addition of a stoichiometric amount of C_6_ to C_10_@2 with the complete exchange of the guest, and (c) C_10_@8 obtained after the addition of quinone 4 and *p*-xylylenediamine 6 to the mixture of C_6_@2 and C_10_. *p*-Xylene is used as the internal standard. Case IV: partial ^1^H-NMR (DMSO-*d*_6_, 400 MHz) of (d) d-Dibenz@*RR*-11 (d.r. 17 : 1 between the two guests), (e) complex 1 obtained from cage disassembly using quinone 4, and (f) 12 h after the addition of *S*,*S*-diaminocyclohexane 13 that led to the formation of l-Dibenz@*SS*-11 and the exchange in the selectivity for the two guests. 1,3,5-Trimethoxy benzene is used as the internal standard.

Once the methodology for cage-to-cage conversion was in hand, complete uptake and release of a guest was achieved by performing the amine subcomponent exchange in the presence of two diacids of different lengths competing in solution. Initially, C_10_@2 was quantitively formed in DMSO-*d*_6_ mixing complex 1 (1 eq.), 3 (2.5 eq.) and sebacate C_10_ (0.5 eq.) (Fig. 2a). The following addition of the adipate C_6_ (0.5 eq.) led to the complete encapsulation of the shorter diacid and the full release of C_10_. The selective displacement is a consequence of the higher binding constant of C_6_, in comparison to the longer C_10_, for cage 2.^36^ Complete guest switch (C_10_@2 to C_6_@2) was confirmed by new sets of signals in the ^1^H-NMR spectrum in the region of the benzylic protons at 4.3 ppm of the TPMA arms and the α-protons of the pyridine ring at 9.0 ppm (Fig. 2b and S10†). Subsequent addition of *p*-xylylenediamine 6 (2.5 equiv.) and quinone 4 (2.5 equiv.) led to the cage-to-cage (C_6_@2 to C_10_@4) transformation in quantitative yield as confirmed by ^1^H-NMR and ESI-MS (Fig. 2c and S11–S14†). In other words, the concomitant ethylenediamine 3 trapping resulted in the formation of cage 8 which had higher preference for the longer C_10_ dicarboxylate in virtue of an increased metal–metal distance of the cage in comparison with cage 2 (calculated structures in Section S8†).^37^

### Cage-to-cage enantiomeric competing guests (case IV)

This methodology was finally challenged in the chiral realm. The latter experiment was conceived with the idea to have enantiomeric guests competing with a cage in which chirality is inverted by subcomponent exchange. As a general strategy, we took advantage of the capability of quinone 4 to form stable adducts also with chiral 1,2-diaminocyclohexane.^42^ At the beginning of this fourth proof case, chiral cage *RR*-11 was synthesized in DMSO-*d*_6_ mixing complex 1 (1 eq.) and (*R*,*R*)-diaminocyclohexane (2.5 eq.) in the presence of a racemic mixture of l-and d-dibenzoyl tartrate (0.5 eq. each *viz.* equimolar concentration of each acid to the final cage concentration). Integration of the NMR peaks of the two diastereoisomeric inclusion complexes d-Dibenz@*RR*-11 and l-Dibenz@*RR*-11, highlighted a remarkable preference toward the d-enantiomer (d.r. 17 : 1 determined by preparing the pure diasteroisomeric systems) (Fig. 2d, S15, S24, S26, S28, and S30†). In other words, d-Dibenz@*RR*-11 is the more stable diasteroisomer in solution leaving l-Dibenz tartrate free in solution. While addition of quinone 4 (2.5 eq.) at room temperature did not result in disassembly in a reasonable time, this was achieved after 72 h by raising the temperature to 40 °C. Formation of 1 and sequestration of the diamine was confirmed by the disappearance of the imine peak at 8.5 ppm and the formation of the aldehyde peak at 10 ppm (Fig. 2e). Subsequent addition of *S*,*S*-diaminocyclohexane 13 (2.5 eq.) drove the reaction mixture toward the formation of the enantiomeric cage *SS*-11 and preferential sequestration of l-Dibenz, to form preferentially l-Dibenz@*SS*-11 in 60% yield (Fig. 2f and S16–S19†). The lower yield reported in this case can be associated with the decomplexation of TPMA structures by competitive binders in solution.^43^ However, to the best of our knowledge, this represents the first example in which programmed uptake and release of two enantiomeric molecules within a confined system can be selectively achieved.

## Conclusions

In conclusion, in this study, we conceived a novel quinone based strategy for selective guest release and uptake which is based either on cage disassembly/assembly or cage to cage conversions. Guest uptake and release has been successfully optimized allowing: (i) reversible assembly and disassembly of the cage with release and uptake of the guest, (ii) cage to cage conversions with switch in affinity toward guests of different sizes, and (iii) inversion in cage chirality with the concomitant and selective uptake and release of two enantiomeric dicarboxylates. Due to the wide use of imine DCC methodology in the formation of supramolecular architectures, this methodology could pave the way to the selective release and uptake in other architectures that take advantage of diamine DCC chemistry.

## Data availability

All experimental procedures, characterisation data and optimised coordinates are available in the ESI.†

## Author contributions

C. Z. conceived the project. F. B. performed the experimental work. F. B. and C. Z. analyzed the experimental results. All of the authors contributed to the scientific discussions and manuscript writing.

## Conflicts of interest

There are no conflicts to declare.

## Supplementary Material

SC-014-D3SC01368E-s001
