# Supplementary material for: Programmed guest confinement via hierarchical cage to cage transformations
Source: Chem Sci. 2023 Jul 4;14(30):8147–51. doi: 10.1039/d3sc01368e (PMC10395264; doi:10.1039/d3sc01368e)
Supplement: SC-014-D3SC01368E-s001 [file SC-014-D3SC01368E-s001.pdf]

Electronic Supplementary Material (ESI) for Chemical Science.
This journal is © The Royal Society of Chemistry 2023

Supporting Information for

Programmed Guest Confinement via Hierarchical Cage to Cage Transformations

Authors: Federico Begato,[1] Giulia M. Licini,[1] and Cristiano Zonta[1]*

[1] Department of Chemical Sciences, University of Padova, via Marzolo 1, 35131 Padova

*Correspondence to: cristiano.zonta@unipd.it

Contents

Supporting Information for 1
1 General Methods 3
2 Synthesis and Characterization 4
2.1 Synthesis of Cages R@2 4
2.1.1 C_6@2 4
2.1.2 C_{10}@2 4
2.2 Synthesis of Cages R@8 5
2.2.1 C_6@8 5
2.2.2 C_{10}@8 5
2.3 Synthesis of Cages R@*RR*-11 6
2.3.1 D-DIBENZ@*RR*-11 6
2.3.2 L-DIBENZ@*RR*-11 6
2.4 Synthesis of Cages R@*SS*-11 7
2.4.1 L-DIBENZ@*SS*-11 7
2.4.2 D-DIBENZ@*SS*-11 7
3 Disassembly and Assembly cycle of cage C_6@2 using 4 8
4 Cage to Cage conversion with 6 11
5 Cage-to-Cage Competing Guests 16
5.1 Guest substitution from C_{10} to C_6 16
5.2 Cage to Cage conversion with selective release and uptake of the guests 17
6 Chiral cage Assembly-Disassembly-Assembly with enantiomers 20
6.1 Selective encapsulation using a racemic mixture 20
6.2 Cage-to-Cage Enantiomeric Competing Guests 21
7 NMR and ESI characterizations 24
7.1 C_6@2 24

7.2 C_{10}@2 24
7.3 C_6@8 25
7.4 C_{10}@8 25
7.5 L-Dibenz@*RR*-11 26
7.6 D-Dibenz@*RR*-11 27
7.7 L-Dibenz@*SS*-11 28
7.8 D-Dibenz@*SS*-11 29
7.9 ESI-MS spectrum of cage D-Dibenz@*RR*-11 30
7.10 ESI-MS spectrum of cage L-Dibenz@*SS*-11 31
8 Computational Studies 32
9 Coordinates of the optimized structures 33
9.1 C_6@2 33
9.2 C10@2 37
9.3 C_6@8 41
9.4 C10@8 45
10 References 51

1 General Methods

NMR spectra were recorded at 301 K on Bruker 400 Avance III BBi-z grad 5 mm and Bruker Avance-500 MHz, All the ^{1}H-NMR spectra were referenced to residual isotopic impurity of DMSO-d_6 (2.50 ppm). The following abbreviations are used in reporting the multiplicity for NMR resonances; s=single, d=doublet, t= triplet, and m=multiplet. The NMR data were processed using MestReNova 12.0.0.

ESI-MS spectra have been acquired with an Agilent Technology LC/MSD Trap SL, interfaced to an Agilent 1100 binary pump. The samples were preventively diluted in acetonitrile and then injected via direct infusion with a syringe pump at a rate of 0.05 ml/min. MS peak intensity for each analysis is reported as monoisotopic mass and the data were processed with MestReNova 12.0.0. Theoretical isotopic pattern have been simulated with enviPat web (https://www.envipat.eawag.ch).

The computational searches of the most stable structures and the TD-DFT calculations were carried out Gaussian 16 package[1] Revision C.01 and processed with GaussView 6.0.16[2] or CYL view BETA 1.0.

Chemicals were purchased from Merck, TCI, or Apollo Scientific and used without further purification.

2 Synthesis and Characterization

2.1 Synthesis of Cages R@2

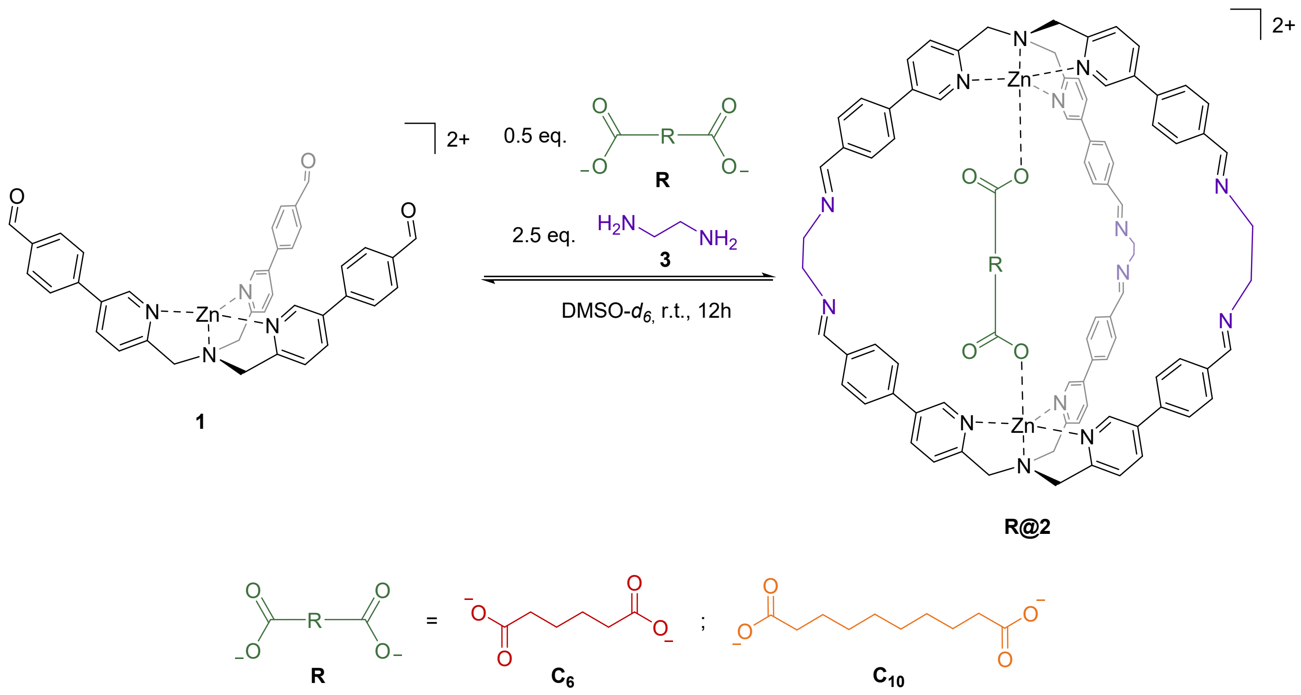

General procedure for the synthesis of molecular cages **R@2**.[1] Perchlorate counterions are removed for clarity.

To 500 µL (1.0 µmol) of a solution 0.002 M of the aldehyde zinc complex **1** in DMSO-d_6, 25 µL (0.5 µmol) of a solution 0.02 M in DMSO-d_6 of a dicarboxylic acid **R** and 125 µL (2.5 µmol) of a solution 0.02 M in DMSO-d_6 of ethylenediamine **3** were added in a NMR tube. The mixture was left overnight at room temperature and checked *via* ^{1}H-NMR.

2.1.1 C$_6$@2

yield=95%, determined *via* ^{1}H-NMR on internal standard *p*-xylene.

^{1}H-NMR (400 MHz, DMSO-d_6) δ (ppm): 9.04 (s, 6H, PyrH), 8.46 (d, *J* = 8.2, 6H, PyrH), 8.38 (s, 6H, NH$_{imm}$), 7.91 (d, *J* = 8.0 Hz, 12H, ArH), 7.80 (d, *J* = 8.3 Hz, 6H, PyrH), 7.75 (d, *J* = 8.0 Hz, 12H, ArH), 4.35 (s, 12H, CH$_2$-$_{TPMA}$), 3.93 (s, 12H, CH$_2$-$_{EDA}$), 1.78 – 1.74 (m, 4H, CH$_{2\text{-acid}}$) (4H, CH$_{2\text{-acid,}}$ hide by solvent).
MS (ESI-MS) (*m/z*): [M^{2+}] calcd. for [$C_{90}H_{80}N_{14}O_4Zn_2$]$^{2+}$, 774.25; found 774.23

2.1.2 C$_{10}$@2

yield=94%, determined *via* ^{1}H-NMR on internal standard *p*-xylene.

^{1}H-NMR (500 MHz, DMSO-d_6) δ (ppm): 9.11 (s, 6H, PyrH), 8.54 (d, *J* = 8.3, 6H, PyrH), 8.51 (s, 6H, NH$_{imm}$), 7.94 (d, *J* = 8.0 Hz, 12H, ArH), 7.82 – 7.81 (m, 18H, PyrH + ArH), 4.43 (s, 12H, CH$_2$-$_{TPMA}$), 3.91 (s, 12H , CH$_2$-$_{EDA}$), 1.88 – 1.82 (m, 6H, CH$_{2\text{-acid}}$), 1.60 – 1.54 (m, 6H, CH$_{2\text{-acid}}$), 1.49 – 1.40 (m, 6H, CH$_{2\text{-acid}}$)
MS (ESI-MS) (*m/z*): [M^{2+}] calcd. for [$C_{94}H_{88}N_{14}O_4Zn_2$]$^{2+}$, 804.28; found 804.31

2.2 Synthesis of Cages R@8

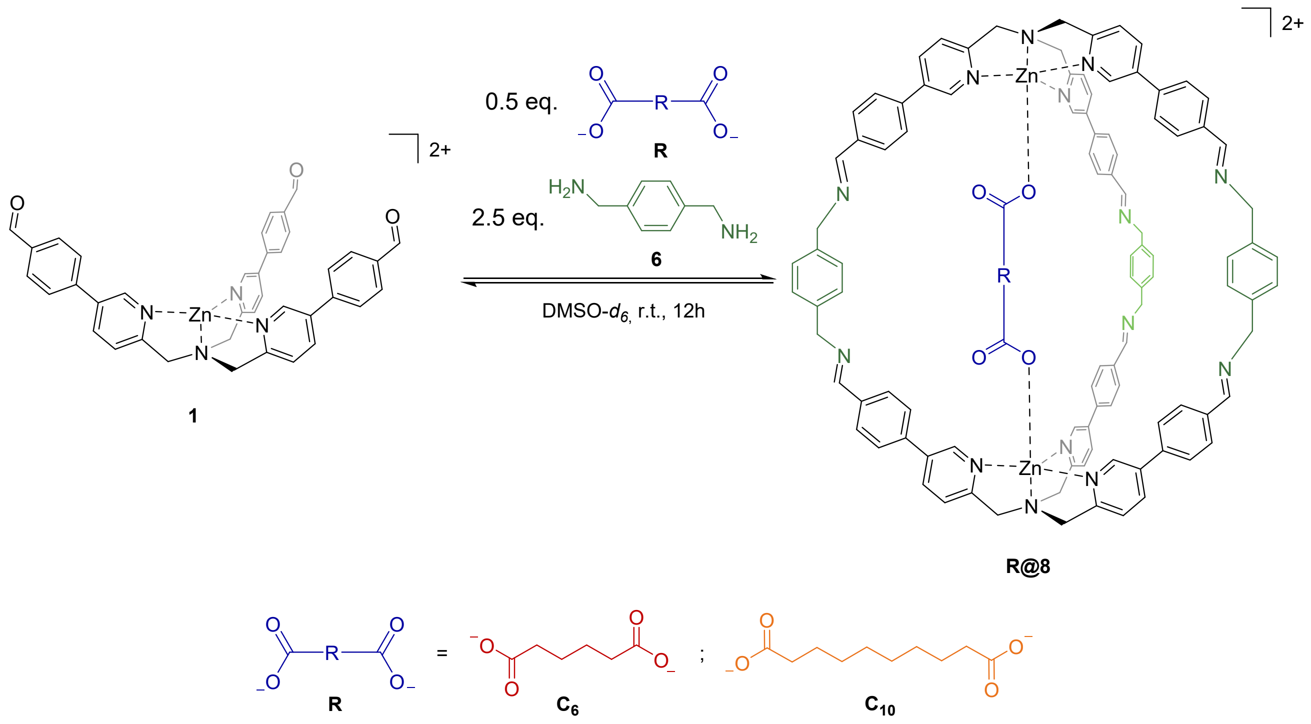

General procedure for the synthesis of molecular cages **R@8**.[3] Perchlorate counterions are removed for clarity.

To 500 µL (1.0 µmol) of a solution 0.002 M of the aldehyde zinc complex **1** in DMSO-d_6, 25 µL (0.5 µmol) of a solution 0.02 M in DMSO-d_6 of dicarboxylate **R** and 125 µL (2.5 µmol) of a solution 0.02 M in DMSO-d_6 of *p*-xylylenediamine **6** were added in a NMR tube. The mixture was left overnight at room temperature and checked *via* ^{1}H-NMR.

2.2.1 C_6@8

yield=93%, determined *via* ^{1}H-NMR on internal standard *p*-xylene.

^{1}H-NMR (400 MHz, DMSO-d_6) δ (ppm): 9.33 – 8.89 (m, 6H, PyrH), 8.63 – 8.35 (m, 12H, NH_{imm}+ PyrH), 8.09 – 7.84 (m, 12H, ArH), 7.77 (m, 18H, PyrH + ArH), 7.38 (s, 12H, $ArH_{p\text{-}xylDA}$), 4.79 (m, 12H, $CH_{2\text{-}pxylDA}$), 4.49 – 4.06 (m, 12H, $CH_{2\text{-}TPMA}$),1.98 – 1.84 (m, 6H, $CH_{2\text{-}acid}$), 1.25 – 1.18 (m, 2H, $CH_{2\text{-}acid}$).
MS (ESI-MS) (*m/z*): [(M^{2+}-C_6)+2Cl^-] calcd. for $[C_{102}H_{84}Cl_2N_{14}Zn_2]^{2+}$, 853.24; found 853.27

2.2.2 C_{10}@8

yield=96%, determined *via* ^{1}H-NMR on internal standard *p*-xylene.

^{1}H-NMR (400 MHz, DMSO-d_6) δ (ppm): 9.09 (s, 6H, PyrH), 8.56 (s, 6H, NH_{imm}), 8.52 (d, *J* = 8.2, 6H, PyrH), 7.96 (d, *J* = 8.0 Hz, 12H, ArH), 7.88 – 7.62 (m, 18H, PyrH + ArH), 7.36 (s, 12H, $ArH_{p\text{-}xylDA}$), 4.81 (s, 12H, $CH_{2\text{-}pxylDA}$), 4.38 (s, 12H, $CH_{2\text{-}TPMA}$), 1.76 – 1.72 (m, 6H, $CH_{2\text{-}acid}$), 1.35 – 1.23 (m, 12H, $CH_{2\text{-}acid}$).
MS (ESI-MS) (*m/z*): [M^{2+}] calcd. for $[C_{112}H_{100}N_{14}O_4Zn_2]^{2+}$, 918.33; found 918.38

2.3 Synthesis of Cages R@*RR*-11

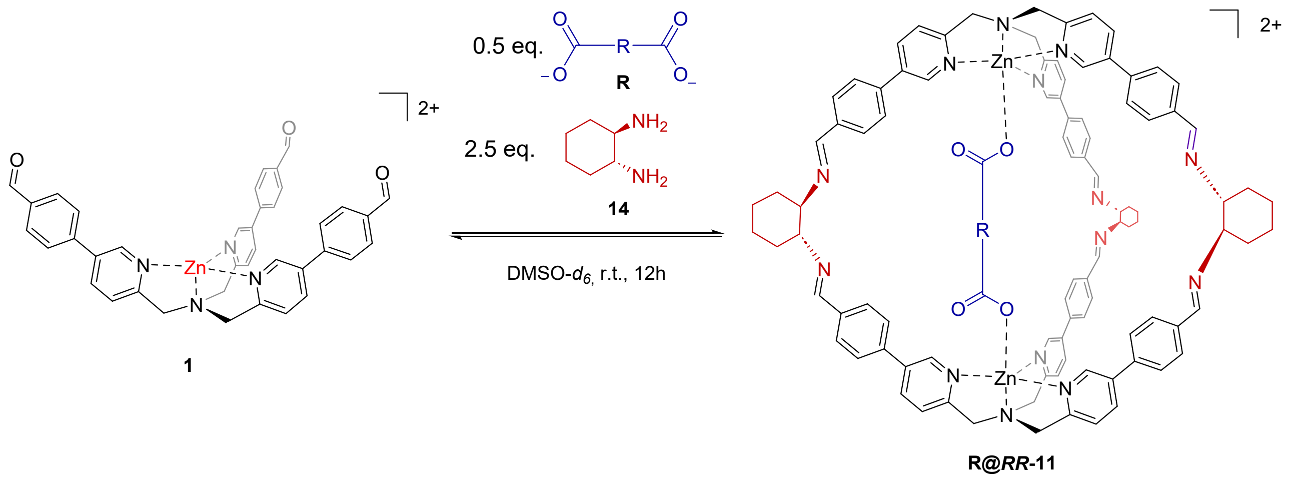

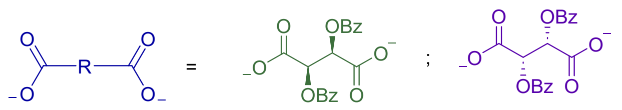

R **D-Dibenz** **L-Dibenz**

General procedure for the synthesis of molecular cages **R@*RR*-11**. Perchlorate counterions are removed for clarity.

To 500 µL (1.0 µmol) of a solution 0.002 M of the aldehyde zinc complex **2** in DMSO-d_6, 50 µL (0.5 µmol) of a solution 0.02 M in DMSO-d_6 of dicarboxylate **R** and 125 µL (2.5 µmol) of a solution 0.02 M in DMSO-d_6 of *R*,*R*-cyclohexyldiamine **14** were added in a NMR tube. The mixture was left overnight at room temperature and checked *via* ^{1}H-NMR.

2.3.1 D-DIBENZ@*RR*-11

yield=94%, determined *via* ^{1}H-NMR on internal standard 1,3,5-trimethoxybenzene.

^{1}H-NMR (400 MHz, DMSO-d_6) δ (ppm): 9.14 (s, 6H, PyrH), 8.46 (s, 6H, NH_{imm}), 8.01 (d, *J* = 8.0 Hz, 6H, PyrH), 7.77 (d, *J* = 7.9 Hz, 12H, ArH), 7.48z – 7.39 (m, 18H, ArH + PyrH), 7.28 (d, *J* = 7.3 Hz, 4H, $ArH_{benzoyl}$), 7.03 – 6.99 (m, 2H, $ArH_{benzoyl}$), 6.56 – 6.52 (m, 4H), 6.42 (s, 2H, $CH_{benzoyl}$), 4.38 (s, 12H, CH_2-$_{TPMA}$), 1.88 – 1.83 (m, 6H, CH_2-$_{cyclohexDA}$), 1.69 – 1.64 (m, 6H, CH_2-$_{cyclohexDA}$), 1.52 – 1.49 (m, 6H, CH_2-$_{cyclohexDA}$), 1.18 – 1.14 (m, 6H, CH_2-$_{cyclohexDA}$).
MS (ESI-MS) (*m/z*): [M^{2+}] calcd. for $[C_{114}H_{102}N_{14}O_8Zn_2]^{2+}$, 963.33; found 963.36

2.3.2 L-DIBENZ@*RR*-11

yield=93%, determined *via* ^{1}H-NMR on internal standard 1,3,5-trimethoxybenzene.

^{1}H-NMR (400 MHz, DMSO-d_6) δ (ppm): 9.02 (s, 6H, PyrH), 8.31 (s, 6H, NH_{imm}), 8.08 (d, J = 8.1 Hz, 6H, PyrH), 7.71 – 7.65 (m, 16H, ArH + $ArH_{benzoyl}$), 7.52 (d, J = 8.2 Hz, 6H, PyrH), 7.38 – 7.36 (m, 14H, ArH + $ArH_{benzoyl}$), 7.01 – 6.97 (m, 4H, $ArH_{benzoyl}$), 6.29 (s, 2H, $CH_{benzoyl}$), 4.38 (m, 12H, CH_2-$_{TPMA}$), 1.88 – 1.84 (m, 6H, CH_2-$_{cyclohexDA}$), 1.68 – 1.63 (m, 6H, CH_2-$_{cyclohexDA}$), 1.52 – 1.49 (m, 6H, CH_2-$_{cyclohexDA}$), 1.19 – 1.14 (m, 6H, CH_2-$_{cyclohexDA}$).
MS (ESI-MS) (*m/z*): [M^{2+}] calcd. for $[C_{114}H_{102}N_{14}O_8Zn_2]^{2+}$, 963.33; found 963.36

2.4 Synthesis of Cages R@*SS*-11

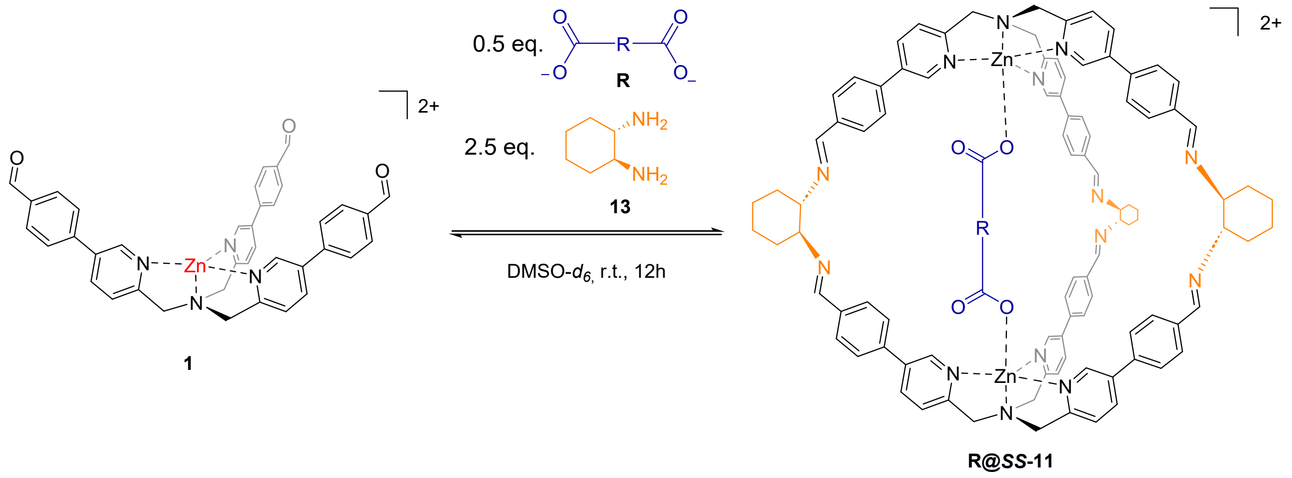

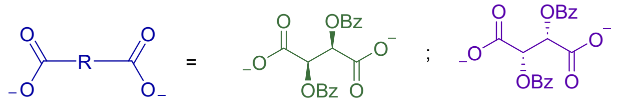

R **D-Dibenz** **L-Dibenz**

General procedure for the synthesis of molecular cages **R@*SS*-11**. Perchlorate counterions are removed for clarity.

To 500 µL (1.0 µmol) of a solution 0.002 M of the aldehyde zinc complex **2** in DMSO-d_6, 50 µL (0.5 µmol) of a solution 0.02 M in DMSO-d_6 of dicarboxylate **R** and 125 µL (2.5 µmol) of a solution 0.02 M in DMSO-d_6 of *S*,*S*-cyclohexyldiamine **13** were added in a NMR tube. The mixture was left overnight at room temperature and checked *via* ^{1}H-NMR.

2.4.1 L-DIBENZ@*SS*-11

yield=94%, determined *via* ^{1}H-NMR on internal standard 1,3,5-trimethoxybenzene.

^{1}H-NMR (400 MHz, DMSO-d_6) δ (ppm): 9.14 (s, 6H, PyrH), 8.46 (s, 6H, NH_{imm}), 8.01 (d, *J* = 8.0 Hz, 6H, PyrH), 7.77 (d, *J* = 7.9 Hz, 12H, ArH), 7.48z – 7.39 (m, 18H, ArH + PyrH), 7.28 (d, *J* = 7.3 Hz, 4H, $ArH_{benzoyl}$), 7.03 – 6.99 (m, 2H, $ArH_{benzoyl}$), 6.56 – 6.52 (m, 4H), 6.42 (s, 2H, $CH_{benzoyl}$), 4.38 (s, 12H, CH_2-$_{TPMA}$), 1.88 – 1.83 (m, 6H, CH_2-$_{cyclohexDA}$), 1.69 – 1.64 (m, 6H, CH_2-$_{cyclohexDA}$), 1.52 – 1.49 (m, 6H, CH_2-$_{cyclohexDA}$), 1.18 – 1.14 (m, 6H, CH_2-$_{cyclohexDA}$).
MS (ESI-MS) (*m/z*): [M^{2+}] calcd. for $[C_{114}H_{102}N_{14}O_8Zn_2]^{2+}$, 963.33; found 963.36

2.4.2 D-DIBENZ@*SS*-11

yield=93%, determined *via* ^{1}H-NMR on internal standard 1,3,5-trimethoxybenzene.

^{1}H-NMR (400 MHz, DMSO-d_6) δ (ppm): 9.02 (s, 6H, PyrH), 8.31 (s, 6H, NH_{imm}), 8.08 (d, J = 8.1 Hz, 6H, PyrH), 7.71 – 7.65 (m, 16H, ArH + $ArH_{benzoyl}$), 7.52 (d, J = 8.2 Hz, 6H, PyrH), 7.38 – 7.36 (m, 14H, ArH + $ArH_{benzoyl}$), 7.01 – 6.97 (m, 4H, $ArH_{benzoyl}$), 6.29 (s, 2H, $CH_{benzoyl}$), 4.38 (m, 12H, CH_2-$_{TPMA}$), 1.88 – 1.84 (m, 6H, CH_2-$_{cyclohexDA}$), 1.68 – 1.63 (m, 6H, CH_2-$_{cyclohexDA}$), 1.52 – 1.49 (m, 6H, CH_2-$_{cyclohexDA}$), 1.19 – 1.14 (m, 6H, CH_2-$_{cyclohexDA}$).
MS (ESI-MS) (*m/z*): [M^{2+}] calcd. for $[C_{114}H_{102}N_{14}O_8Zn_2]^{2+}$, 963.33; found 963.36

3 Disassembly and Assembly cycle of cage C_6@2 using 4

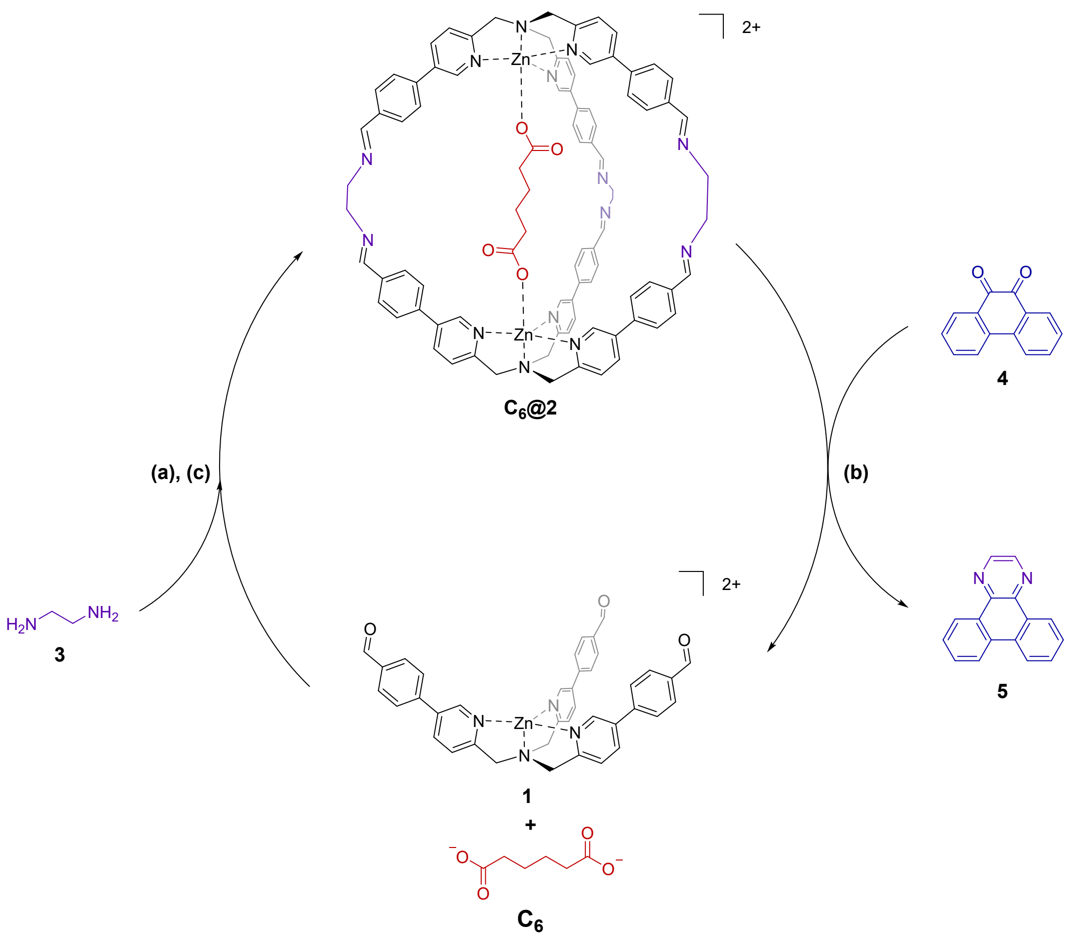

Procedure for the assembly/disassembly cycle. Perchlorate counterions are removed for clarity.

a) Formation of cage **C_6@2**:

To 350 µL (0.70 µmol) of a solution 0.002 M of complex **1** in DMSO-d_6, 18 µL (0.35 µmol) of a solution 0.02 M in DMSO-d_6 of adipate **C_6,** 87 µL (1.75 µmol) of a solution 0.02 M in DMSO-d_6 of ethylenediamine, and 30 µL (0.60 µmol) of a solution 0.02 M in DMSO-d_6 of 1,3,5-trimethoxybenzene were added in a NMR tube and left at room temperature for 12 hours. After 12 hours, ^{1}H-NMR confirmed the complete formation of cage **C_6@2** (yield 95% determined *via* ^{1}H-NMR on internal standard 1,3,5-trimethoxybenzene).

b) Disassembly of cage **C_6@2**with **4**:

After the complete formation of cage **C_6@2**, 44 µL (1.75 µmol) of a solution 0.04 M in DMSO-d_6 of 1,3-phenantrenequinone **4**, and 20 µL of H_2O were added to the NMR tube and the mixture was heated at 60 °C for 48 h. ^{1}H-NMR confirmed the complete disassembly of the cage as confirmed by the disappearance of the imine peak at 8.4 ppm and the formation of the aldehyde peak of complex **1** at 10 ppm.

c) Assembly of cage **C_6@2** with ethylenediamine:

After 48 h, 87 µL (1.75 µmol) of a solution 0.02 M in DMSO-d_6 of ethylenediamine were added to the NMR tube and the mixture was left for 12 hours at room temperature. ^{1}H-NMR confirmed the formation of cage **C_6@2** (yield 90% determined *via* ^{1}H-NMR on internal standard 1,3,5-trimethoxybenzene).

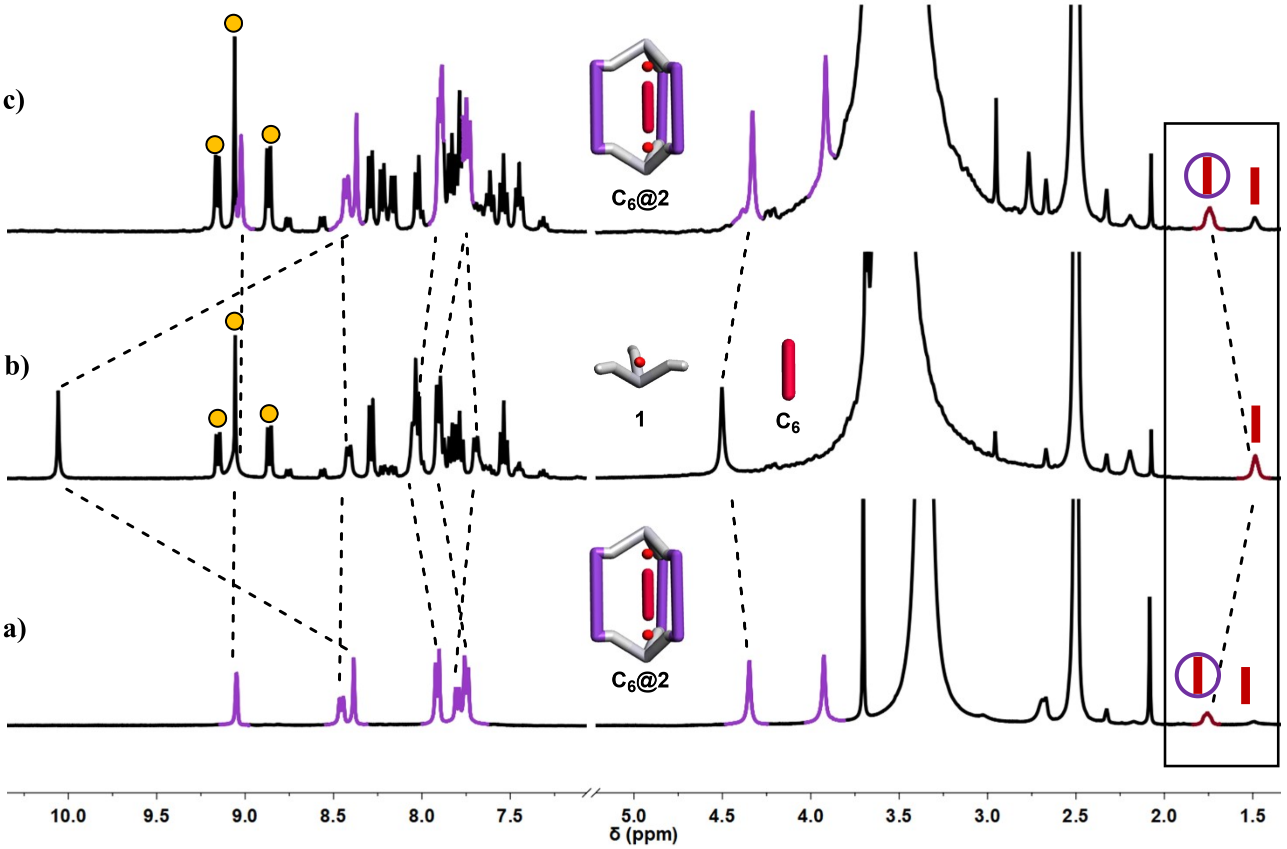

Figure S1 ^{1}H-NMR (DMSO-d_6, 400 MHz) of a) cage **C$_6$@2**, b) 2 days after the addition of **4** and 20 μL of H_2O, heating the solution at 60 °C, and c) 12 h after the addition of ethylenediamine. The purple circle indicates cage **2**, red stick indicates **C$_6$**. Yellow dots correspond to the protons of pyrazine **5**.

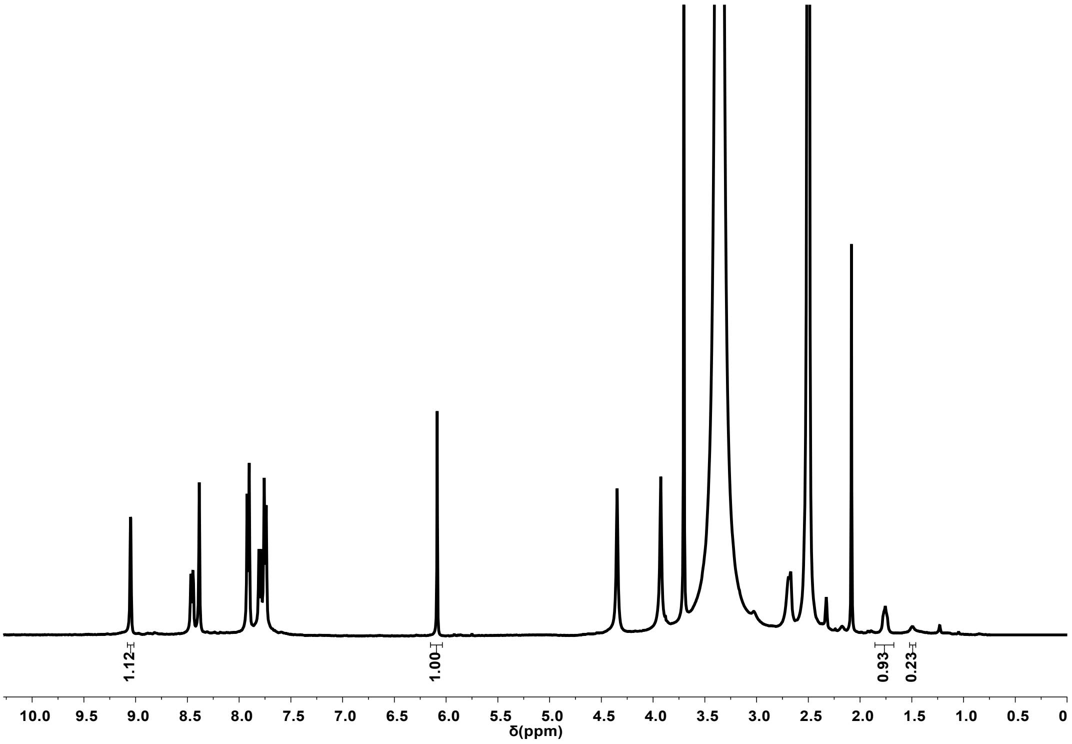

Figure S2 ^{1}H-NMR (DMSO-d_6, 400 MHz) cage **C$_6$@2** (yield=95%, based on internal standard 1,3,5-trimethoxybenzene at 6.1 ppm). 4:1 ratio among encapsulated and free **C$_6$** dicarboxylate.

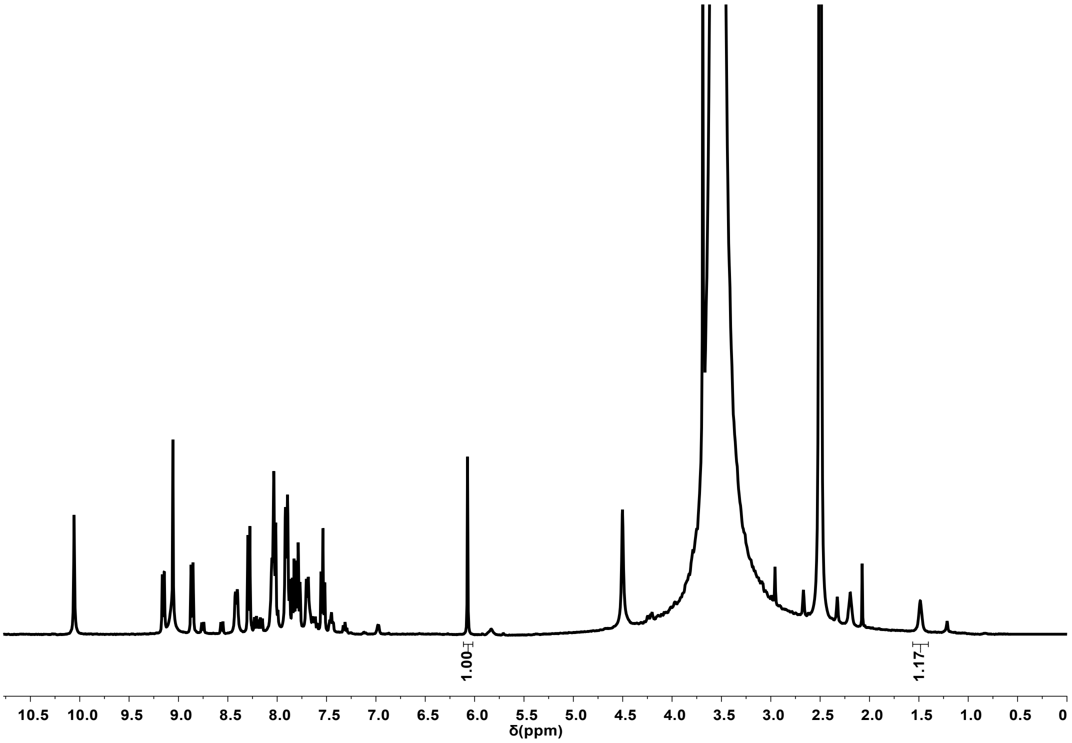

Figure S3 ^{1}H-NMR (DMSO-d_6, 400 MHz) cage $\mathbf{C_6@2}$ 2 days after the addition of **4**, 20 μL of H_2O, and heating the solution at 60 °C. Complete release of the guest $\mathbf{C_6}$ was observed. Peaks between 8.30-8.10 ppm belong to compound **7**. Peaks between 8.80-8.60 ppm are due to the formation of a side product between the quinone and ethylenediamine, probably a dimer.[4]

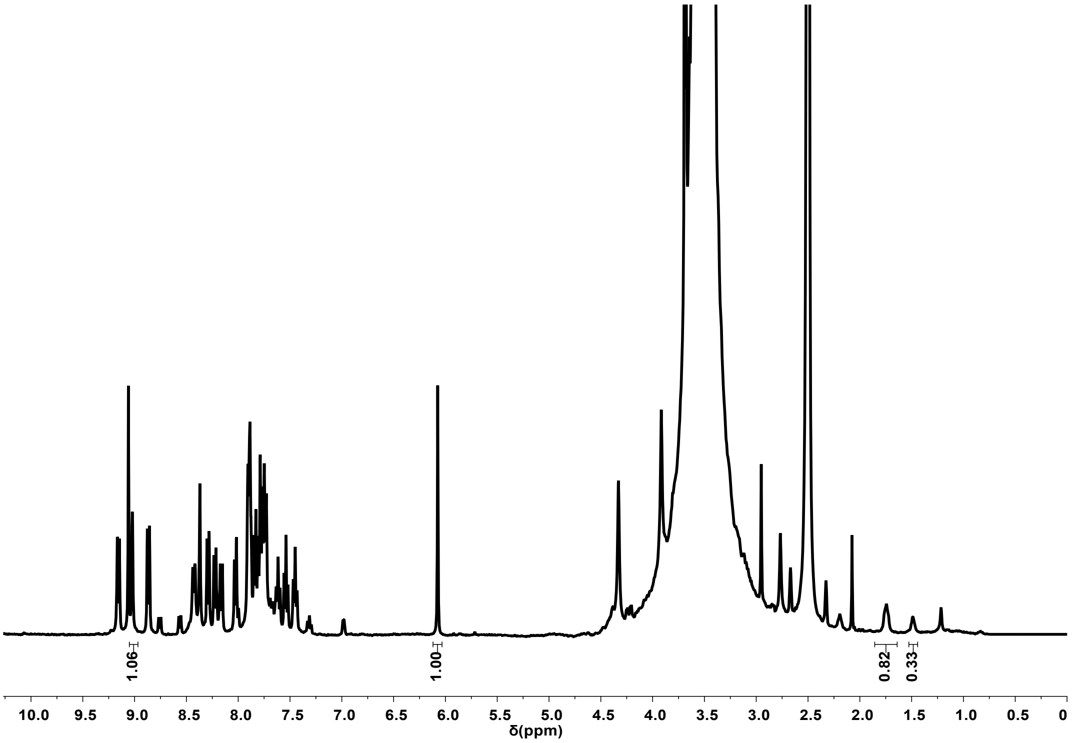

Figure S4 ^{1}H-NMR (DMSO-d_6, 400 MHz) of cage $\mathbf{C_6@2}$ 12 h after the addition of ethylenediamine to the mixture of the previously disassembled cage (yield=90%, based on internal standard 1,3,5-trimethoxybenzene at 6.1 ppm). 2.5:1 ratio among encapsulated and free $\mathbf{C_6}$ dicarboxylate.

4 Cage to Cage conversion with 6

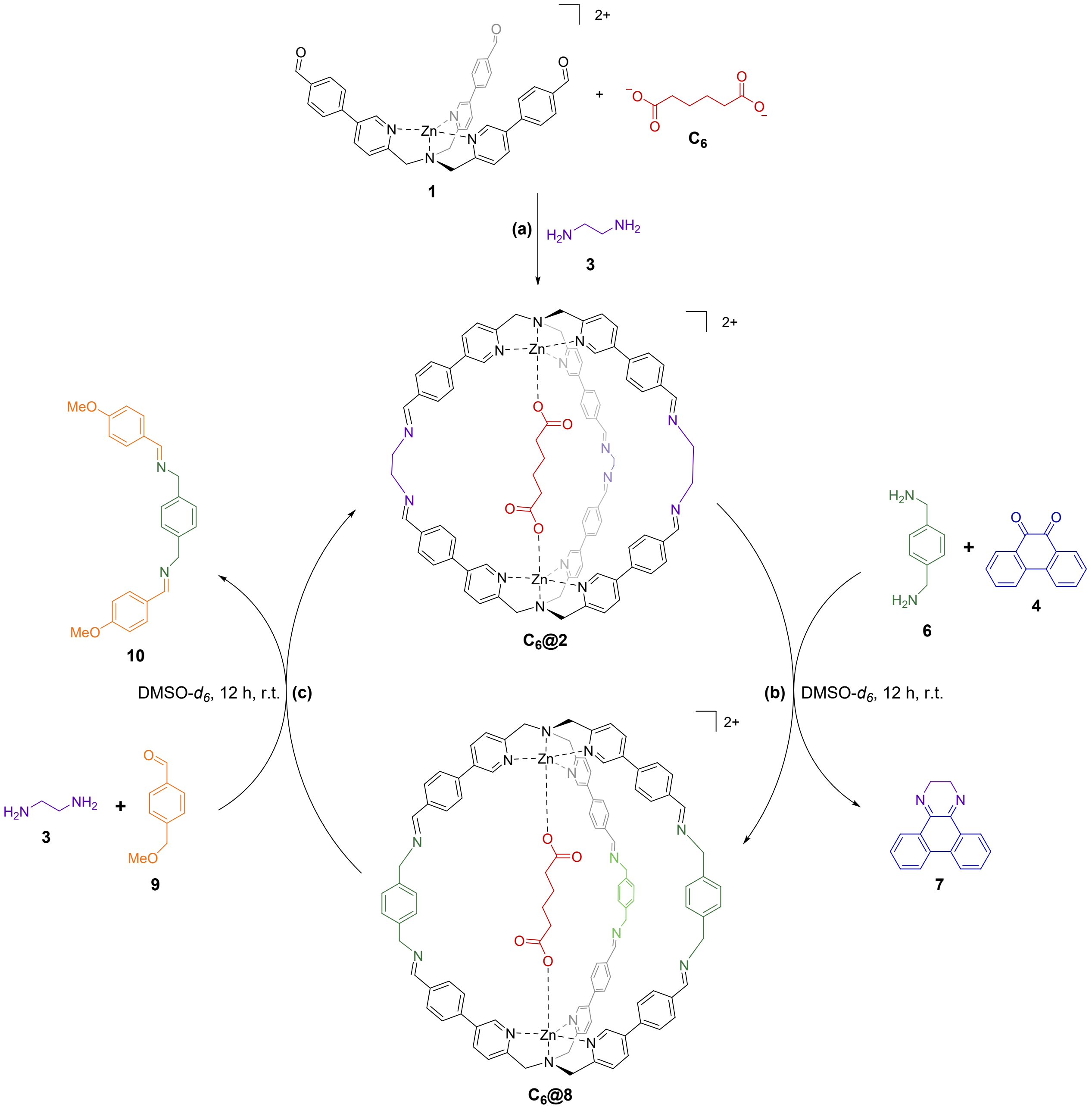

Procedure for the cage to cage transformation. Perchlorate counterions are removed for clarity.

a) Formation of cage **C_6@2**:

To 350 µL (0.70 µmol) of a solution 0.002 M of complex **1** in DMSO-d_6, 18 µL (0.35 µmol) of a solution 0.02 M in DMSO-d_6 of a adipate **C_6**, 87 µL (1.75 µmol) of a solution 0.02 M in DMSO-d_6 of ethylenediamine, and 30 µL (0.37 µmol) of a solution 0.012 M in DMSO-d_6 of *p*-xylene were added in a NMR tube and left at room temperature for 12 hours. After 12 hours, ^{1}H-NMR confirmed the complete formation of cage **C_6@2** (yield 95% determined *via* ^{1}H-NMR on internal standard *p*-xylene).

b) Cage to cage conversion from **C_6@2 to C_6@8** with **4**:

After the complete formation of cage **C_6@2**, 87 µL (1.75 µmol) of a solution 0.02 M in DMSO-d_6 of *p*-xylylenediamine and 44 µL (1.75 µmol) of a solution 0.04 M in DMSO-d_6 of 1,3-phenantrenequinone **4** were added to the NMR tube and the mixture was left for 12 hours at room temperature. After 12 hours, ^{1}H-NMR confirmed the formation of cage **C_6@8** (ESI analysis showed the presence of a minor species in which one arm was substituted with ethylenediamine instead of *p*-xylylenediamine).

c) Cage to cage conversion from **C_6@8** to **C_6@2**:

After the formation of cage **C_6@8**, 87 µL (1.75 µmol) of a solution 0.02 M in DMSO-d_6 of ethylenediamine and 44 µL (1.75 µmol) of a solution 0.04 M in DMSO-d_6 of *p*-methoxybenzaldehyde **9** were added to the NMR tube and the mixture was left for 12 hours at room temperature. After this time, ^{1}H-NMR confirmed the re-formation of cage **C_6@2** (yield 76% determined *via* ^{1}H-NMR on internal standard *p*-xylene).

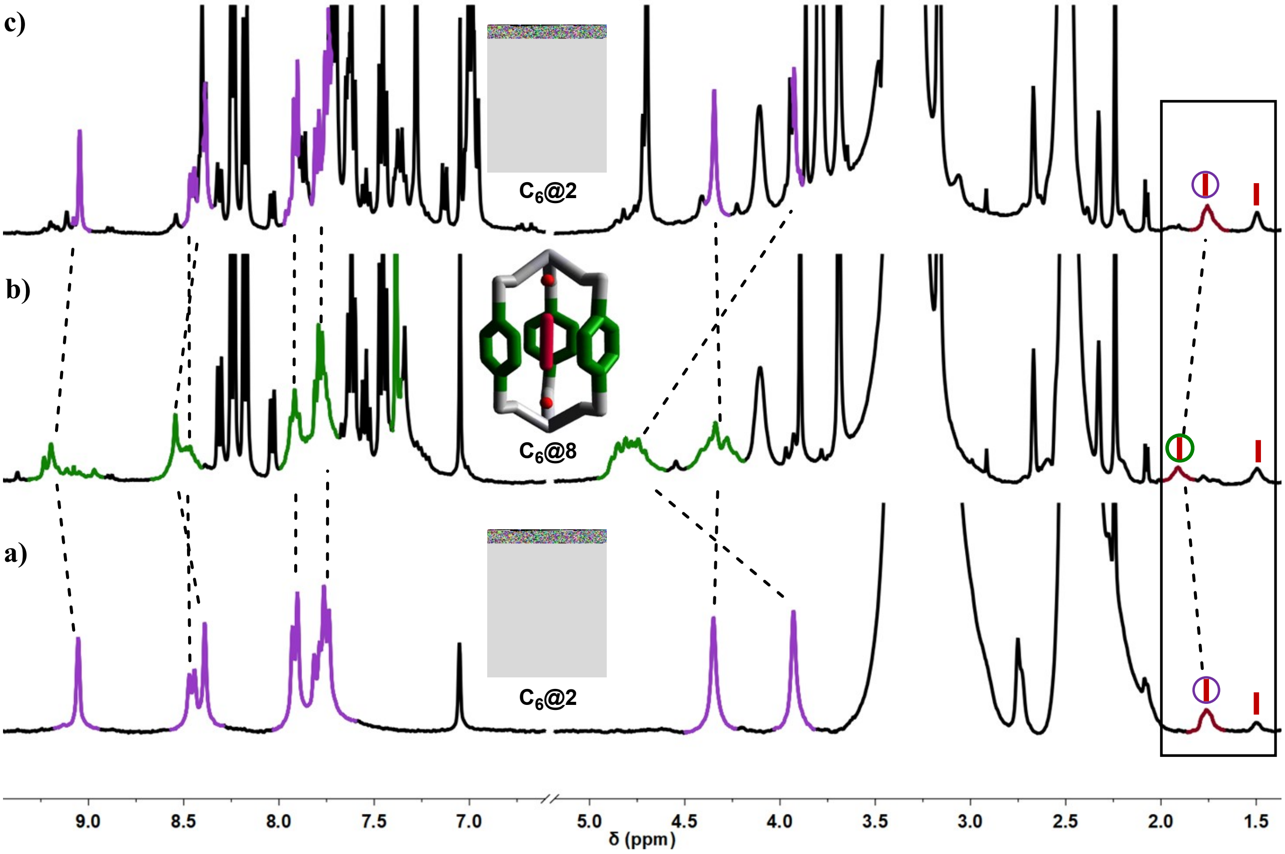

Figure S5 ^{1}H-NMR (DMSO-d_6, 400 MHz) of a) cage **C_6@2**, b) 12 hours after the addition of *p*-xylylenediamine **6** and quinone **4** that led to the formation of cage **C_6@8**, and c) 12 h after the addition of ethylenediamine and *p*-methoxybenzaldehyde **9** that allow the re-formation of cage **C_6@2**. The purple circle indicates cage **2**, the green circle indicates cage **8**, red stick indicates **C_6**.

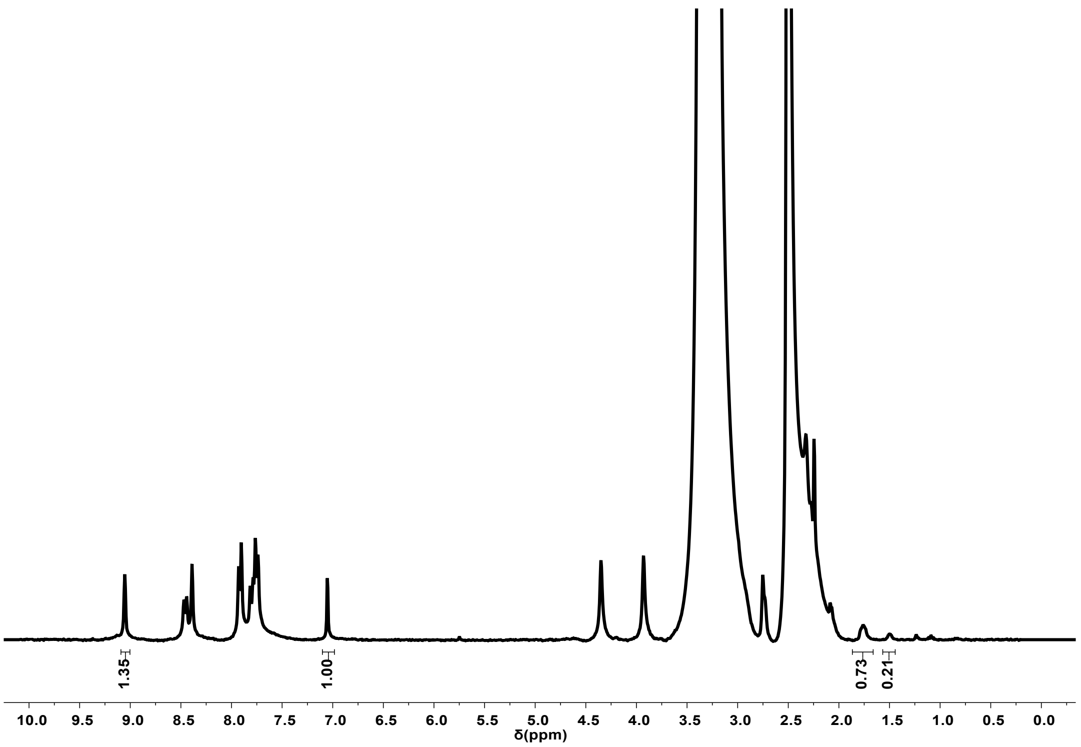

Figure S6 ^{1}H-NMR (DMSO-d_6, 400 MHz) of cage **C_6@2** (yield=95%, based on internal standard *p*-xylene at 7.1 ppm). 4:1 ratio among encapsulated and free **C_6** dicarboxylate.

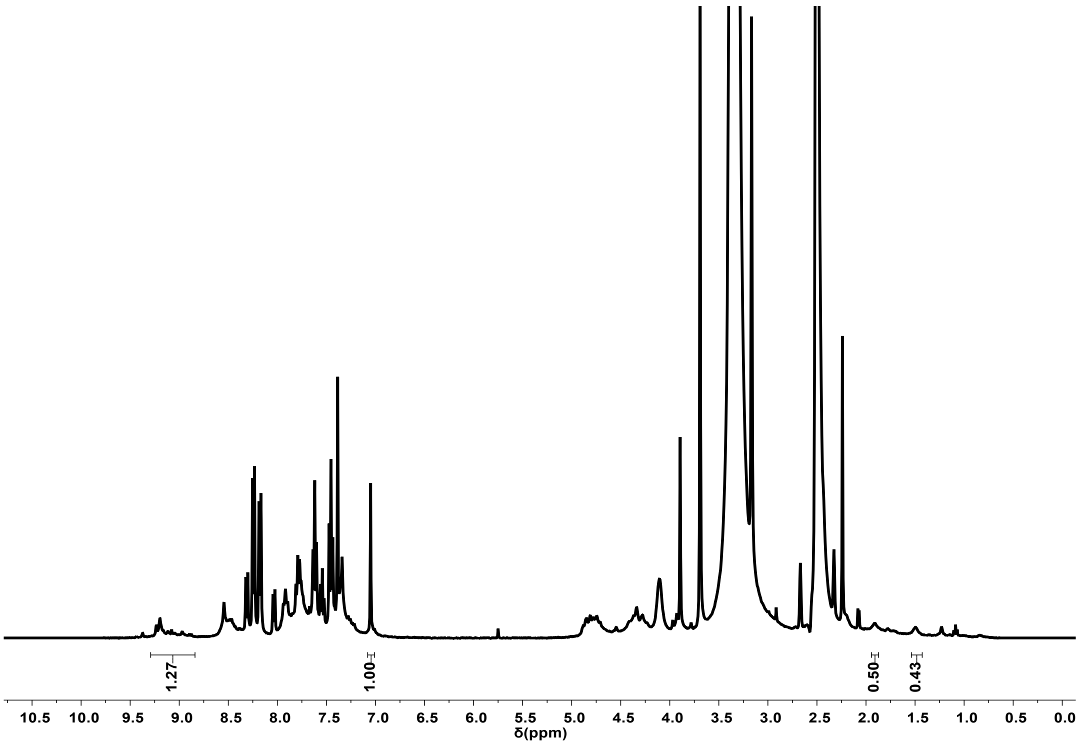

Figure S7 ^{1}H-NMR (DMSO-d_6, 400 MHz) of cage **C_6@2** 12 hours after the addition of *p*-xylylenediamine **6** and **4** that led to the formation of cage **C_6@8**. 1.2:1 ratio among encapsulated and free **C_6** dicarboxylate.

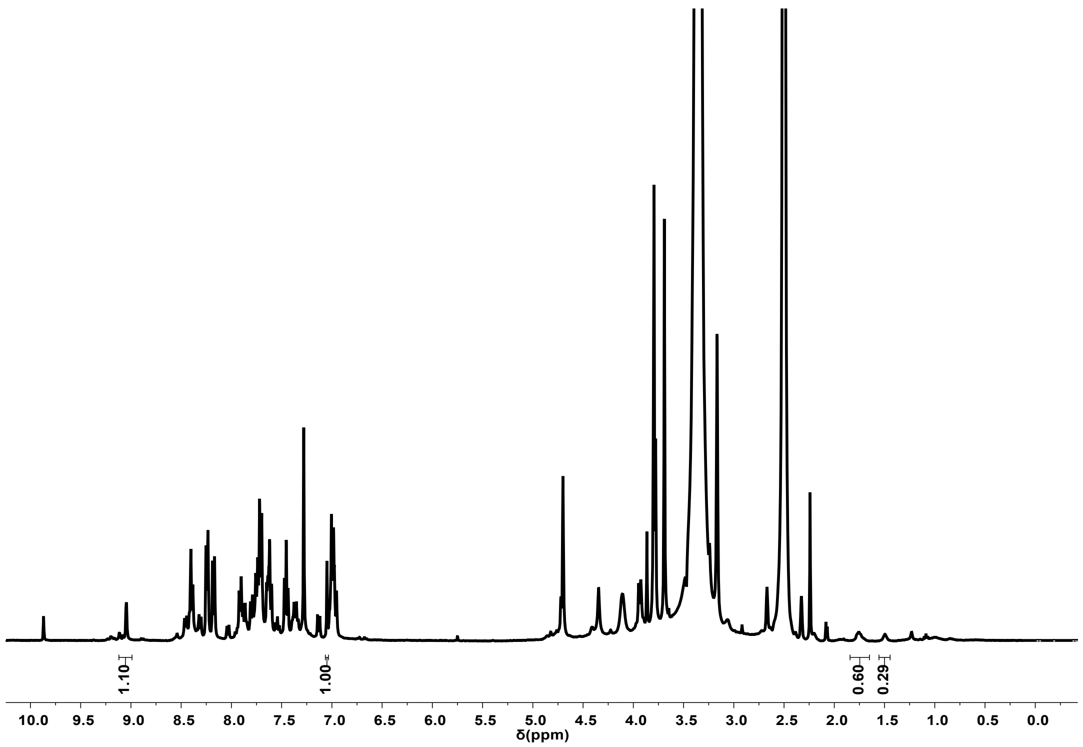

Figure S8 ^{1}H-NMR (DMSO-d_6, 400 MHz) of cage $\mathbf{C_6@2}$ 12 h after the addition of ethylenediamine **3** and *p*-methoxybenzaldehyde **9** to cage $\mathbf{C_6@8}$. (yield=76%, based on internal standard *p*-xylene at 7.1 ppm). 2:1 ratio among encapsulated and free $\mathbf{C_6}$ dicarboxylate.

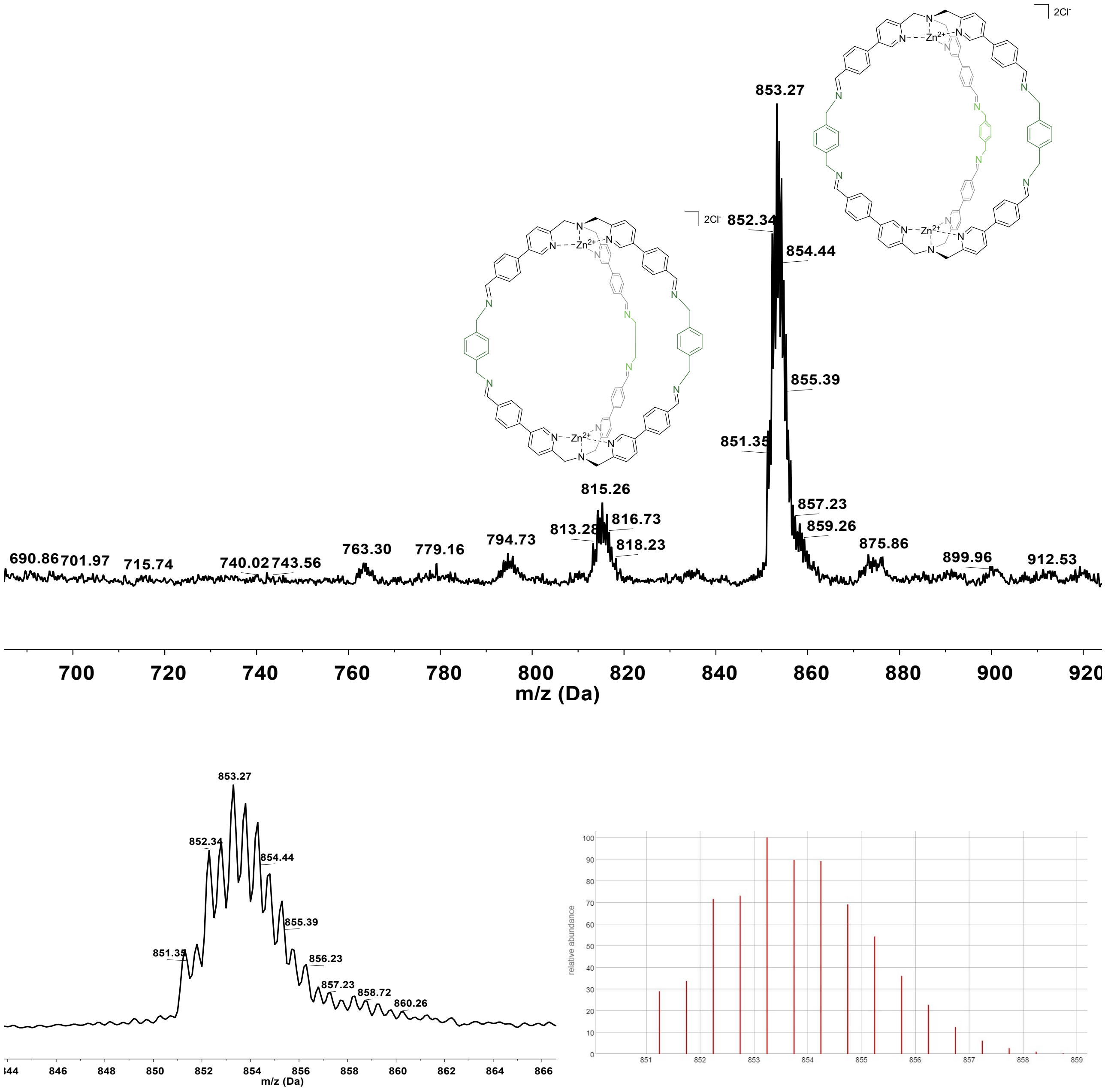

Figure S9 Experimental, and calculated ESI-MS pattern (in CH_3CN/0.1% HCOOH) of the reaction mixture. Due to the low affinity of the guest, only the empty cage is observed.

5 Cage-to-Cage Competing Guests

5.1 Guest substitution from C_{10} to C_6

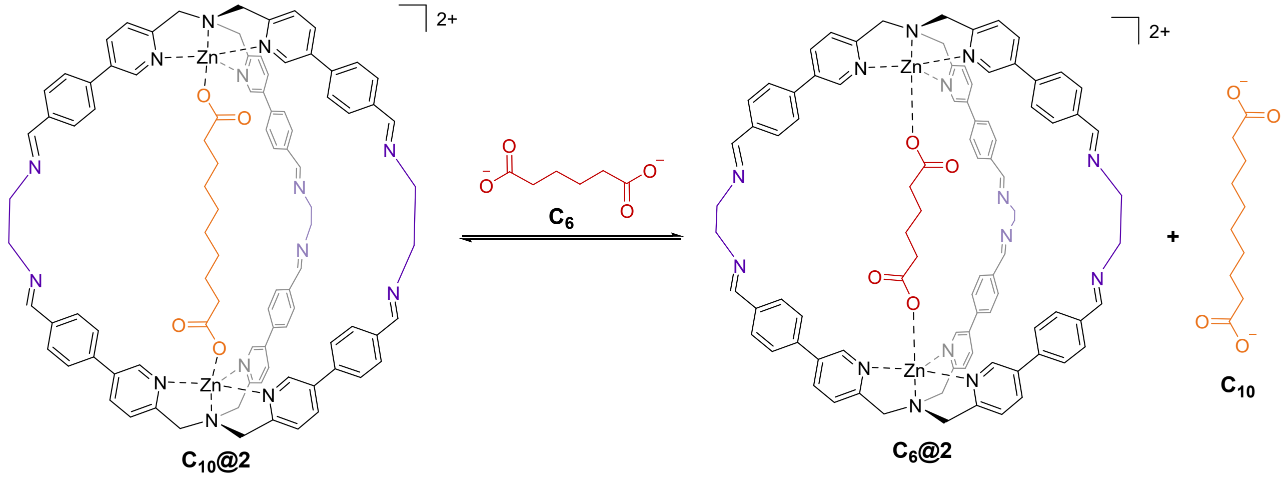

Procedure for the guest displacement. Perchlorate counterions are removed for clarity.

To 350 µL (0.70 µmol) of a solution 0.002 M of complex **1** in DMSO-d_6, 18 µL (0.35 µmol) of a solution 0.02 M in DMSO-d_6 of a dicarboxylate $\mathbf{C_{10}}$, 87 µL (1.75 µmol) of a solution 0.02 M in DMSO-d_6 of ethylenediamine **3**, and 40 µL (0.49 µmol) of a solution 0.012 M in DMSO-d_6 of *p*-xylene were added in a NMR tube and left at room temperature for 12 hours (yield=95% determined *via* ^{1}H-NMR on internal standard *p*-xylene). After 12 hours 36 µL (0.35 µmol) of a solution 0.01 M in DMSO-d_6 of adipate $\mathbf{C_6}$ were added to the NMR tube in 4 aliquots. At the end, the complete exchange of the guest from $\mathbf{C_{10}}$ to $\mathbf{C_6}$ occurred, obtaining the selective uptake and release of the guests.

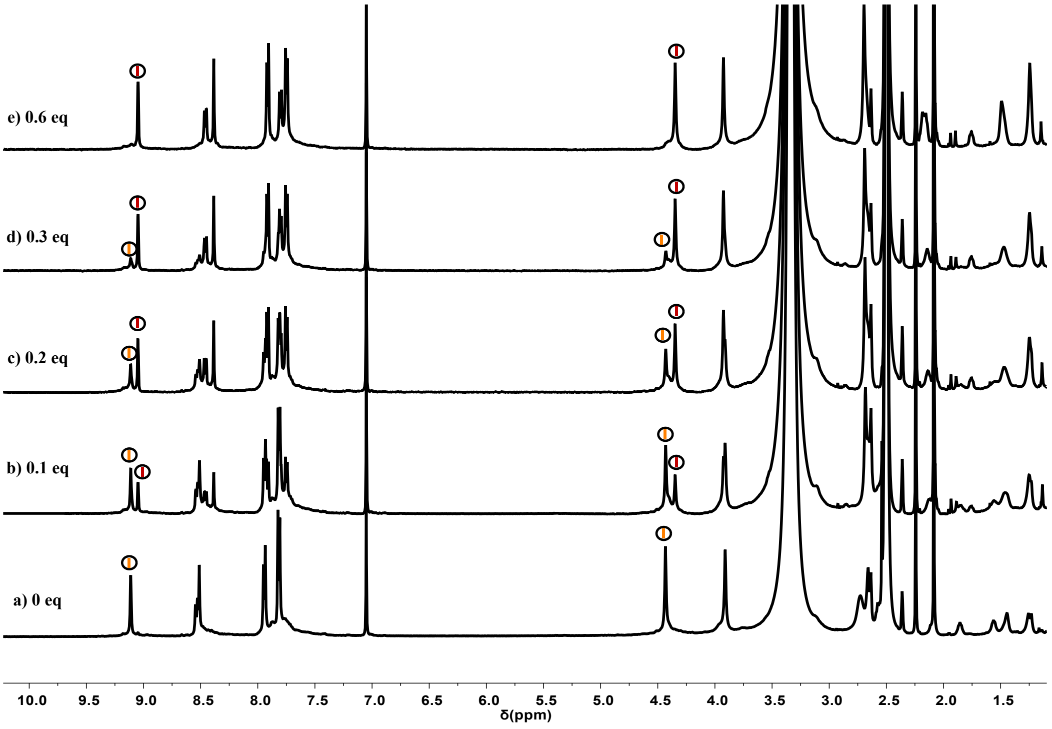

Figure S10 ^{1}H-NMR (DMSO-d_6, 500 MHz) titration experiment of the preformed cage $\mathbf{C_{10}@2}$ using adipic acid $\mathbf{C_6}$ as competitor. The black circle indicates the cage, orange stick indicates $\mathbf{C_{10}}$ while the red stick indicates $\mathbf{C_6}$ (internal standard *p*-xylene at 7.1 ppm).

5.2 Cage to Cage conversion with selective release and uptake of the guests

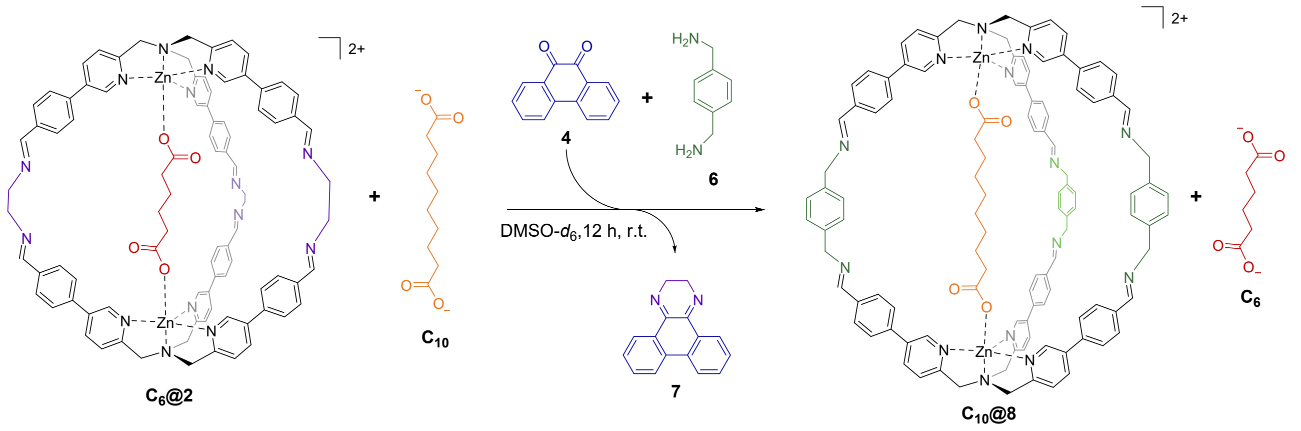

Procedure for the cage to cage transformation. Perchlorate counterions are removed for clarity.

To the prepared mixture in which $\mathbf{C_{10}}$ was substituted with $\mathbf{C_6}$ as guest in cage **2**, 87 µL (1.75 µmol) of a solution 0.02 M in DMSO-d_6 of *p*-xylylenediamine **6** and 44 µL (1.75 µmol) of a solution 0.04 M in DMSO-d_6 of 1,3-phenantrenequinone **4** were added to the NMR tube and the mixture was followed by NMR for 12 hours at room (yield 95% determined *via* ^{1}H-NMR on internal standard *p*-xylene). At the end of the reaction, the complete cage conversion from $\mathbf{C_6@2}$ to $\mathbf{C_{10}@8}$ occurred with the selective uptake and exchange of the guest from $\mathbf{C_6}$ to $\mathbf{C_{10}}$.

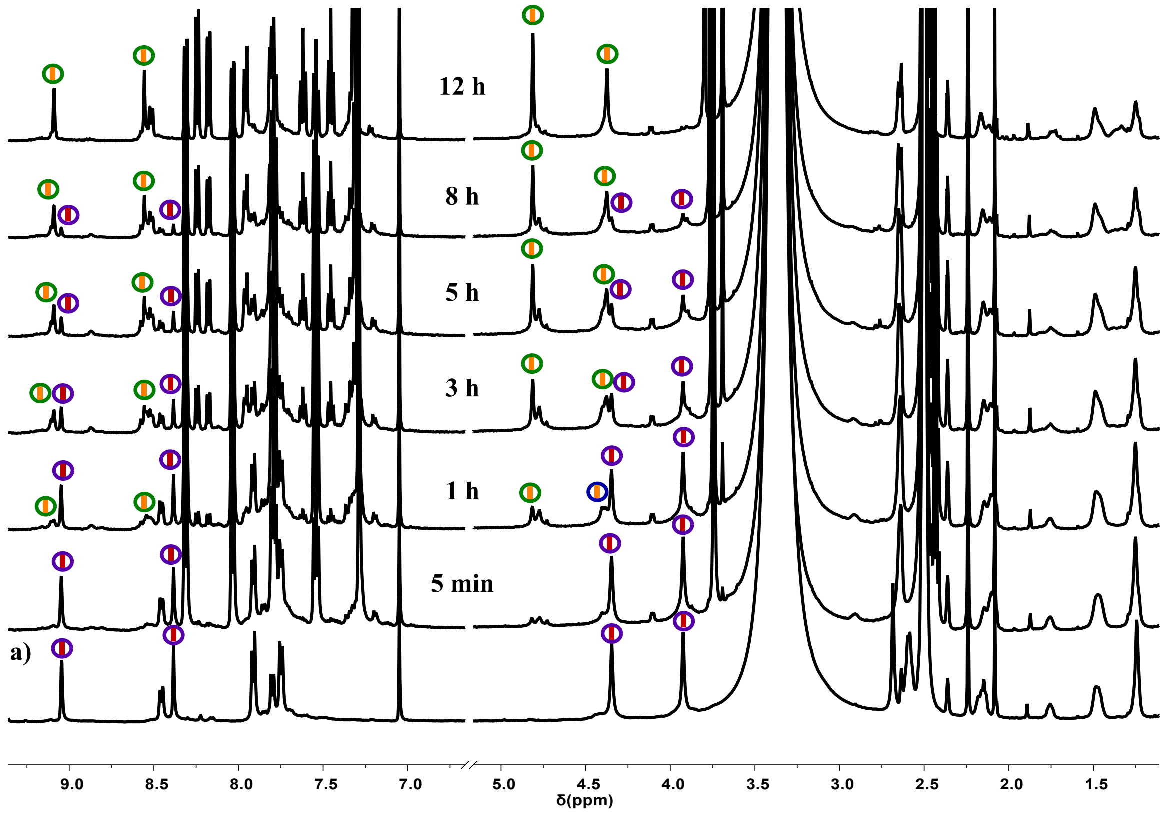

Figure S11 ^{1}H-NMR (DMSO-d_6, 500 MHz) spectra in time progression of the cage $\mathbf{C_6@2}$ after the addition of 2.5 equiv. of **6** and 2.5 equiv. of **4**. Times are related to the addition of **4** and **6**. a)Spectrum of $\mathbf{C_6@2}$ before the addition of the subcomponents. The green circle with the orange stick indicates $\mathbf{C_{10}@8}$ while the purple circle with the red stick indicates $\mathbf{C_6@2}$ (internal standard *p*-xylene at 7.1 ppm).

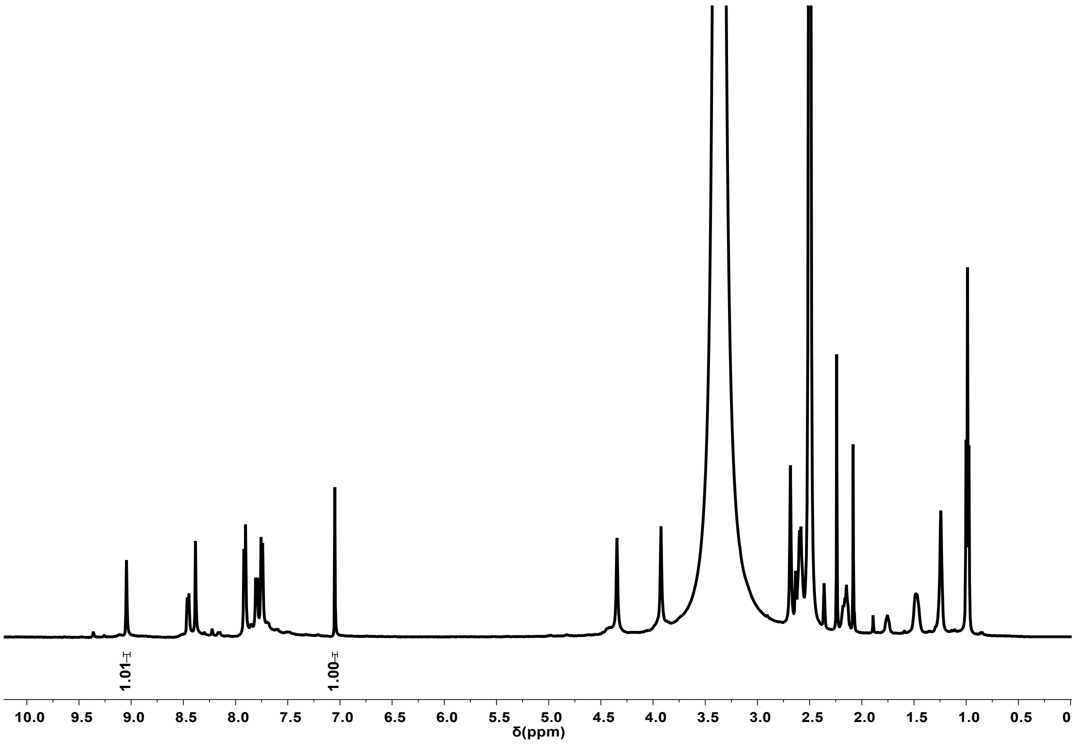

Figure S12 ^{1}H-NMR (DMSO-d_6, 500 MHz) of cage $\mathbf{C_6@2}$ in the presence of $\mathbf{C_{10}}$ (yield=95%, based on internal standard *p*-xylene at 7.1 ppm).

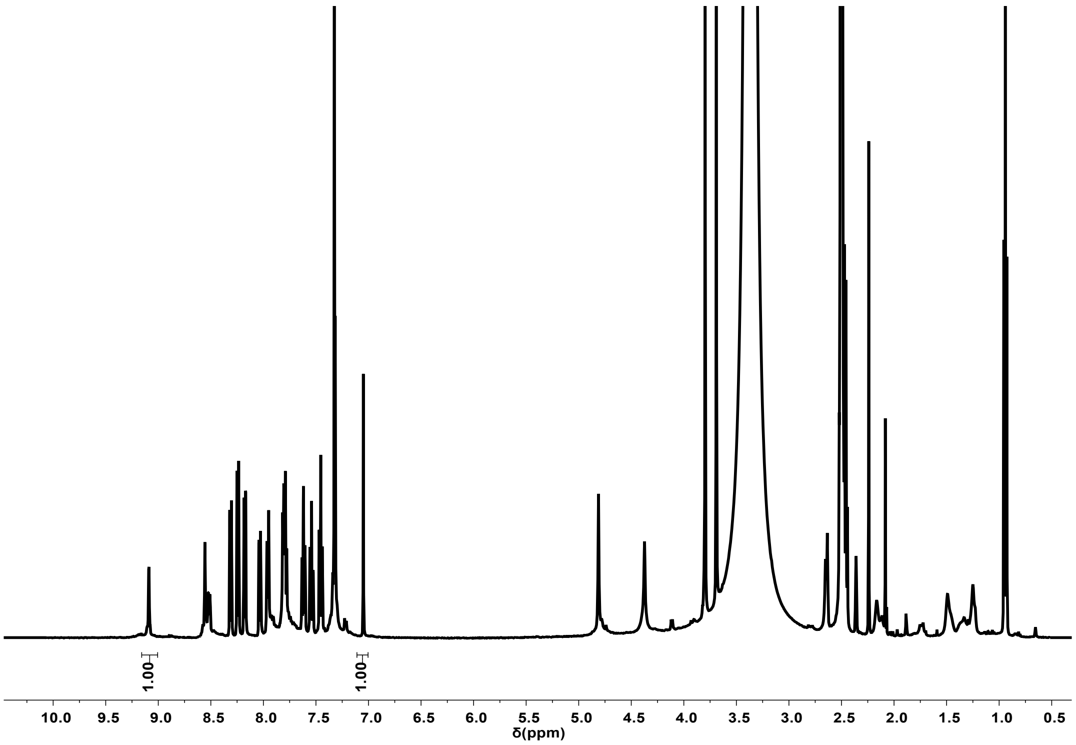

Figure S13 ^{1}H-NMR (DMSO-d_6, 500 MHz) of cage $\mathbf{C_{10}@8}$ formed after the addition of 2.5 equiv. of **6** and 2.5 equiv. of **4** in the presence of $\mathbf{C_{10}}$ to cage $\mathbf{C_6@2}$ (yield=94%, based on internal standard *p*-xylene at 7.1 ppm).

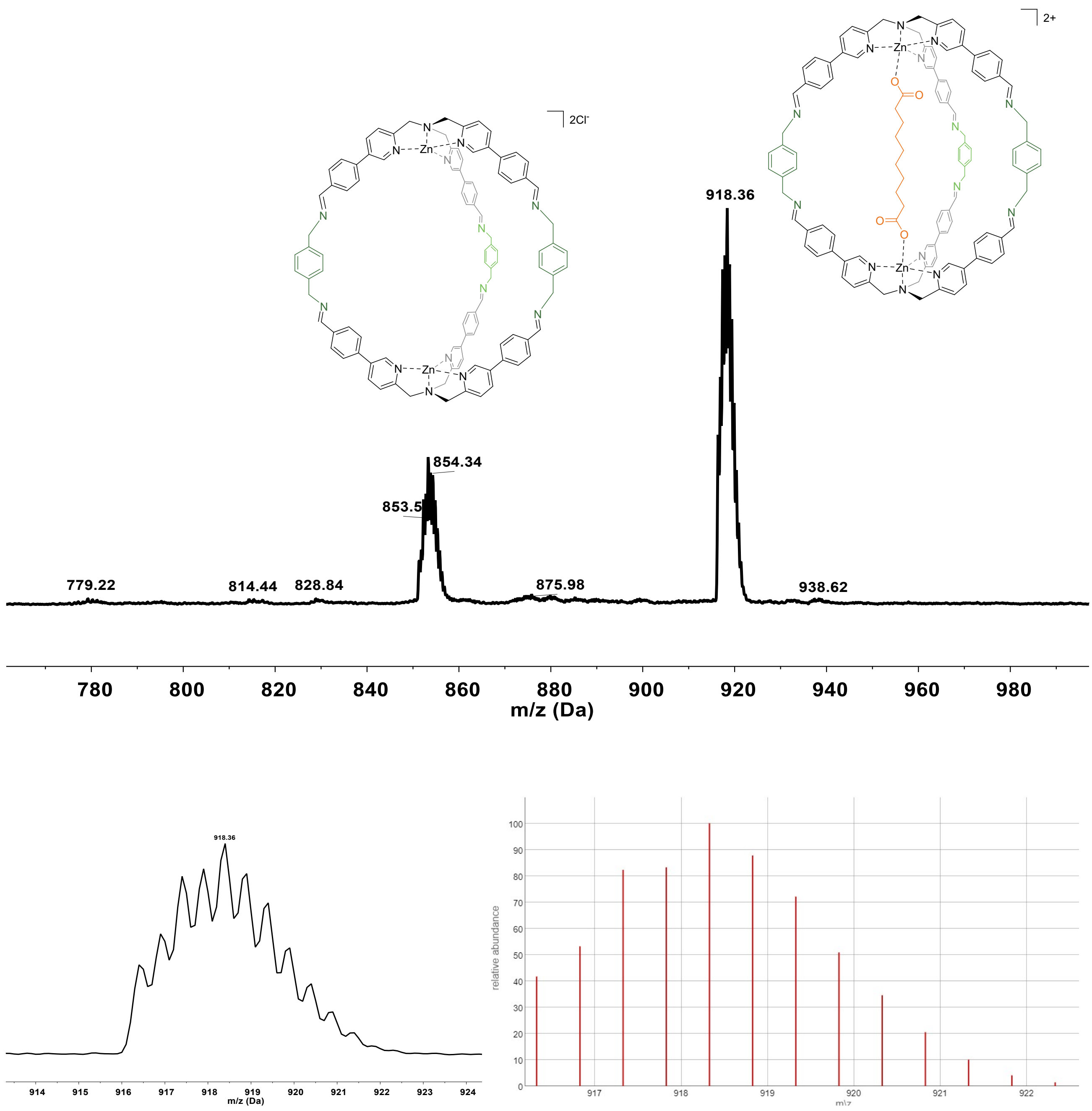

Figure S14 Experimental, and calculated ESI-MS pattern (in CH_3CN/0.1% HCOOH) of the reaction mixture after the cage to cage transformation. Due to dilution for the analysis, the empty cage was also observed.

6 Chiral cage Assembly-Disassembly-Assembly with enantiomers

6.1 Selective encapsulation using a racemic mixture

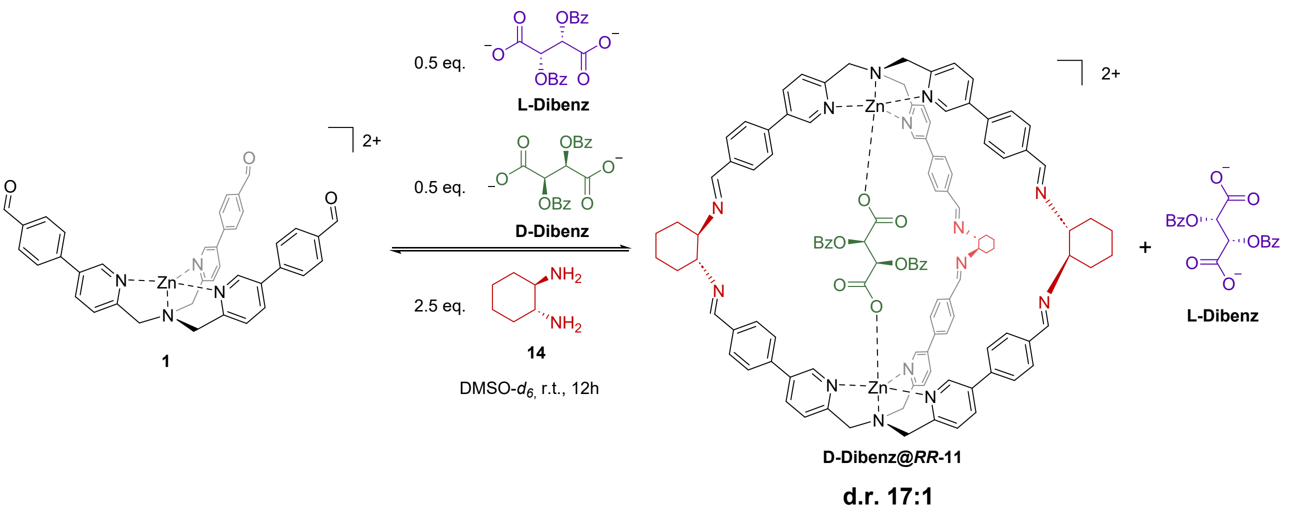

Procedure for the selective encapsulation. Perchlorate counterions are removed for clarity.

To 350 µL (0.70 µmol) of a solution 0.002 M of complex **1** in DMSO-d_6, 18 µL (0.35 µmol) of a solution 0.02 M in DMSO-d_6 of a dicarboxylic acid **D-Dibenz**, 18 µL (0.35 µmol) of a solution 0.02 M in DMSO-d_6 of a dicarboxylate **L-Dibenz**, 87 µL (1.75 µmol) of a solution 0.02 M in DMSO-d_6 of (1*R*,2*R*)-(-)-1,2-diaminocyclohexane **14**, and 35 µL (0.70 µmol) of a solution 0.02 M in DMSO-d_6 of 1,3,5-trimethoxybenzene were added in a NMR tube and left at room temperature for 12 hours (yield=94% determined *via* ^{1}H-NMR on internal standard 1,3,5-trimethoxybenzene). From the NMR, a preference for the encapsulation of **D-Dibenz** was observed, with a diastereomeric ratio 17:1.

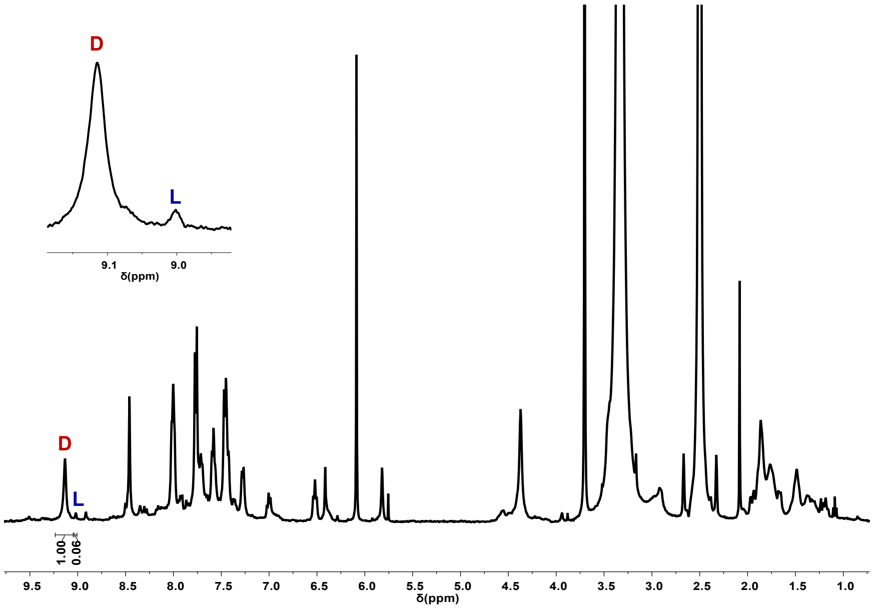

Figure S15 ^{1}H-NMR (DMSO-d_6, 400 MHz) of cage **5** in the presence of a racemic mixture of Benzoyl-tartrate **Dibenz**. From integration of the α -pyridine protons peak, a preference for the encapsulation of **D-Dibenz** was observed, with a diastereomeric ratio 17:1.

6.2 Cage-to-Cage Enantiomeric Competing Guests

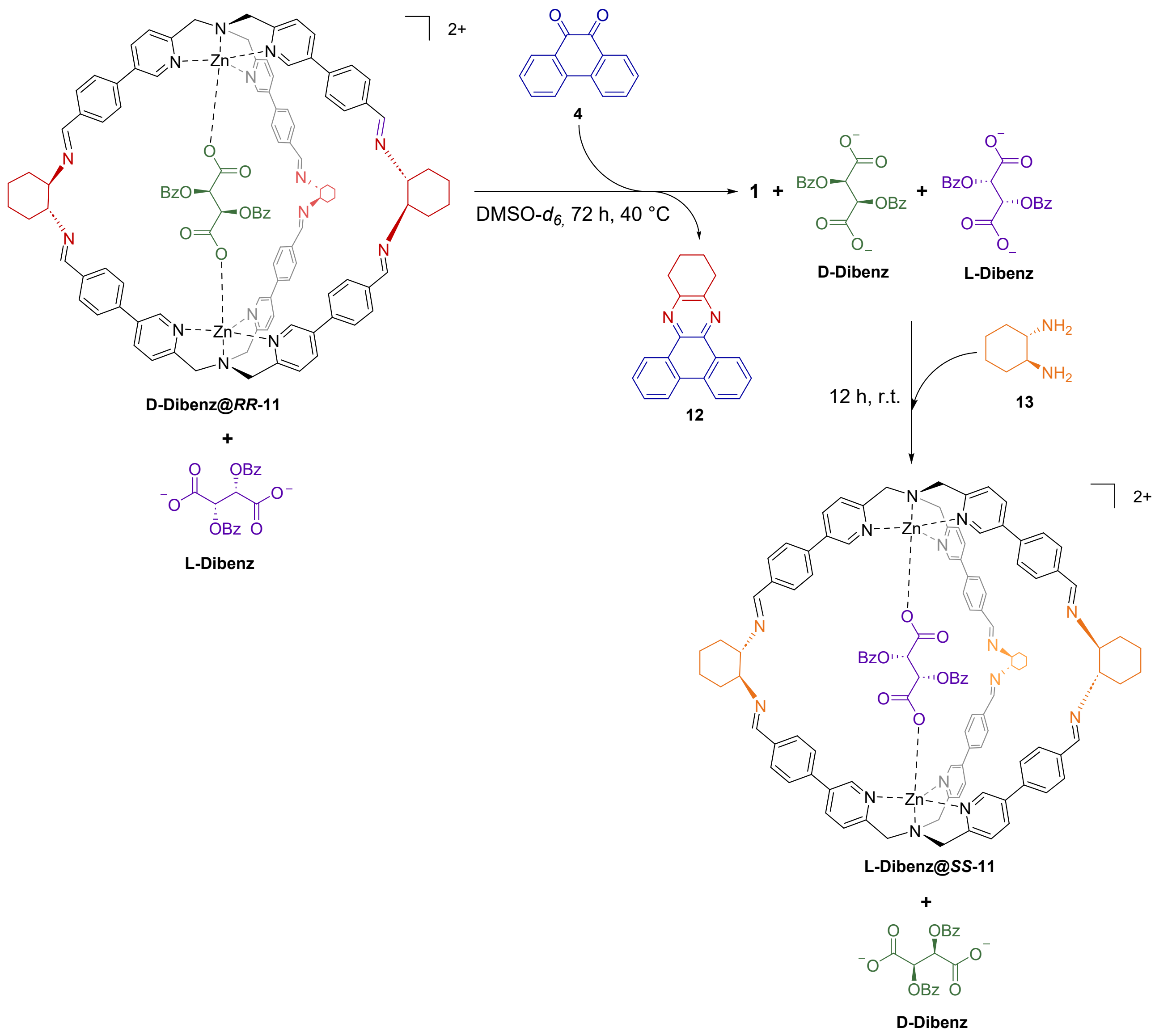

Procedure for the cage to cage transformation. Perchlorate counterions are removed for clarity.

a) Disassembly of cage **D-Dibenz@*RR*-11** with **4**:

After 12 hours, 44 µL (1.75 µmol) of a solution 0.04 M in DMSO-d_6 of 1,3-phenantrenequinone **4** were added to the previously prepared mixture in the NMR tube, and the solution was left for 72 hours at 40 °C. ^{1}H-NMR showed the complete disassembly of the cage as confirmed by the disappearance of the imine peak at 8.5 ppm and the formation of the aldehyde peak of complex **1** at 10 ppm.

b) Assembly of cage **L-Dibenz@*SS*-11** with **13**:

After 72 hours, 87 µL (1.75 µmol) of a solution 0.02 M in DMSO-d_6 of (1*S*,2*S*)-(+)-1,2-diaminocyclohexane **13** were added to the NMR tube and the mixture was left for 12 hours at room temperature. ^{1}H-NMR confirmed the formation of cage **L-Dibenz@*SS*-11** (yield 60% determined *via* ^{1}H-NMR on internal standard 1,3,5-trimethoxybenzene).

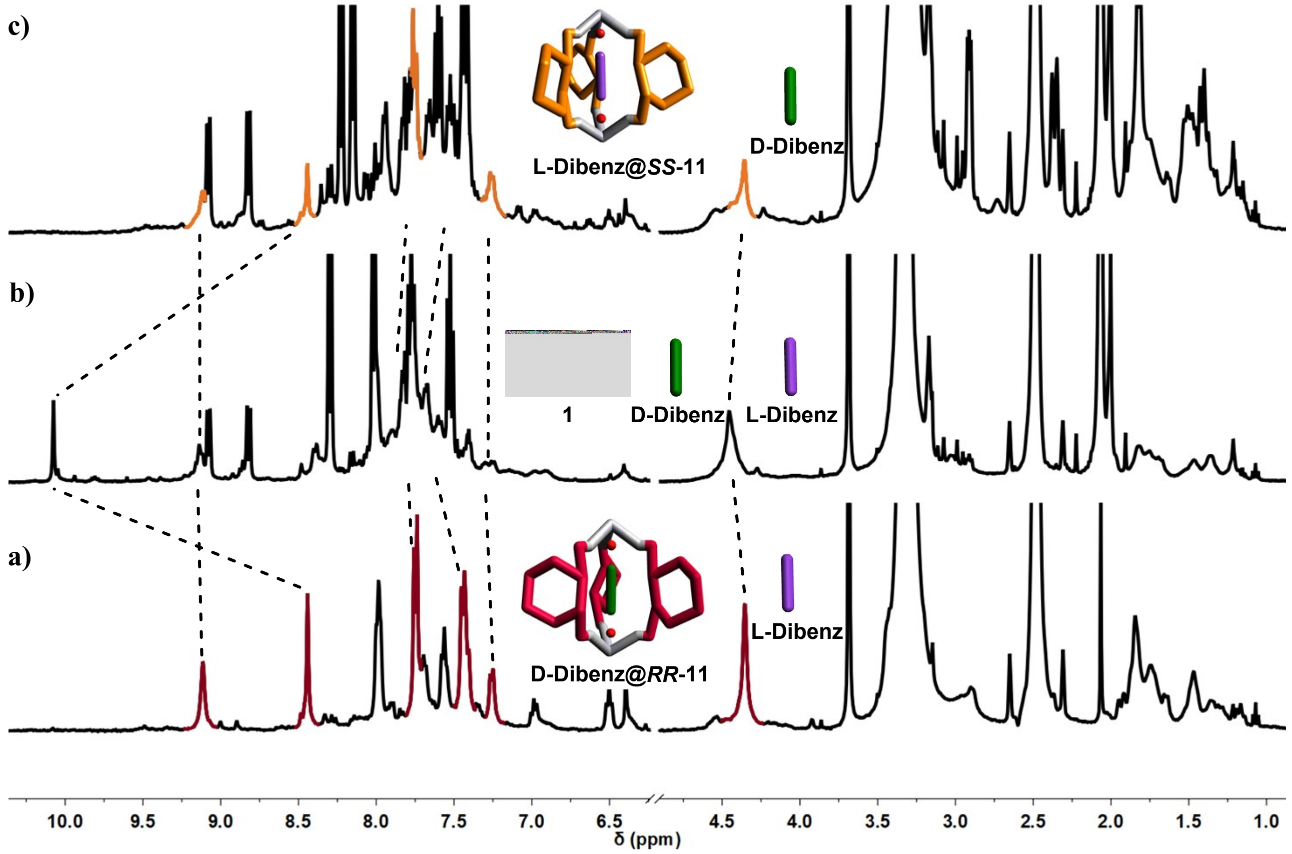

Figure S16 ^{1}H-NMR (DMSO-d_6, 400 MHz) of a) cage **D-Dibenz@*RR*-11**, b) 72 h after the addition of **4**, and c) 12 h after the addition of *S*,*S*- diaminocyclohexane **13** that led to the formation of cage **L-Dibenz@*SS*-11**.

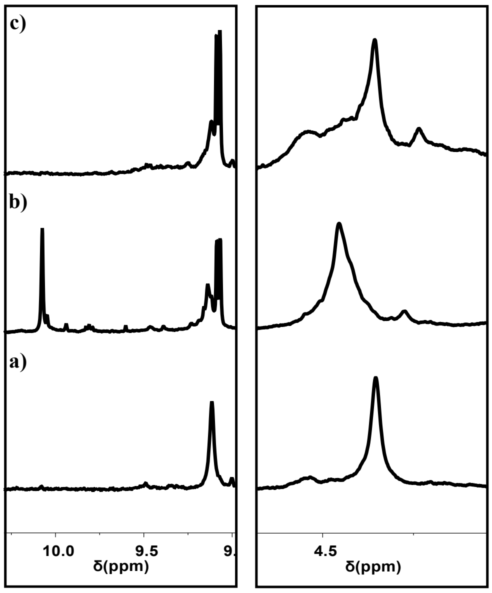

Figure S17 Partial ^{1}H-NMR (DMSO-d_6, 400 MHz) of the α -pyridine protons peak. (left) and the CH_2 benzylic protons of the **TPMA** scaffold (right). a) cage **D-Dibenz@*RR*-11**, b) 72 h after the addition of **4**, and c) 12 h after the addition of *S*,*S*- diaminocyclohexane **13** that led to the formation of cage **L-Dibenz@*SS*-11**.

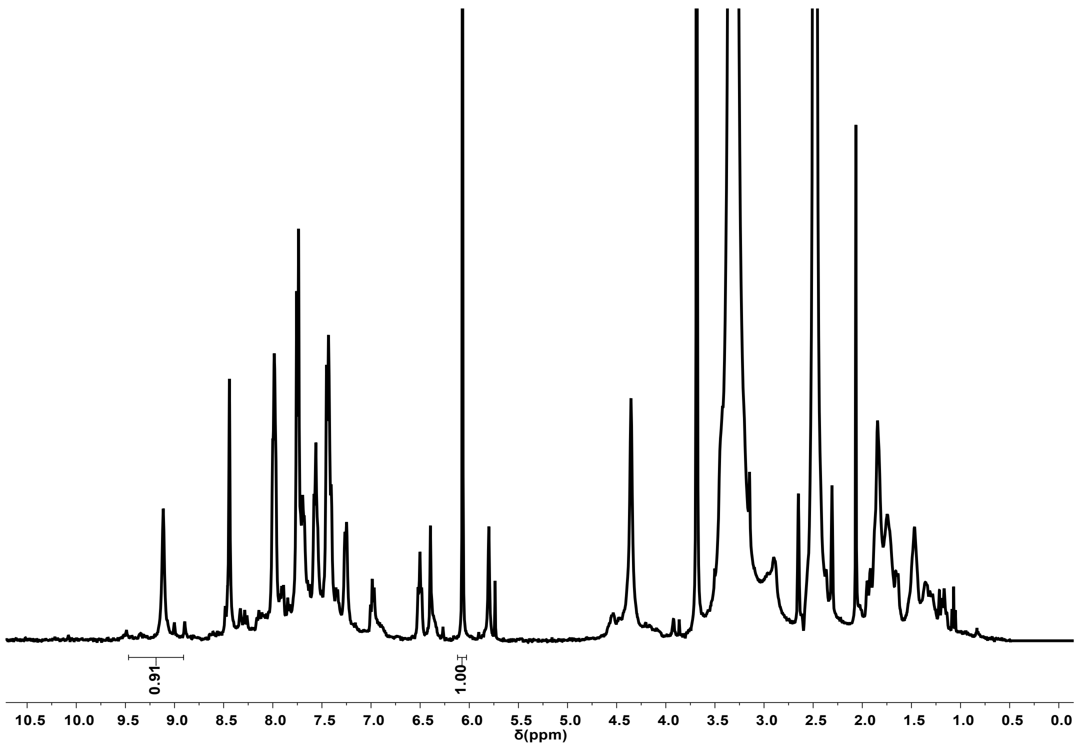

Figure S18 ^{1}H-NMR (DMSO-d_6, 400 MHz) of cage **7** in the presence of a racemic mixture of Benzoyl-tartrate **Dibenz** (yield=94 % determined *via* ^{1}H-NMR on internal standard 1,3,5-trimethoxybenzene at 6.1 ppm).

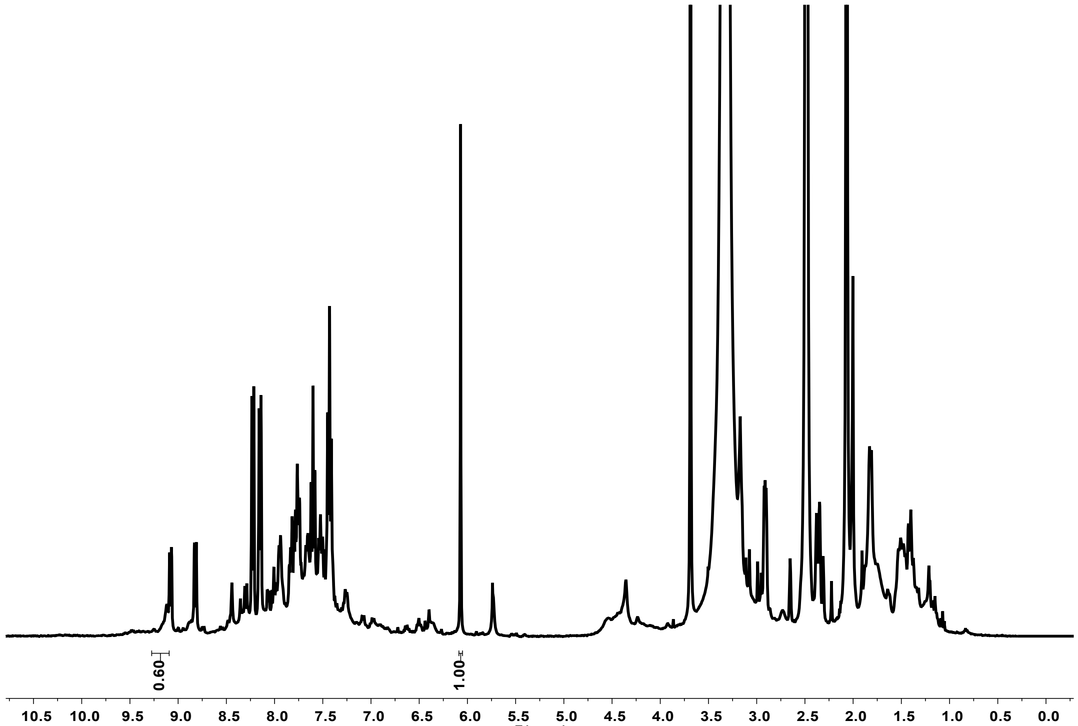

Figure S19 ^{1}H-NMR (DMSO-d_6, 400 MHz) of cage **L-Dibenz@*SS*-11** after the disassembly-assembly cycle (yield=60% determined *via* ^{1}H-NMR on internal standard 1,3,5-trimethoxybenzene at 6.1 ppm).

7 NMR and ESI characterizations

7.1 $C_6@2$

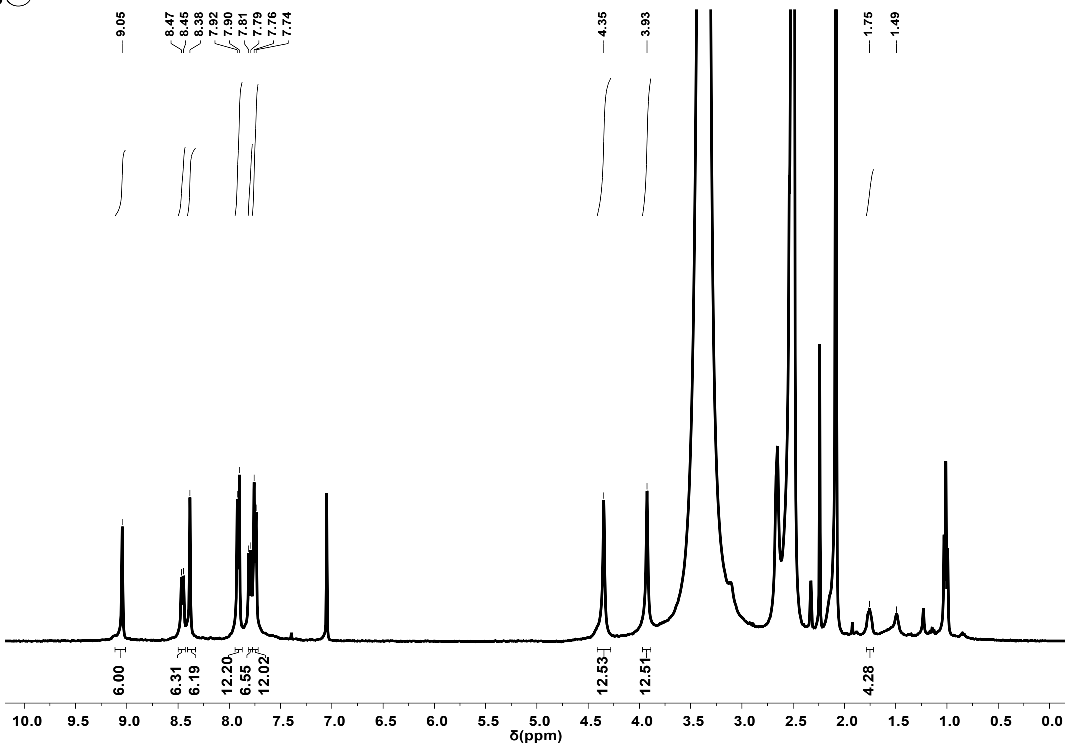

Figure S20 ^{1}H-NMR spectrum (400 MHz, 301 K, DMSO-d_6) of cage $\mathbf{C_6@2}$ (internal standard *p*-xylene at 7.1 ppm).

7.2 $C_{10}@2$

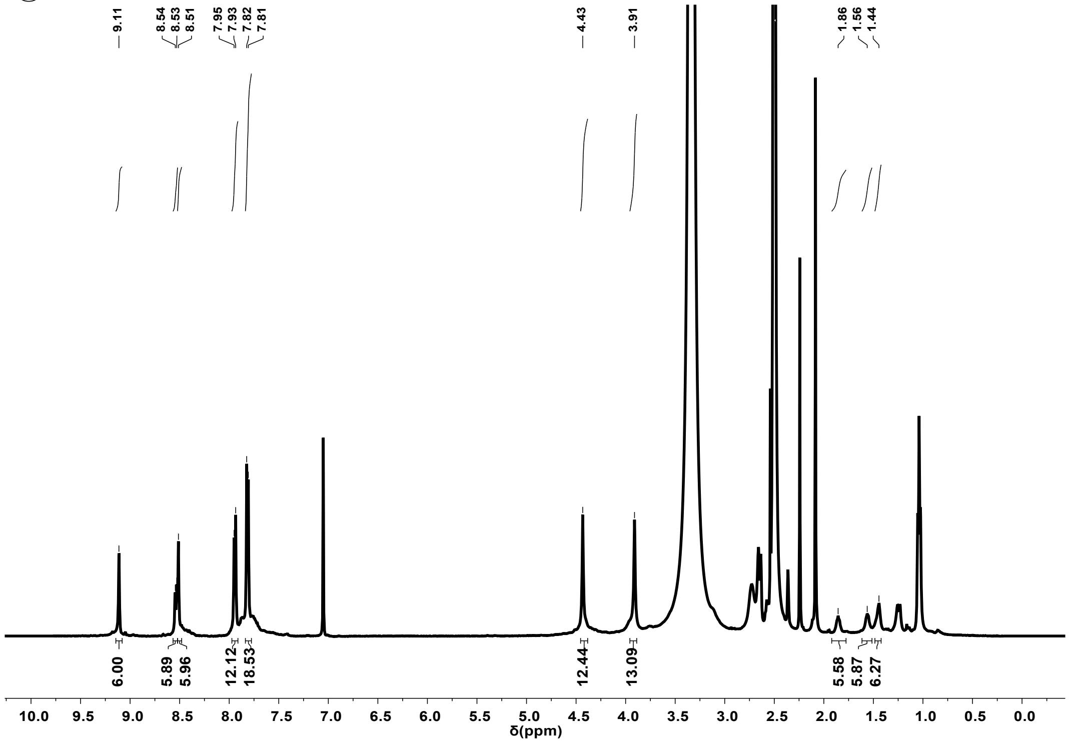

Figure S21 ^{1}H-NMR spectrum (500 MHz, 301 K, DMSO-d_6) of cage $\mathbf{C_{10}@2}$ (internal standard *p*-xylene at 7.1 ppm).

7.3 C_6@8

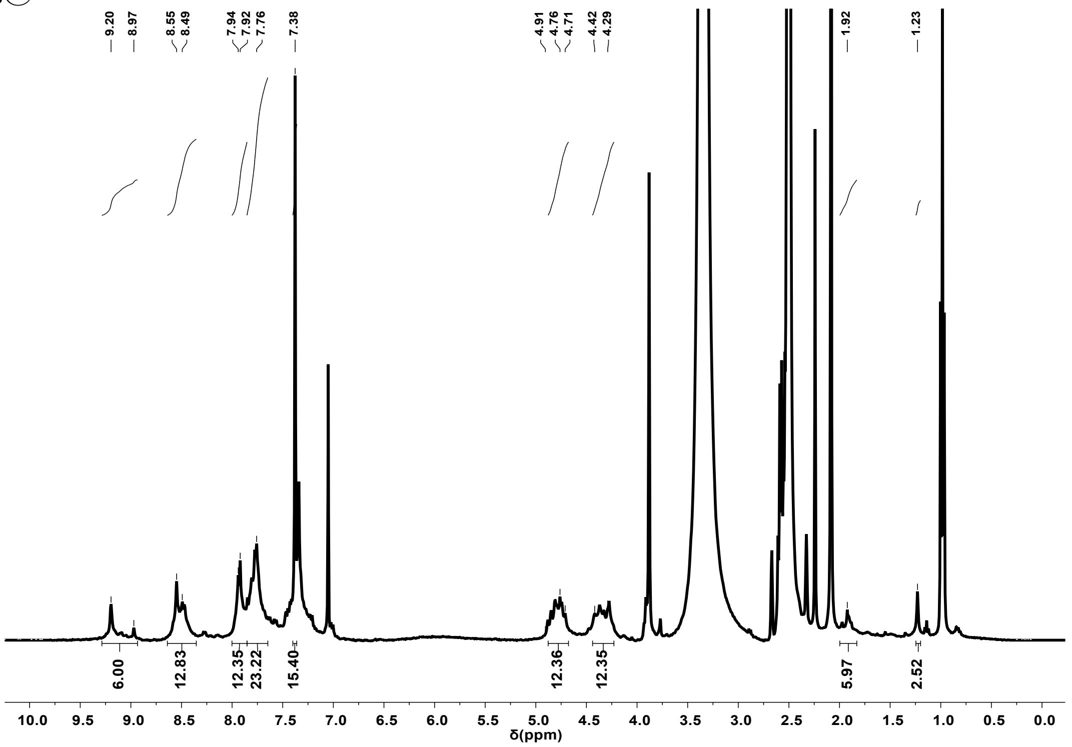

Figure S22 ^{1}H-NMR spectrum (400 MHz, 301 K, DMSO-d_6) of cage **C_6@8** (internal standard *p*-xylene at 7.1 ppm).

7.4 C_{10}@8

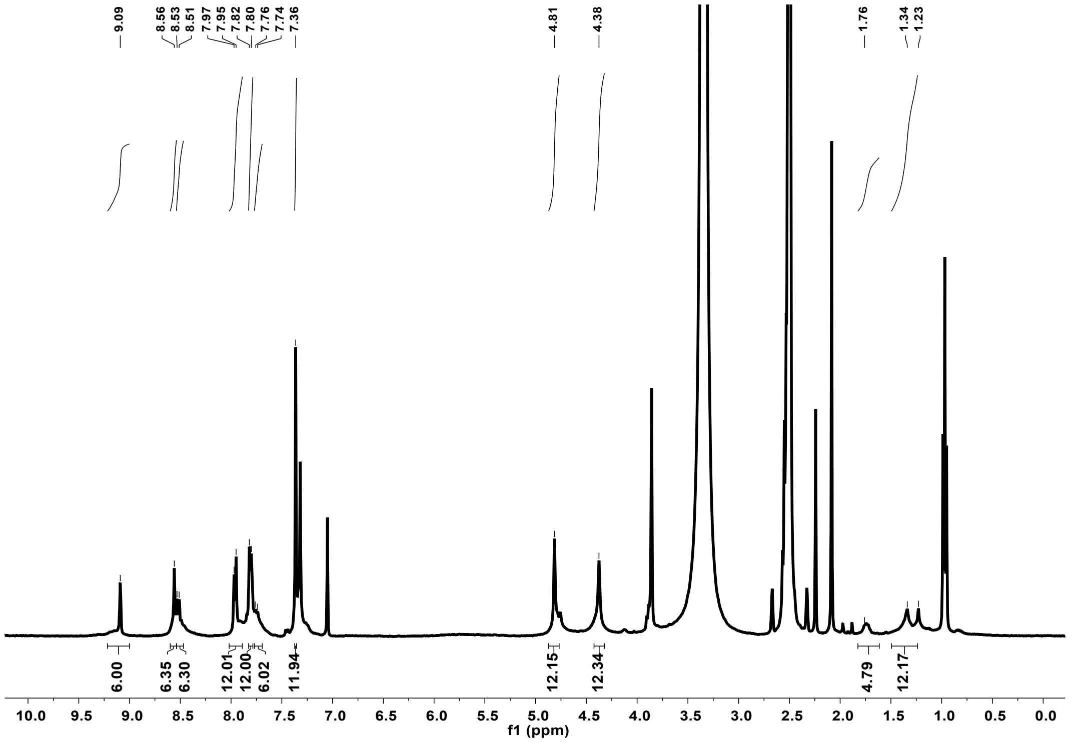

Figure S23 ^{1}H-NMR spectrum (400 MHz, 301 K, DMSO-d_6) of cage **C_{10}@8** (internal standard *p*-xylene at 7.1 ppm).

7.5 L-Dibenz@*RR*-11

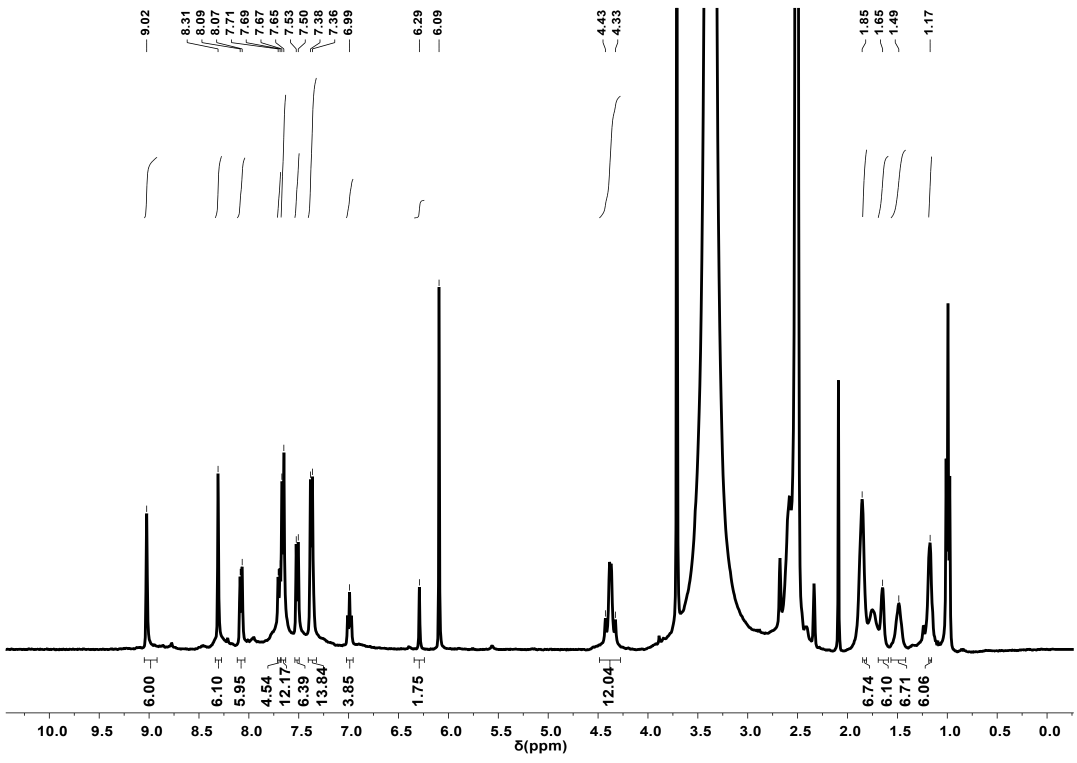

Figure S24 ^{1}H-NMR spectrum (400 MHz, 301 K, DMSO-d_6) of cage **L-Dibenz@*RR*-11** (internal standard 1,3,5-trimethoxybenzene at 6.1 ppm).

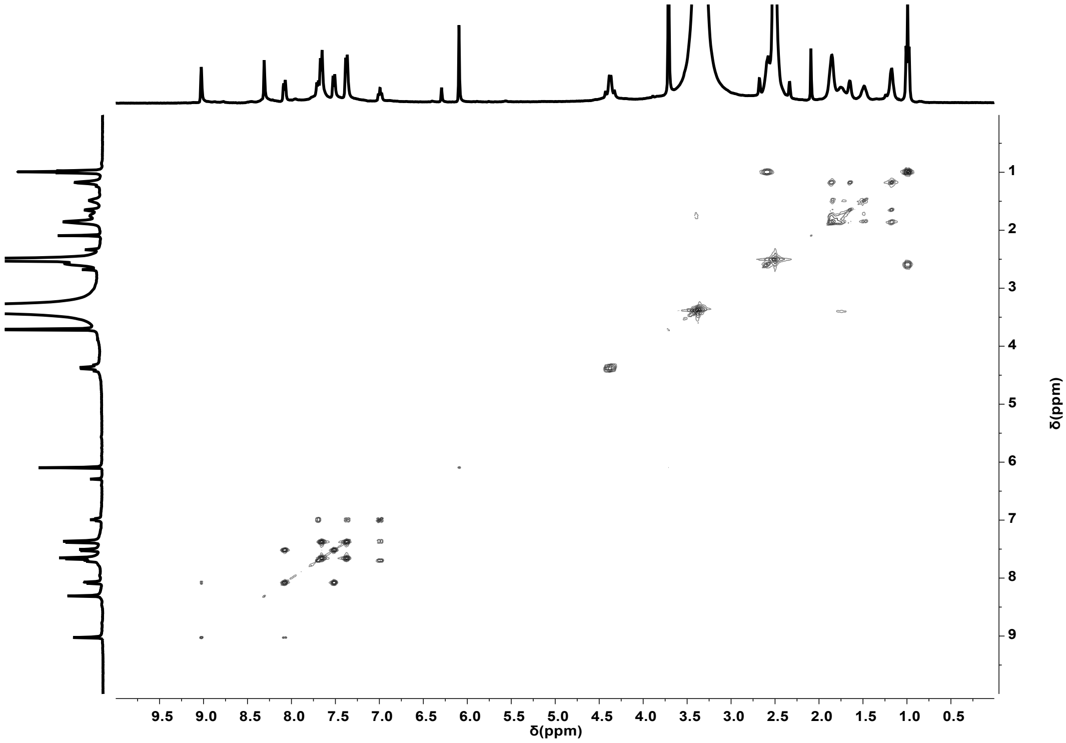

Figure S25 ^{1}H-^{1}H COSY spectrum (500 MHz, 301 K, DMSO-d_6) of cage **L-Dibenz@*RR*-11**.

7.6 D-Dibenz@*RR*-11

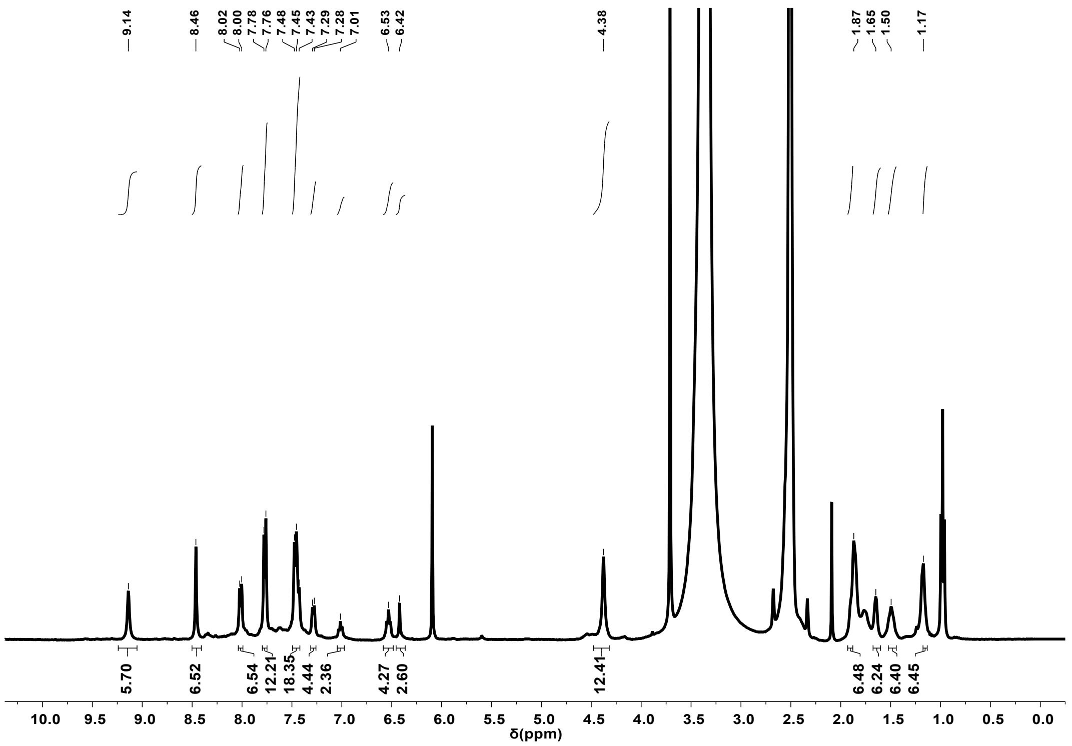

Figure S26 ^{1}H-NMR spectrum (400 MHz, 301 K, DMSO-d_6) of cage **D-Dibenz@*RR*-11** (internal standard 1,3,5-trimethoxybenzene at 6.1 ppm)..

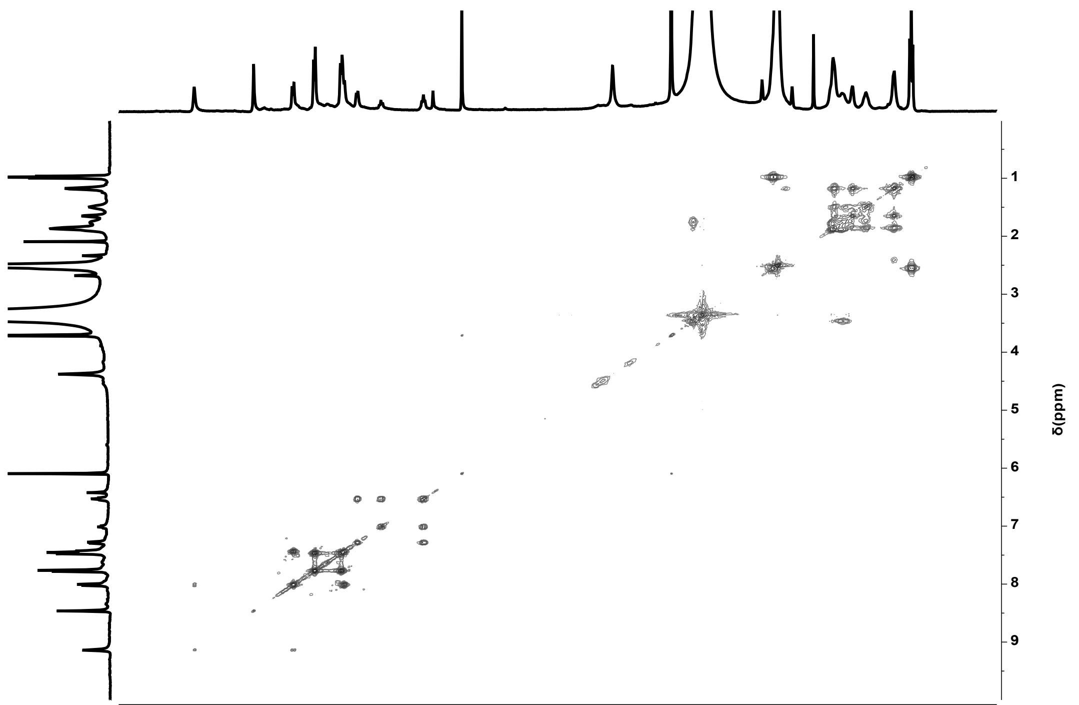

Figure S27 ^{1}H-^{1}H COSY spectrum (500 MHz, 301 K, DMSO-d_6) of cage **D-Dibenz@*RR*-11**.

7.7 L-Dibenz@*SS*-11

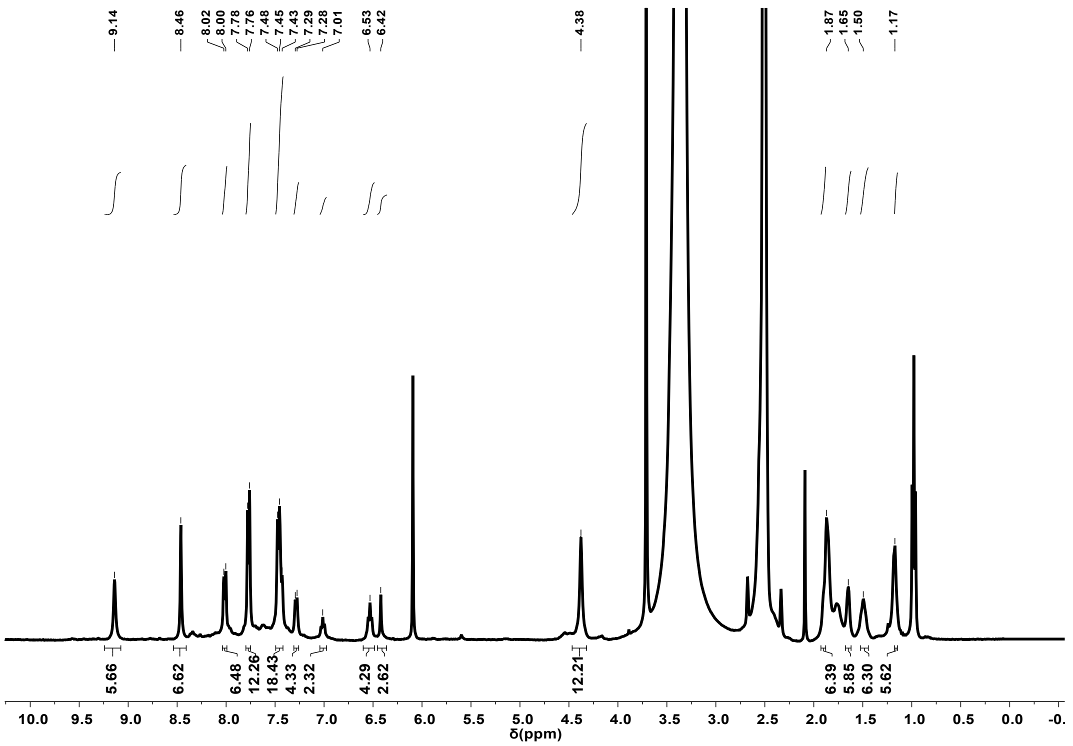

Figure S28 ^{1}H-NMR spectrum (400 MHz, 301 K, DMSO-d_6) of cage **L-Dibenz@*SS*-11** (internal standard 1,3,5-trimethoxybenzene at 6.1 ppm)..

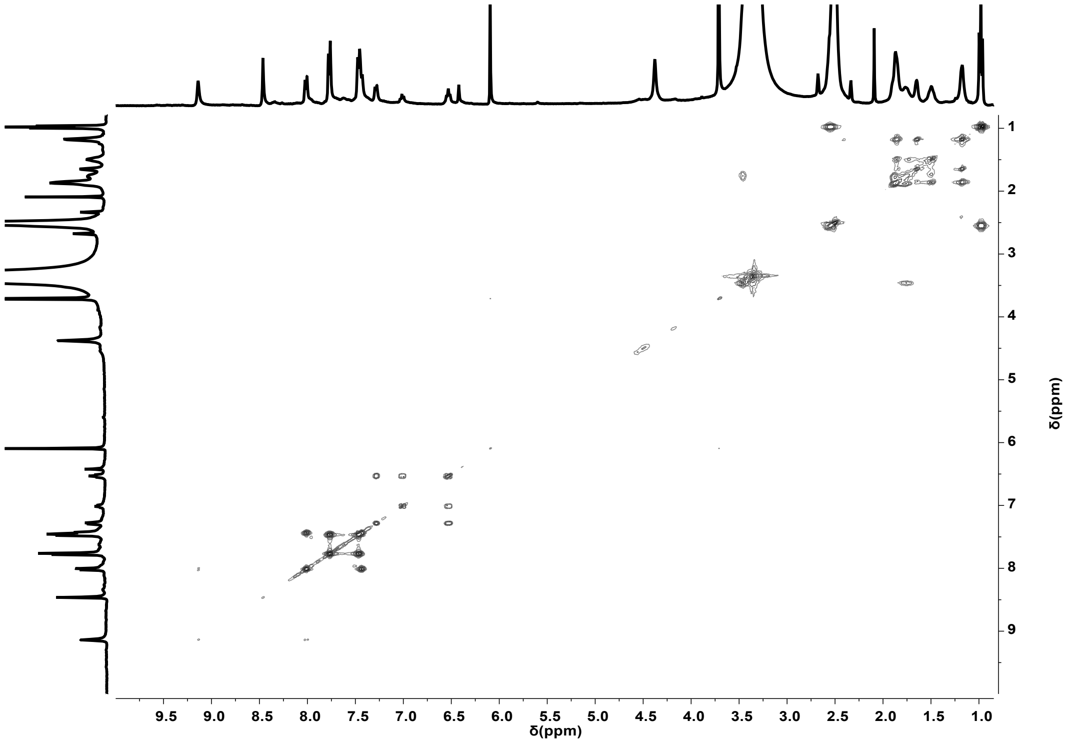

Figure S29 ^{1}H-^{1}H COSY spectrum (500 MHz, 301 K, DMSO-d_6) of cage **L-Dibenz@*SS*-11**.

7.8 D-Dibenz@*SS*-11

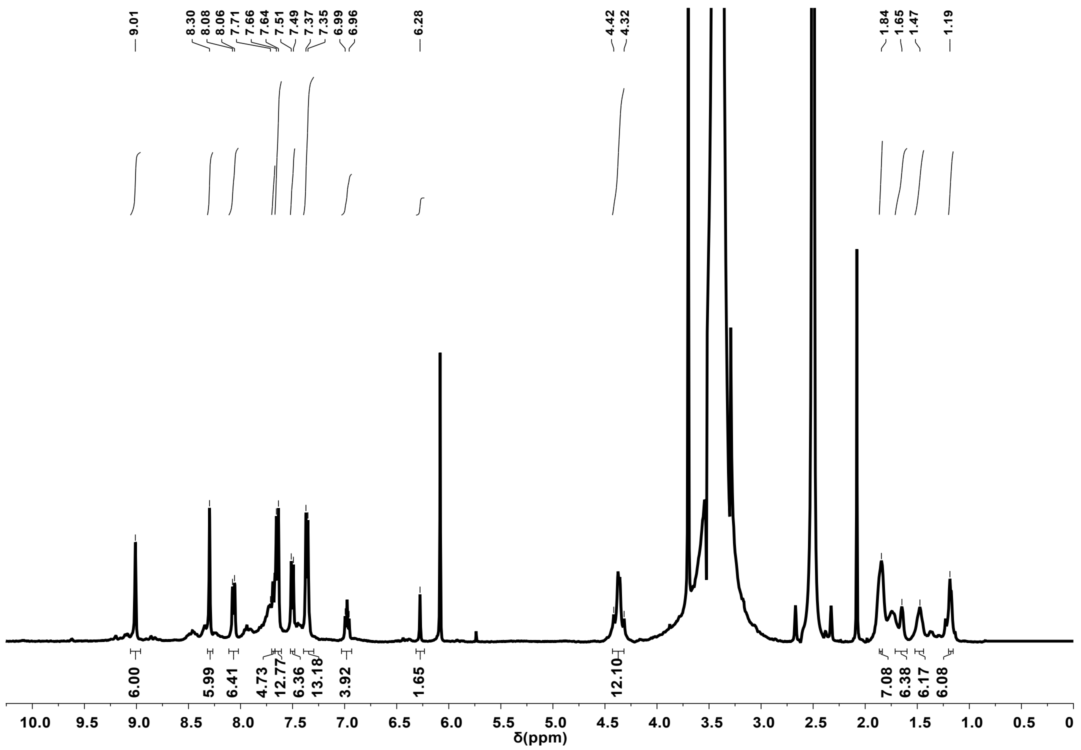

Figure S30^{1}H-NMR spectrum (400 MHz, 301 K, DMSO-d_6) of cage **D-Dibenz@*SS*-11** (internal standard 1,3,5-trimethoxybenzene at 6.1 ppm)..

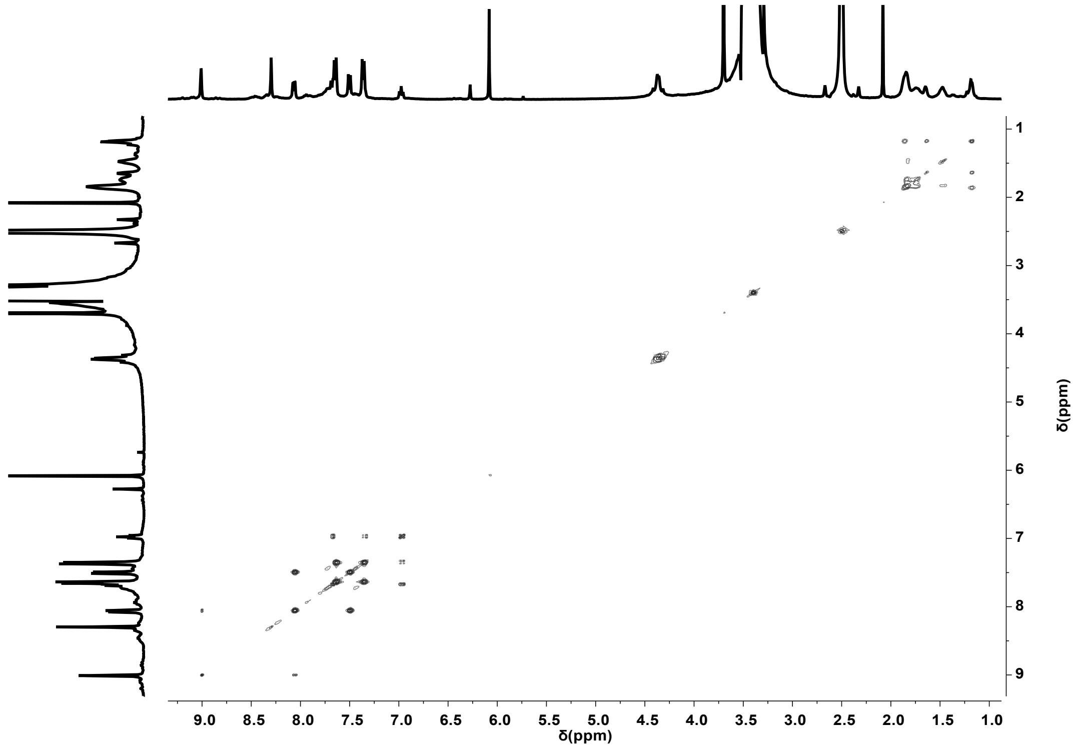

Figure S31 ^{1}H-^{1}H COSY spectrum (500 MHz, 301 K, DMSO-d_6) of cage **D-Dibenz@*SS*-11**.

7.9 ESI-MS spectrum of cage D-Dibenz@*RR*-11

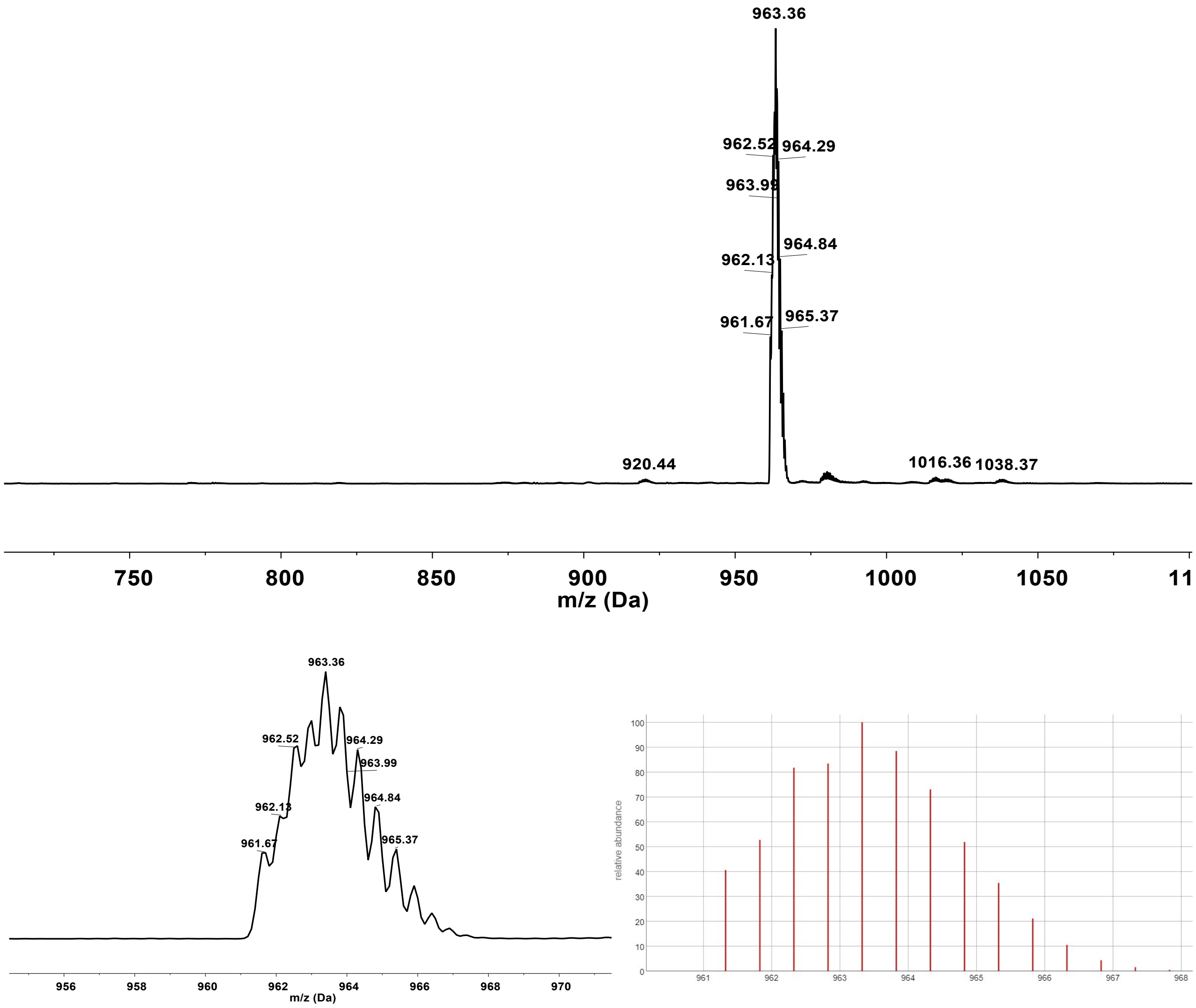

Figure S32 Experimental, and calculated ESI-MS pattern (in CH_3CN/0.1% HCOOH) of cage **D-Dibenz@*RR*-11.**

7.10 ESI-MS spectrum of cage L-Dibenz@*SS*-11

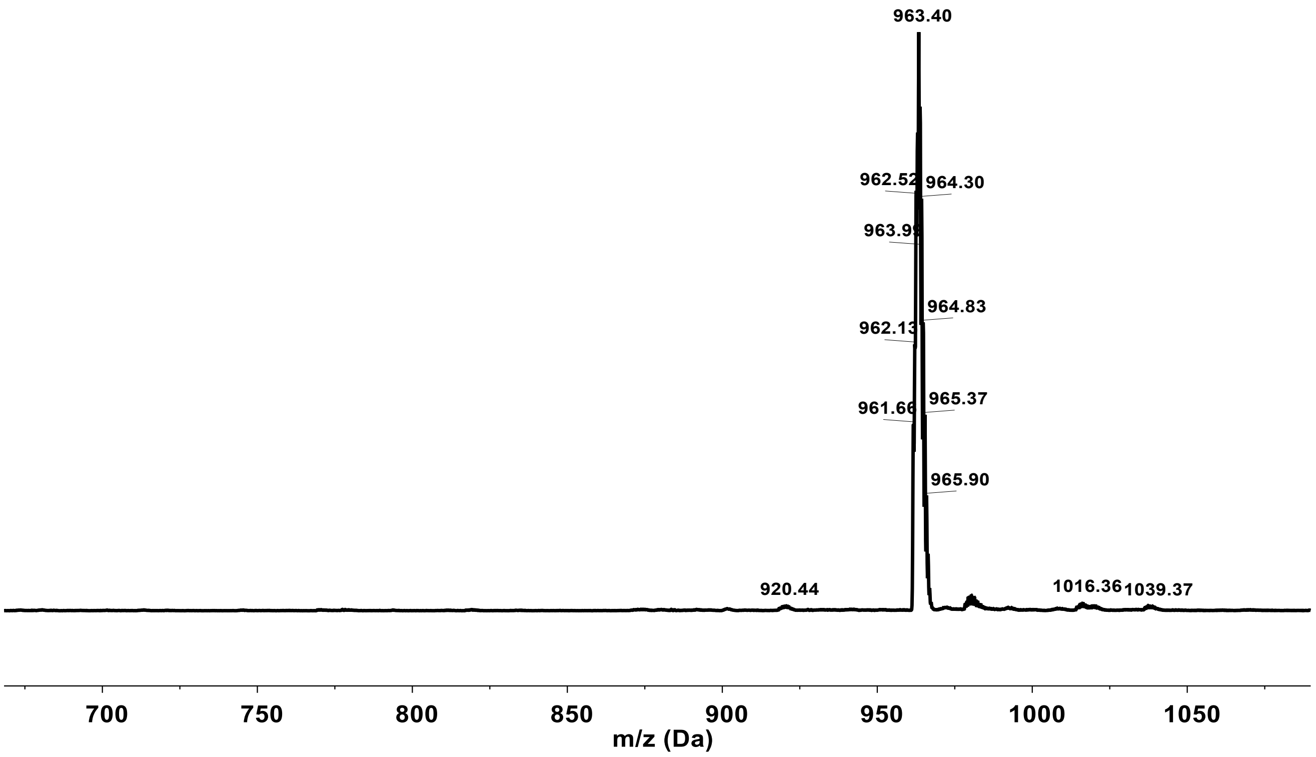

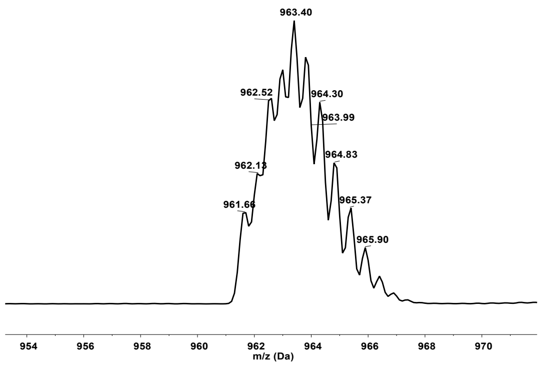

Figure S33 Experimental, and calculated ESI-MS pattern (in CH_3CN/0.1% HCOOH) of cage **L-Dibenz@*SS*-11.**

8 Computational Studies

The geometry optimization of cages **C_6@2**, **C_{10}@2**, **C_6@8**, and **C_{10}@8** were run with Gaussian 16 package. Molecular cages containing the guests were optimized using DFT-B3LYP calculations at the 6-31G(d) level of theory (DMSO as solvent).

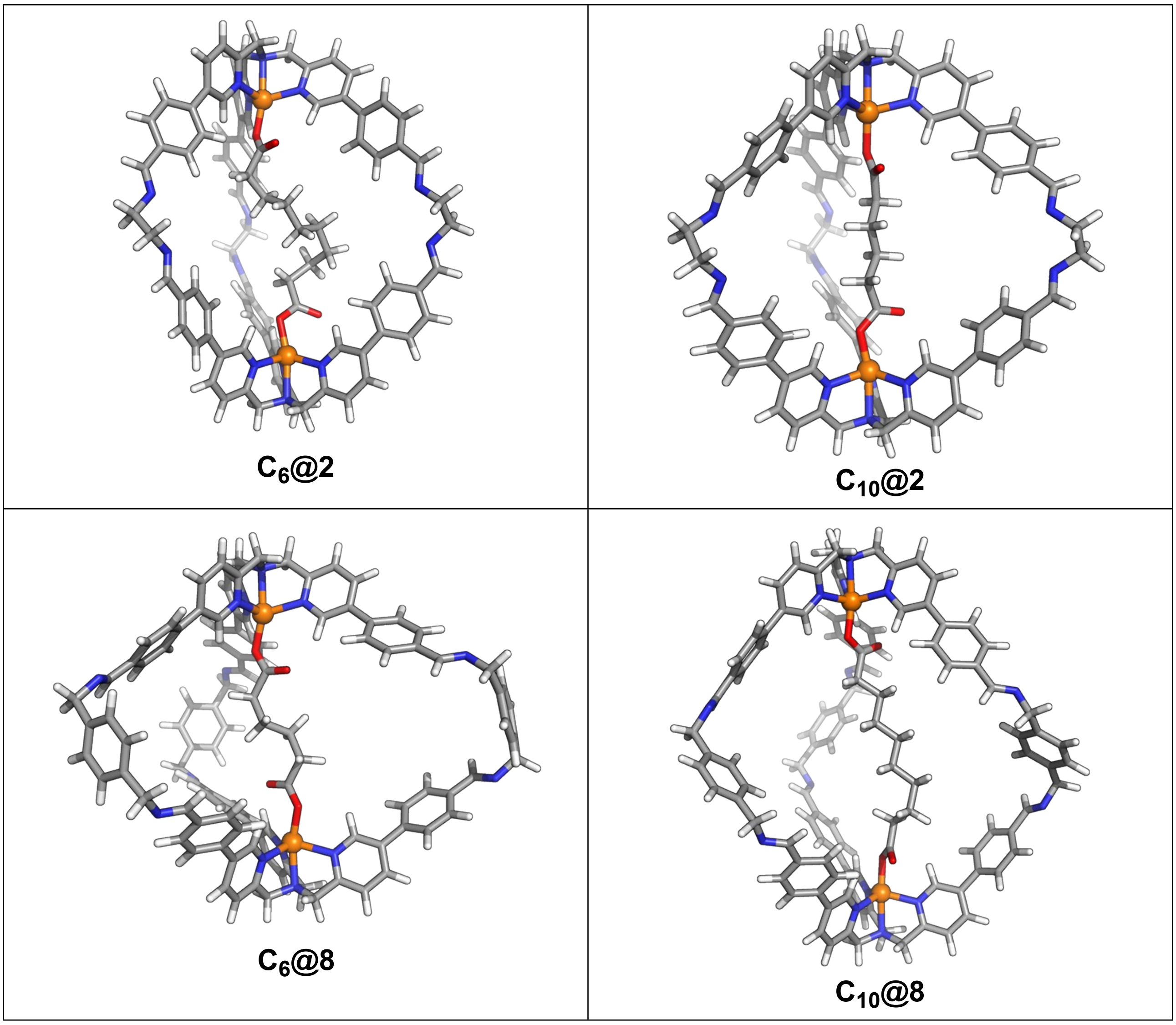

Figure S34 DFT-B3LYP minimized structures for the inclusion cages **C_6@2**, **C_{10}@2**, **C_6@8**, and **C_{10}@8**.

Table S1 Zn-Zn distance for the optimized structures.

Structure	Zn-Zn distance (Å)
**C_6@2**	12.22
**C_{10}@2**	13.66
**C_6@8**	11.70
**C_{10}@8**	16.22

9 Coordinates of the optimized structures

9.1 C_6@2

Symbol	X	Y	Z
C	-0.5755270	-96.686.840	25.982.860
C	-0.8344380	75.769.160	65.664.090
C	-0.4041450	24.681.560	-95.864.110
N	-13.637.520	-84.821.340	23.050.490
N	-16.227.840	66.792.080	57.390.990
N	-11.826.750	20.941.420	-84.154.190
C	-24.641.560	-83.326.050	29.321.220
C	-22.533.090	14.269.120	-86.042.760
C	-27.236.860	71.152.350	52.647.690
C	-33.491.550	-71.753.940	27.160.290
C	-31.373.240	-62.673.730	16.647.410
C	-44.463.390	-69.766.350	35.652.930
C	-40.119.290	-52.092.130	14.561.670
C	-53.116.050	-59.053.800	33.695.200
C	-51.219.500	-50.117.520	23.013.820
C	-36.063.900	62.820.270	44.303.060
C	-33.783.150	49.049.150	42.700.420
C	-47.137.360	68.589.530	37.928.360
C	-42.428.850	41.287.170	35.112.790
C	-55.703.330	60.845.610	30.156.580
C	-53.588.840	47.027.520	28.708.670
C	-31.398.540	10.159.360	-75.013.040
C	-41.999.750	0.1350010	-77.576.630
C	-29.761.620	15.103.470	-61.961.330
C	-50.792.270	-0.2343470	-67.447.220
C	-38.653.800	11.581.320	-51.891.120
C	-49.413.510	0.2858360	-54.462.930
C	-63.023.790	38.690.780	20.906.790
C	-76.638.240	41.956.840	19.813.990
C	-58.843.050	26.968.720	14.443.100
C	-85.217.060	33.791.930	12.546.260
C	-80.157.120	22.310.350	0.6510860
C	-59.390.660	-0.0357220	-43.991.890
C	-56.115.600	-0.0543240	-30.356.230
C	-72.779.690	-0.3047110	-47.269.880
C	-82.020.210	-0.5799050	-37.275.660
C	-77.905.750	-0.5611870	-23.982.740
C	-60.930.050	-39.205.180	20.555.490
C	-57.271.580	-27.316.210	14.080.720
C	-74.408.690	-40.404.950	24.321.150
C	-83.373.450	-30.152.190	21.619.380
C	-78.859.590	-18.737.660	15.064.150
N	-65.119.840	-0.2888110	-20.669.590
N	-67.156.970	18.988.610	0.7570810
N	-65.960.760	-17.449.540	11.363.200

C	-88.073.940	-0.7190280	11.843.000
C	-88.780.860	13.108.140	-0.1827040
C	-87.372.340	-0.8799710	-12.625.370
N	-84.065.090	-0.0775010	-0.0759810
Zn	-61.087.080	-0.0248980	-0.0108450
C	0.6074370	-93.691.070	35.428.250
C	0.8311580	15.666.440	-97.927.830
C	0.3973850	81.361.770	58.234.960
N	13.925.040	-82.276.360	31.022.010
N	16.215.650	14.197.390	-85.818.160
N	11.749.600	70.974.180	51.634.650
C	25.146.900	-84.606.360	25.426.590
C	22.211.920	66.740.900	57.578.190
C	27.121.890	20.758.380	-84.977.760
C	34.024.930	-73.864.540	20.664.670
C	45.415.420	-77.124.210	13.177.270
C	31.521.690	-60.346.640	23.576.800
C	54.108.680	-67.197.130	0.8777810
C	40.289.720	-50.444.710	19.350.290
C	51.823.750	-53.690.730	11.928.260
C	35.956.830	19.878.120	-73.221.990
C	46.788.350	28.686.950	-71.945.340
C	33.913.290	10.218.870	-63.225.700
C	55.329.860	27.918.200	-60.982.440
C	42.546.680	0.9314490	-52.397.770
C	53.446.110	18.143.190	-51.062.580
C	31.034.720	56.458.780	51.785.190
C	41.254.920	50.911.330	59.615.920
C	29.727.850	52.242.310	38.444.050
C	50.002.000	41.508.340	54.270.940
C	38.589.010	43.012.600	33.037.720
C	48.974.160	37.541.990	40.825.290
C	62.842.540	16.980.360	-39.671.570
C	76.346.640	20.676.220	-40.771.180
C	58.731.170	11.875.160	-27.275.470
C	84.889.080	19.259.290	-29.902.060
C	79.919.900	13.994.400	-18.009.650
C	58.961.790	28.301.880	34.963.240
C	55.912.040	19.727.830	24.295.510
C	72.200.390	28.006.110	39.653.710
C	81.513.830	19.527.090	33.817.910
C	77.615.450	11.454.680	23.172.050
C	61.529.730	-43.281.620	0.7803820
C	57.718.060	-29.910.290	0.5975360
C	75.106.410	-46.276.040	0.5819660
C	84.006.880	-36.279.970	0.2129300
C	79.337.110	-23.270.100	0.0524790
N	64.966.760	11.672.550	18.506.240
N	67.023.830	10.304.710	-16.844.520

N	66.354.420	-20.211.920	0.2515990
C	88.483.200	-12.006.860	-0.3718330
C	88.494.770	12.385.090	-0.5658920
C	87.236.690	0.1909830	16.450.900
N	84.100.000	0.0726560	0.2145890
Zn	61.139.060	0.0241150	0.1120760
H	-11.749.760	-104.702.240	30.597.990
H	-14.189.670	84.351.220	69.366.700
H	-0.9973350	24.349.830	-105.146.780
H	-28.191.980	-90.676.360	36.722.700
H	-25.847.660	11.283.500	-96.118.140
H	-30.801.170	81.393.800	54.600.040
H	-22.920.950	-64.202.950	10.018.440
H	-46.192.570	-76.630.150	43.903.990
H	-38.489.500	-45.501.380	0.6086680
H	-61.349.090	-57.611.340	40.616.120
H	-25.274.430	44.529.200	47.688.040
H	-49.009.280	79.245.600	38.992.100
H	-40.688.160	30.593.950	34.421.820
H	-64.015.590	65.644.990	25.085.710
H	-43.353.500	-0.2666350	-87.587.290
H	-21.613.890	21.975.570	-59.934.990
H	-58.750.100	-0.9379670	-69.679.060
H	-37.466.560	15.967.740	-42.029.800
H	-80.596.610	50.700.910	24.871.490
H	-48.512.940	23.691.950	14.608.370
H	-95.768.650	36.174.510	11.708.510
H	-45.935.780	0.0999770	-26.956.210
H	-76.042.540	-0.2723930	-57.610.320
H	-92.389.540	-0.7858810	-39.708.790
H	-47.089.440	-25.569.330	10.787.860
H	-77.986.280	-49.488.930	29.045.690
H	-93.835.040	-31.060.790	24.339.540
H	-98.519.140	-10.574.800	11.632.850
H	-87.297.740	0.0303820	19.808.590
H	-87.984.380	16.126.870	-12.336.220
H	-99.350.560	14.093.790	0.0978150
H	-97.794.440	-0.7419540	-15.784.770
H	-86.220.870	-19.376.120	-0.9990460
H	12.089.420	-102.890.500	36.270.450
H	14.132.430	19.859.350	-106.298.390
H	0.9934020	87.064.550	65.541.260
H	28.887.090	-94.857.710	23.899.400
H	25.338.390	70.654.200	67.393.070
H	30.580.640	27.416.020	-93.046.830
H	47.448.050	-87.517.760	10.721.840
H	22.731.290	-57.780.540	29.395.800
H	62.679.440	-70.021.350	0.2750490
H	38.292.970	-40.126.090	22.077.770

H	48.482.620	36.271.180	-79.547.720
H	25.600.900	0.3317910	-64.222.040
H	63.437.650	35.081.930	-60.102.250
H	40.997.970	0.1459080	-45.066.350
H	42.343.760	53.966.460	69.991.300
H	21.870.220	56.549.920	32.325.760
H	57.648.980	37.195.310	60.653.090
H	37.685.360	40.336.830	22.552.550
H	80.260.950	24.391.200	-50.183.420
H	48.463.760	0.8959700	-25.383.530
H	95.354.450	22.015.330	-30.669.830
H	45.885.360	19.160.540	20.195.470
H	75.305.760	34.636.860	47.655.670
H	91.783.870	19.314.570	37.304.220
H	47.481.280	-26.638.710	0.7432380
H	78.804.210	-56.338.760	0.7460520
H	94.533.050	-38.473.260	0.0690110
H	98.903.240	-14.378.770	-0.1184850
H	88.017.460	-11.004.700	-14.626.550
H	87.300.980	21.285.850	0.0625760
H	99.123.010	11.785.810	-0.8353290
H	97.607.030	0.5077780	18.175.800
H	86.136.330	-0.8004290	20.998.590
H	0.4847350	0.5686090	-100.889.720
H	-0.0599170	34.998.200	-94.427.590
H	-0.4834750	70.073.150	74.361.300
H	0.0501660	88.347.880	50.525.420
H	-0.1751110	-100.487.950	16.503.990
H	0.2054140	-91.360.830	45.367.540
C	18.795.860	-0.3122900	-0.4186110
H	18.926.900	0.5148280	-11.365.780
H	18.063.490	-12.335.050	-10.170.390
C	0.6617180	-0.2093520	0.5098500
H	0.7414030	-0.9765430	12.896.250
H	0.6863000	0.7596660	10.283.110
C	-0.6741370	-0.3620030	-0.2370540
H	-0.7600470	-13.788.930	-0.6389330
H	-0.6919510	0.3134670	-11.039.090
C	-18.925.740	-0.0708200	0.6488730
H	-18.942.560	0.9750700	0.9764190
H	-18.333.840	-0.6739510	15.676.600
C	32.381.270	-0.3724700	0.2917970
C	-32.513.700	-0.3898540	0.0139630
O	-42.313.780	0.3564520	0.4298010
O	-33.767.930	-13.271.690	-0.7894850
O	42.158.290	0.1787540	-0.3645880
O	33.607.700	-0.9476600	13.840.120

9.2 C_{10}@2

Symbol	X	Y	Z
C	0.2525500	-61.945.740	-67.670.870
C	0.1876780	95.483.200	-0.9754260
C	13.464.530	-42.458.630	82.008.510
N	11.274.460	-52.720.980	-60.591.770
N	10.725.210	84.013.350	-0.8258160
N	22.477.370	-38.065.610	71.483.800
C	23.807.840	-53.561.540	-62.810.210
C	35.040.320	-38.632.340	73.614.000
C	22.738.050	86.428.540	-0.4674370
C	33.721.630	-44.710.200	-56.438.550
C	29.961.460	-34.002.300	-48.147.010
C	47.372.180	-46.922.930	-58.760.620
C	39.572.200	-25.747.700	-42.446.610
C	57.011.640	-38.723.160	-52.974.390
C	53.297.870	-27.945.020	-44.759.230
C	33.080.130	76.017.180	-0.3335240
C	31.258.060	62.974.380	-0.8232130
C	45.354.140	79.278.960	0.2616840
C	41.482.710	53.603.850	-0.7390110
C	55.509.420	69.829.910	0.3678590
C	53.846.120	56.846.450	-0.1447770
C	44.865.050	-34.540.610	63.404.510
C	58.408.460	-33.095.290	66.753.380
C	40.879.020	-32.019.760	50.172.500
C	67.698.720	-28.883.140	57.248.900
C	50.135.630	-28.012.530	40.662.100
C	63.692.130	-26.171.390	44.071.630
C	64.922.360	47.007.080	-0.0771780
C	78.398.420	50.986.340	-0.0480150
C	62.517.130	33.197.590	-0.0501770
C	88.521.240	41.491.310	0.0285190
C	85.162.750	27.979.480	0.0562720
C	73.203.690	-21.343.710	33.805.870
C	68.755.380	-12.531.670	23.910.990
C	86.711.980	-25.139.660	33.148.060
C	94.810.680	-20.353.770	22.883.100
C	89.315.930	-12.040.310	13.116.980
C	63.642.980	-19.182.690	-38.767.120
C	61.798.300	-13.194.700	-26.231.460
C	75.861.470	-16.667.280	-45.220.910
C	85.540.240	-0.8824680	-39.065.910
C	82.877.830	-0.3262160	-26.580.460
N	76.442.120	-0.8227180	13.832.540
N	72.306.140	24.029.510	-0.0008000
N	71.077.790	-0.5387650	-20.420.280
C	92.897.720	0.5625620	-19.491.500
C	95.564.940	17.060.320	0.2019420

C	97.131.900	-0.7429880	0.0943300
N	91.348.440	0.4786970	-0.4889620
Zn	68.537.780	0.3282740	-0.1554640
C	-0.9101730	-54.532.990	-74.505.710
C	0.2263610	-51.337.110	76.320.850
C	-0.8868660	96.515.470	0.1255160
N	-18.151.440	-48.810.190	-64.682.100
N	-0.6985670	-43.647.510	68.107.680
N	-17.659.060	84.935.400	0.1857670
C	-30.689.370	-50.548.940	-66.247.910
C	-29.628.680	86.487.200	-0.2301420
C	-19.463.180	-45.342.370	70.153.360
C	-40.570.860	-45.295.850	-56.651.540
C	-54.274.700	-45.882.940	-59.567.840
C	-36.523.510	-39.691.300	-44.422.230
C	-63.694.890	-40.830.340	-50.626.660
C	-45.900.810	-34.816.030	-35.445.590
C	-59.678.060	-35.188.290	-38.407.660
C	-29.952.750	-38.165.570	62.686.350
C	-43.381.730	-41.608.180	64.804.180
C	-27.010.770	-27.889.430	53.559.930
C	-53.590.670	-35.039.520	58.007.210
C	-37.206.900	-21.238.130	46.856.300
C	-50.706.910	-24.681.900	48.958.040
C	-39.673.390	75.709.950	-0.1984040
C	-51.725.680	77.220.790	-0.8996420
C	-37.595.180	63.908.960	0.5346120
C	-61.311.470	67.121.440	-0.8969270
C	-47.257.760	53.946.430	0.5585730
C	-59.259.260	55.284.360	-0.1684020
C	-61.706.200	-17.628.720	41.952.600
C	-74.297.330	-15.840.800	47.925.370
C	-60.184.970	-12.577.790	28.966.320
C	-84.540.090	-0.9554460	40.953.640
C	-82.118.570	-0.4889680	28.055.530
C	-69.349.060	44.428.510	-0.1804510
C	-65.491.250	31.056.530	-0.0280930
C	-83.092.290	46.775.030	-0.3533620
C	-92.041.460	36.125.530	-0.3723400
C	-87.230.600	23.133.020	-0.2219150
C	-69.514.990	-29.718.650	-28.777.560
C	-65.981.130	-19.078.910	-20.405.500
C	-82.594.290	-34.664.610	-27.470.250
C	-91.227.620	-29.093.370	-18.102.690
C	-86.682.580	-18.823.830	-0.9831950
N	-74.090.450	20.789.960	-0.0501420
N	-70.050.040	-0.6404050	22.270.910
N	-74.211.750	-13.927.740	-11.129.180
C	-95.269.830	-13.258.780	0.1361390

C	-92.688.070	0.2425310	20.025.920
C	-96.230.570	10.952.810	-0.2735480
N	-90.837.360	0.0105280	0.5615220
Zn	-67.994.370	0.0783800	0.2406340
H	0.7923600	-67.865.760	-75.246.620
H	0.7425790	105.011.080	-0.9754670
H	18.698.990	-47.936.290	90.016.170
H	27.990.620	-61.055.620	-69.721.140
H	39.225.720	-42.129.500	83.184.470
H	26.169.580	96.676.470	-0.2519300
H	19.410.430	-32.183.440	-46.376.360
H	50.470.300	-55.205.620	-65.083.690
H	36.393.670	-17.350.280	-36.337.020
H	67.509.960	-40.876.840	-54.703.800
H	21.837.200	60.398.040	-12.962.400
H	46.935.080	89.302.520	0.6516320
H	39.951.070	43.763.180	-11.710.820
H	64.758.390	72.592.140	0.8639870
H	61.668.660	-35.074.260	76.934.050
H	30.454.640	-33.373.030	47.493.840
H	78.054.950	-27.445.990	60.188.950
H	46.904.790	-26.515.070	30.401.310
H	81.016.580	61.497.490	-0.1064280
H	52.467.670	29.132.330	-0.0505940
H	98.939.070	44.509.990	0.0531500
H	58.672.490	-0.8604920	24.072.010
H	90.848.410	-31.949.110	40.521.600
H	105.253.710	-23.238.360	22.287.210
H	52.887.450	-14.918.390	-20.262.270
H	77.805.750	-20.823.660	-55.054.070
H	95.078.650	-0.6976360	-43.895.140
H	103.100.380	0.3178630	-22.734.580
H	91.063.770	16.022.170	-22.450.030
H	96.617.310	14.701.680	12.674.870
H	105.365.730	20.600.350	-0.1445510
H	107.746.370	-0.6170700	0.3455980
H	96.621.220	-15.346.980	-0.6621610
H	-14.199.120	-61.451.730	-81.413.230
H	-0.2827290	-56.467.640	84.644.670
H	-14.436.290	105.862.890	-0.0537010
H	-34.819.730	-56.048.360	-74.854.070
H	-33.159.000	96.096.470	-0.6377190
H	-23.178.770	-52.406.390	77.749.120
H	-57.586.850	-50.187.930	-68.985.580
H	-25.940.860	-39.371.800	-42.055.840
H	-74.214.850	-41.088.720	-53.304.810
H	-42.524.720	-30.889.790	-25.904.790
H	-45.844.050	-49.582.390	71.770.800
H	-16.649.880	-25.098.150	51.940.830

H	-63.866.320	-38.146.260	59.622.100
H	-34.684.140	-13.092.390	40.130.590
H	-53.528.230	86.304.500	-14.689.600
H	-28.392.300	62.767.640	10.978.760
H	-70.342.030	68.393.610	-14.858.680
H	-45.560.650	45.142.410	11.704.850
H	-76.059.750	-19.297.710	58.057.330
H	-50.828.570	-13.581.130	23.570.430
H	-94.313.570	-0.8212790	45.469.090
H	-55.116.060	28.336.130	0.1154930
H	-86.843.320	56.913.770	-0.4474870
H	-102.683.340	37.866.170	-0.4917770
H	-56.417.550	-14.070.210	-21.271.630
H	-85.985.460	-42.924.510	-33.641.770
H	-101.365.200	-32.809.470	-17.024.210
H	-105.853.700	-13.339.890	-0.1560910
H	-94.381.610	-19.998.560	0.9958650
H	-91.663.010	13.183.530	21.865.460
H	-102.730.230	-0.0428020	23.433.230
H	-106.498.150	13.646.850	0.0085000
H	-96.606.330	0.7313540	-13.072.700
H	0.6876690	-59.050.060	70.005.890
H	0.8851740	-33.580.200	86.544.800
H	-0.3197350	94.573.870	-19.442.670
H	-0.3811500	97.435.510	10.950.500
H	-0.1703810	-68.968.680	-60.361.220
H	-0.4900340	-46.349.970	-80.511.200
C	16.120.210	-0.0209980	0.4250370
H	0.8167810	0.3655900	10.774.640
H	17.901.350	0.7566620	-0.3290720
C	11.106.350	-13.124.540	-0.2397200
H	10.409.280	-20.943.830	0.5296930
H	18.473.820	-16.701.730	-0.9700400
C	-0.2663100	-11.464.330	-0.9051390
H	-0.6769390	-21.362.410	-11.490.340
H	-0.9511210	-0.7124940	-0.1662600
C	-0.2485440	-0.2957400	-21.872.860
H	0.2596180	-0.8797450	-29.680.340
H	0.3714160	0.5986330	-20.365.260
C	-16.250.930	0.1594860	-27.166.380
H	-23.590.440	-0.6532780	-26.226.800
H	-15.242.280	0.3448420	-37.941.220
C	-21.855.570	14.507.740	-20.814.100
H	-28.964.360	19.047.020	-27.805.810
H	-13.615.800	21.681.640	-19.666.750
C	28.791.060	-0.1778210	12.904.320
C	-29.070.270	13.110.340	-0.7341070
H	-30.263.780	23.079.910	-0.2847510
H	-23.329.520	0.7369640	-0.0012560

C	-43.135.290	0.7179530	-0.8222310
O	-49.012.390	0.6221940	-19.122.300
O	-48.481.850	0.3941470	0.3179510
H	30.340.000	0.7366680	18.707.210
H	27.263.440	-10.030.920	19.975.420
C	41.471.920	-0.4717330	0.4891500
O	49.724.280	0.5268270	0.3809290
O	43.377.500	-16.008.840	0.0064250

9.3 C_6@8

Symbol	X	Y	Z
Zn	47.687.340	32.426.250	10.397.330
N	51.889.930	41.602.560	-0.8671470
N	39.107.570	46.363.050	24.202.140
C	59.156.150	52.948.470	-0.8256010
C	48.485.820	36.399.850	-20.586.910
N	63.811.710	20.041.460	18.258.790
C	48.071.900	53.528.050	31.313.150
C	25.969.660	48.971.060	25.600.790
C	62.913.160	58.141.290	0.5429580
C	63.020.140	59.559.030	-19.874.630
C	52.144.040	42.229.350	-32.797.590
H	42.646.580	27.259.220	-20.203.260
C	76.068.280	25.626.040	18.169.550
C	62.263.520	0.7723510	23.411.090
C	62.692.460	50.724.050	28.781.210
C	44.073.320	63.283.630	40.377.690
C	21.104.680	58.976.540	34.146.440
H	19.398.770	42.732.970	19.593.260
N	64.639.210	46.909.980	14.734.870
H	54.840.470	64.512.850	0.9207120
H	71.907.910	64.410.540	0.4821750
C	59.493.160	54.179.670	-32.163.500
H	68.936.170	68.631.230	-19.253.280
C	48.889.730	36.209.300	-45.933.570
C	77.151.720	39.577.500	12.488.900
C	87.229.650	18.903.370	23.055.990
C	72.866.180	0.0323240	28.831.120
H	52.174.970	0.3743380	23.030.310
H	65.973.480	42.365.840	35.058.390
H	68.775.070	59.426.400	31.585.840
C	30.549.850	66.015.950	41.782.240
H	51.511.380	68.903.060	45.925.310
C	0.6802780	62.776.920	34.921.610
H	62.786.940	59.011.950	-41.300.690
C	49.548.870	22.359.250	-48.117.210
C	45.643.430	44.581.430	-56.790.450
H	78.823.310	38.957.170	0.1674140

H	85.816.150	44.795.690	16.772.040
C	85.609.340	0.6207340	28.394.270
H	96.961.310	23.695.790	22.894.650
C	71.085.460	-12.974.110	35.108.020
H	27.332.630	73.966.780	48.424.390
C	-0.1298400	63.395.820	23.474.730
C	0.1350460	66.926.520	47.235.720
C	47.299.850	17.086.290	-60.833.330
H	52.195.410	15.698.690	-39.954.680
C	43.393.230	39.328.440	-69.411.880
H	44.736.360	55.292.010	-55.247.250
H	94.141.390	0.0982000	32.594.300
C	60.196.550	-15.795.480	43.502.390
C	80.919.940	-22.893.680	33.275.940
C	-14.270.730	68.468.330	24.247.250
H	0.2693870	60.354.220	13.843.010
C	-11.528.800	71.976.950	47.987.570
H	0.7295050	66.150.560	56.290.010
C	44.311.170	25.488.340	-71.655.830
H	48.001.620	0.6350120	-62.392.960
H	40.859.080	45.820.370	-77.722.860
C	59.334.080	-28.062.180	50.082.730
H	52.577.690	-0.8247260	45.217.580
C	80.017.610	-35.101.900	39.769.100
H	89.270.330	-21.014.400	26.591.800
C	-19.470.210	73.037.610	36.443.350
H	-20.319.730	69.123.550	15.237.480
H	-15.622.560	75.264.300	57.479.210
C	41.923.760	19.867.440	-85.075.680
C	69.258.530	-37.824.880	48.386.490
H	50.930.650	-30.025.960	56.694.740
H	87.596.540	-42.719.660	38.293.770
C	-32.988.080	78.885.630	37.124.000
N	39.083.820	27.318.040	-95.032.000
H	42.566.070	0.8908500	-85.919.290
C	68.242.610	-50.788.910	55.326.330
N	-37.707.130	83.559.940	48.011.300
H	-38.729.700	78.936.900	27.728.560
C	35.636.390	20.973.190	-107.739.350
N	76.774.920	-60.091.290	53.461.460
H	59.556.870	-52.029.590	61.978.540
C	-51.464.310	88.437.990	48.158.920
C	20.580.480	21.918.430	-109.665.510
H	40.909.980	26.369.670	-115.714.670
H	38.772.770	10.428.270	-108.189.740
C	74.270.040	-73.094.210	59.708.360
C	-60.188.960	78.661.310	55.900.170
H	-51.460.660	98.273.720	53.055.630
H	-55.569.180	89.800.180	38.029.410

C	12.918.810	10.455.920	-112.053.160
C	13.961.670	34.169.790	-108.127.920
C	67.973.610	-82.245.510	49.331.400
H	83.899.330	-77.088.370	63.132.290
H	67.637.690	-72.341.770	68.459.770
Zn	-47.541.650	-33.197.000	-0.7057780
C	-55.726.250	73.060.940	67.937.260
C	-72.552.260	74.523.930	50.801.240
C	-0.1008440	11.126.820	-112.453.350
H	17.862.870	0.0853470	-113.330.360
C	0.0029490	34.805.500	-108.316.610
H	19.738.470	43.175.680	-106.258.210
C	74.002.550	-84.036.600	36.811.350
C	55.495.600	-88.142.960	51.626.570
N	-40.706.310	-51.738.250	0.1261830
N	-49.184.360	-33.945.410	-28.485.930
N	-64.956.010	-24.609.500	0.3271960
C	-63.089.820	63.066.670	74.298.900
H	-46.188.400	76.182.700	72.074.100
C	-80.014.260	64.679.880	57.276.190
H	-76.239.800	78.806.530	41.508.450
C	-0.7646060	23.264.320	-110.256.630
H	-0.6792470	0.2063480	-113.972.320
H	-0.4927330	44.342.510	-106.655.950
C	67.396.310	-90.951.050	26.658.990
H	83.689.410	-79.535.620	34.831.150
C	48.980.350	-95.239.870	41.539.640
H	50.670.590	-86.925.230	61.296.640
C	-50.380.360	-60.817.570	0.3749530
C	-27.816.500	-55.165.670	0.3141270
C	-56.230.700	-44.291.840	-33.488.320
C	-44.443.280	-24.543.020	-36.843.040
C	-76.974.370	-29.287.350	-0.0609540
C	-64.365.110	-15.413.820	13.052.550
C	-75.201.130	58.569.530	68.931.850
H	-59.211.040	58.508.290	83.379.420
H	-89.397.870	61.327.380	52.948.860
C	-22.810.040	23.715.040	-109.130.380
C	54.696.510	-96.432.750	28.808.660
H	72.043.220	-91.827.290	16.864.530
H	39.146.270	-99.443.780	43.403.780
C	-64.583.770	-56.764.580	0.0649220
C	-47.419.410	-73.479.680	0.8675500
C	-23.922.230	-67.905.800	0.7537370
H	-20.632.520	-47.285.770	0.1047850
C	-61.551.500	-54.359.890	-23.556.310
C	-58.521.850	-45.624.030	-47.144.740
C	-46.488.850	-24.930.170	-50.707.610
H	-38.822.980	-16.526.140	-32.153.170

C	-76.991.140	-39.659.550	-11.583.810
C	-88.817.440	-24.798.000	0.5153970
C	-75.742.570	-10.458.400	19.584.580
H	-54.409.240	-11.862.280	15.507.780
C	-82.316.030	46.497.120	74.828.590
N	-27.211.620	12.964.630	-100.275.210
H	-27.448.280	22.058.030	-118.947.300
H	-25.957.470	33.660.460	-105.601.730
C	46.939.950	-102.802.020	17.372.530
N	-64.720.700	-47.666.950	-10.873.410
H	-68.850.220	-51.450.620	0.9227260
H	-70.800.170	-65.660.280	-0.1028920
C	-34.147.870	-77.029.250	10.568.580
H	-55.416.060	-80.554.410	10.594.580
C	-0.9777290	-72.222.290	0.8457310
H	-53.859.070	-61.915.740	-21.614.780
H	-70.232.590	-59.636.660	-27.724.980
C	-53.630.500	-35.918.230	-55.759.140
H	-64.295.190	-54.010.040	-50.890.190
C	-41.815.100	-14.303.390	-59.908.720
H	-77.221.600	-34.638.320	-21.324.080
H	-86.014.820	-45.891.400	-10.973.140
C	-88.181.750	-15.351.660	15.287.930
H	-98.336.600	-28.835.160	0.1867390
C	-75.092.720	-0.0620050	30.634.140
N	-83.909.960	36.533.240	64.219.090
H	-92.320.270	49.207.580	78.427.950
H	-76.576.180	42.646.820	83.394.520
C	-31.153.610	15.957.790	-88.519.070
N	33.187.610	-97.892.100	17.571.900
H	46.599.210	-113.716.000	18.567.690
H	52.042.310	-100.675.740	0.7847440
H	-31.682.980	-87.052.660	13.909.270
C	-0.0342430	-68.648.410	-0.1304080
C	-0.5814790	-81.029.100	18.716.900
H	-55.694.030	-36.621.110	-66.386.750
C	-42.521.620	-0.0700320	-56.519.570
C	-37.198.070	-17.798.270	-72.753.130
H	-97.298.880	-11.997.470	20.123.510
C	-65.313.110	-0.1341700	40.675.700
C	-84.926.540	0.9426640	31.531.320
C	-75.432.900	27.019.140	63.543.700
C	-34.711.380	0.5566030	-78.681.840
H	-31.929.810	26.378.590	-85.049.590
C	29.650.750	-89.334.850	0.8799150
C	12.494.130	-74.099.280	-0.1073840
H	-0.3177970	-61.974.390	-0.9389590
C	0.6942490	-86.433.210	18.938.430
H	-12.831.400	-83.630.810	26.586.280

C	-39.019.560	0.9097330	-65.811.440
H	-46.208.640	0.2274870	-46.745.720
C	-33.713.000	-0.8057730	-81.968.050
H	-36.236.530	-28.269.550	-75.462.820
C	-65.513.230	0.7578750	51.397.780
H	-57.736.220	-0.9116230	40.315.540
C	-85.084.800	18.314.280	42.158.120
H	-92.406.940	10.341.190	23.711.500
C	-75.420.100	17.464.170	52.321.650
H	-67.509.820	25.645.330	71.065.780
C	16.234.450	-83.222.890	0.8897100
H	36.415.700	-86.019.240	0.0768630
H	19.594.200	-71.436.420	-0.8864710
H	0.9893060	-93.286.810	26.811.180
H	-39.802.310	19.594.810	-63.093.760
H	-30.175.610	-10.817.620	-91.842.330
H	-57.951.260	0.6802150	59.170.020
H	-92.637.800	26.075.420	42.751.720
C	19.341.210	0.2841580	-0.2284970
C	0.4394850	0.1394380	-0.5618650
C	-0.4767740	-0.0649480	0.6544080
C	-19.672.170	-0.2338630	0.3119400
C	24.054.750	16.371.240	0.3310580
O	16.140.570	25.626.710	0.5546590
O	36.948.360	16.923.120	0.5155130
C	-24.254.270	-16.289.630	-0.1460080
O	-16.358.010	-25.791.760	-0.2216090
O	-37.019.580	-16.927.860	-0.4051110
H	25.346.400	0.0992720	-11.290.190
H	22.450.820	-0.4911630	0.4857990
H	0.3212940	-0.7192700	-12.349.020
H	0.1104010	10.257.450	-11.200.230
H	-0.1422900	-0.9452470	12.183.210
H	-0.3671480	0.8005830	13.185.840
H	-22.755.640	0.4879320	-0.4568060
H	-25.793.510	0.0084470	11.913.950

9.4 C_{10}@8

Symbol	X	Y	Z
Zn	80.870.870	0.3443480	0.2707830
N	83.749.210	0.6033940	23.337.350
N	88.353.640	-14.736.460	-0.4425640
C	96.262.410	0.4786910	28.121.500
C	73.626.550	0.8804020	31.686.900
N	84.432.500	20.820.090	-0.8138390
C	101.274.010	-15.184.920	-0.8125800
C	80.511.660	-25.449.360	-0.6223030
C	107.048.180	0.1065170	18.103.450

C	98.897.520	0.6303410	41.727.700
C	75.340.290	10.538.740	45.466.570
H	63.819.380	0.9355580	27.086.040
C	96.468.780	26.701.680	-0.6767250
C	75.091.260	26.466.840	-15.957.210
C	109.369.780	-0.2488760	-0.6132520
C	106.617.350	-26.656.100	-13.996.800
C	84.950.670	-37.314.270	-12.126.700
H	70.234.760	-24.339.740	-0.2974190
N	103.730.380	0.5878540	0.4587080
H	107.714.840	-0.9868580	17.690.370
H	116.842.090	0.4694720	21.494.200
C	88.402.030	0.9125890	50.420.400
H	109.049.290	0.5346120	45.432.900
C	63.759.280	13.693.770	54.157.480
C	106.350.880	20.243.120	0.2779700
C	99.395.230	38.603.430	-13.418.770
C	77.107.840	38.462.690	-22.857.530
H	65.769.130	20.997.790	-16.693.040
H	109.009.170	0.3316950	-15.424.570
H	119.924.010	-0.4943340	-0.4345710
C	98.398.350	-37.688.700	-16.147.060
H	117.081.520	-26.910.660	-16.857.120
C	75.444.590	-48.514.370	-14.113.090
H	90.380.150	10.486.740	61.008.540
C	53.942.820	22.797.460	49.919.030
C	62.294.840	0.7602290	66.767.790
H	105.269.290	25.098.020	12.547.880
H	116.641.700	22.165.650	-0.0534210
C	89.674.340	44.550.310	-21.398.430
H	109.199.760	43.126.890	-12.349.950
C	66.253.460	44.194.230	-31.160.760
H	102.471.450	-46.658.110	-20.712.990
C	66.364.580	-51.992.570	-0.3981740
C	74.916.460	-55.498.890	-26.323.620
C	43.000.010	25.695.530	58.021.040
H	54.997.750	27.829.660	40.353.890
C	51.293.910	10.394.830	74.781.830
H	69.708.040	0.0427900	70.171.740
H	91.935.110	53.760.990	-26.681.930
C	57.714.290	35.823.180	-38.536.010
C	64.096.090	58.101.920	-31.650.940
C	56.903.900	-61.985.580	-0.6076160
H	66.796.050	-46.951.700	0.5628530
C	65.341.570	-65.344.690	-28.465.250
H	81.814.770	-52.904.450	-34.305.580
C	41.463.290	19.480.780	70.500.630
H	35.550.980	32.851.230	54.632.310
H	50.142.410	0.5553180	84.424.520

C	47.328.100	41.191.400	-46.089.080
H	59.274.230	25.076.870	-38.478.490
C	53.646.100	63.434.250	-39.092.630
H	70.482.650	64.747.960	-25.902.810
C	56.107.550	-68.642.950	-18.396.030
H	49.959.450	-64.562.050	0.1880610
H	64.800.580	-70.496.410	-38.000.680
C	29.607.320	22.630.950	78.659.790
C	45.066.810	55.032.270	-46.393.290
H	40.856.920	34.569.300	-51.785.910
H	51.932.780	74.147.820	-39.282.390
C	45.453.000	-78.620.790	-20.435.960
N	26.635.600	16.393.620	89.400.250
H	23.316.970	30.809.270	74.802.880
C	33.731.820	60.277.680	-54.205.330
N	42.693.760	-83.682.080	-31.827.800
H	39.779.590	-81.305.020	-11.384.610
C	14.565.680	20.615.130	96.429.450
N	30.078.960	72.511.340	-53.891.430
H	28.451.150	52.830.700	-60.368.680
C	31.546.990	-93.163.270	-32.395.320
C	0.4054460	0.9634370	97.027.640
H	17.417.690	23.326.690	106.669.890
H	10.155.010	29.633.850	91.823.770
C	18.635.730	76.269.650	-62.121.630
C	20.084.450	-87.602.230	-40.693.810
H	35.236.500	-102.371.820	-37.065.680
H	27.923.630	-95.751.110	-22.310.420
C	-0.3240390	0.7390740	108.751.820
C	0.1053800	0.1878340	85.740.040
C	0.6992130	81.506.420	-53.846.600
H	21.899.590	84.172.030	-68.998.630
H	15.181.240	67.825.040	-68.348.630
Zn	-81.138.970	-0.2168720	-0.1837550
C	17.658.640	-92.086.530	-53.720.720
C	11.676.140	-77.713.660	-35.409.380
C	-13.372.770	-0.2223970	109.164.900
H	-0.1035270	13.232.550	117.657.460
C	-0.9005800	-0.7748140	86.181.800
H	0.6663050	0.3349500	76.553.270
C	0.3236570	75.277.260	-41.861.350
C	-0.0568040	92.398.060	-58.314.890
N	-86.952.820	16.035.850	-10.692.170
N	-87.111.880	-0.5155790	17.857.760
N	-83.449.670	-18.815.420	-14.017.430
C	0.7117930	-86.854.060	-61.240.480
H	23.995.430	-99.811.670	-58.015.560
C	0.1192330	-72.430.180	-42.930.170
H	13.333.120	-74.103.880	-25.282.540

C	-16.390.140	-0.9933870	97.896.920
H	-18.919.430	-0.3777300	118.389.430
H	-11.151.140	-13.640.700	77.294.580
C	-0.7791520	79.788.330	-34.637.430
H	0.9022540	66.876.870	-38.124.930
C	-11.664.320	96.870.340	-51.096.930
H	0.2198900	97.441.720	-67.545.720
C	-99.372.090	16.965.960	-15.766.810
C	-78.548.610	26.447.540	-11.652.920
C	-100.112.530	-0.3249630	20.798.700
C	-78.447.910	-0.8429930	27.578.310
C	-95.627.760	-24.509.770	-14.634.430
C	-73.236.830	-24.056.220	-20.945.510
C	-0.1214040	-76.906.740	-55.989.660
H	0.5382420	-90.552.410	-71.321.510
H	-0.5221920	-64.811.030	-38.595.410
C	-27.361.120	-20.460.760	98.161.220
C	-15.424.150	90.645.430	-39.152.600
H	-10.502.290	74.802.510	-25.358.260
H	-17.389.260	105.352.890	-54.777.380
C	-108.043.180	0.4532940	-14.893.730
C	-103.683.550	28.639.320	-22.064.310
C	-81.992.300	38.522.530	-17.816.980
H	-68.645.350	24.767.070	-0.7578370
C	-109.261.030	0.0685400	0.9338950
C	-104.709.730	-0.4442630	33.907.540
C	-82.193.740	-0.9821630	40.982.520
H	-68.212.270	-10.006.120	24.339.530
C	-106.523.510	-18.352.840	-0.6026580
C	-97.775.580	-35.894.690	-22.396.440
C	-74.457.930	-35.445.300	-28.964.500
H	-63.707.250	-19.027.710	-19.792.700
C	-12.175.540	-70.787.900	-64.578.950
N	-38.795.640	-16.311.650	90.018.460
H	-30.865.210	-21.754.030	108.467.950
H	-23.298.560	-30.139.620	94.781.660
C	-27.444.180	95.407.230	-31.149.960
N	-104.085.290	-0.4038290	-0.3605660
H	-106.674.970	-0.1238530	-24.114.370
H	-118.664.930	0.7290150	-14.460.120
C	-94.943.050	39.416.060	-23.162.780
H	-113.763.550	29.260.330	-26.032.310
C	-72.097.840	49.526.460	-18.619.850
H	-109.769.410	11.629.170	0.8931160
H	-119.473.690	-0.2872920	11.251.730
C	-95.710.660	-0.7608960	44.045.120
H	-115.232.010	-0.2967000	36.108.260
C	-72.037.230	-13.450.060	51.149.170
H	-106.476.800	-23.448.280	0.3680150

H	-116.388.240	-20.171.820	-10.489.860
C	-87.158.270	-41.415.510	-29.504.030
H	-107.662.590	-40.338.540	-22.877.710
C	-62.719.540	-40.710.740	-36.323.540
N	-24.210.550	-67.572.530	-56.995.250
H	-14.971.690	-78.009.800	-72.356.640
H	-0.8179690	-61.899.990	-69.783.900
C	-41.136.750	-23.040.200	79.422.230
N	-38.423.770	85.761.880	-31.913.200
H	-31.021.110	104.888.320	-35.337.770
H	-24.447.230	97.303.020	-20.707.290
H	-98.238.900	48.580.940	-27.962.680
C	-63.866.930	52.507.320	-0.7640000
C	-70.500.950	56.988.320	-30.452.310
H	-99.236.770	-0.8663810	54.259.640
C	-62.232.130	-23.076.160	48.241.030
C	-71.793.750	-0.7186390	63.754.610
H	-88.784.320	-50.207.830	-35.662.180
C	-53.673.130	-31.929.020	-42.513.170
C	-60.269.380	-54.554.180	-37.120.930
C	-27.942.590	-55.360.100	-56.897.040
C	-52.023.170	-19.788.750	70.039.210
H	-34.925.010	-31.673.500	76.558.280
C	-41.829.550	79.926.930	-21.079.300
C	-54.254.840	62.538.580	-0.8509670
H	-65.114.540	47.078.530	0.1682790
C	-60.795.070	66.890.740	-31.358.630
H	-76.700.190	54.757.540	-39.090.450
C	-52.440.050	-26.262.080	57.603.680
H	-62.356.040	-28.202.510	38.669.140
C	-61.894.000	-10.239.370	73.019.340
H	-79.228.820	0.0361540	66.159.440
C	-42.507.840	-36.832.110	-49.226.470
H	-55.504.600	-21.227.230	-42.299.340
C	-49.059.100	-59.428.500	-43.730.210
H	-67.065.670	-61.508.390	-32.278.010
C	-39.984.290	-50.615.360	-49.856.340
H	-22.237.450	-47.544.470	-62.160.480
C	-52.472.200	69.757.450	-20.401.680
H	-36.814.050	82.089.320	-11.514.170
H	-47.999.100	64.747.410	0.0102610
H	-59.451.740	72.454.280	-40.578.550
H	-44.964.870	-33.783.700	55.211.900
H	-61.616.050	-0.5208730	82.630.750
H	-35.664.530	-29.895.430	-54.044.950
H	-47.151.010	-70.101.570	-44.184.290
C	-38.638.880	0.0299340	0.0291360
C	-28.511.240	-10.474.360	-0.3749830
C	-14.360.040	-0.4838020	-0.5331770

C	-0.3997130	-15.246.460	-0.9704470
C	10.034.860	-0.9297970	-11.333.940
C	20.632.380	-19.577.820	-15.482.230
C	34.638.100	-13.673.040	-17.709.530
C	-52.850.480	-0.5005080	0.1982560
O	-62.170.950	0.2814050	-0.2540450
O	-54.902.480	-15.965.750	0.7450130
C	40.895.350	-0.7727620	-0.5057060
C	55.137.590	-0.2582390	-0.6917740
O	59.792.660	-0.0474560	-18.235.310
O	61.685.510	-0.0643720	0.4144390
H	-38.729.590	0.8470030	-0.6996410
H	-35.637.630	0.4707470	0.9914830
H	-31.687.900	-15.083.340	-13.214.600
H	-28.606.520	-18.467.990	0.3756850
H	-14.531.040	0.3363990	-12.663.250
H	-11.172.490	-0.0328030	0.4185800
H	-0.7170700	-19.813.680	-19.196.410
H	-0.3677280	-23.408.630	-0.2334970
H	0.9704350	-0.1237770	-18.820.960
H	12.947.470	-0.4539590	-0.1868290
H	17.376.250	-24.502.320	-24.754.600
H	21.187.760	-27.495.980	-0.7861240
H	34.232.320	-0.5951620	-25.495.300
H	41.270.920	-21.519.630	-21.568.310
H	40.973.500	-15.089.010	0.3081550
H	34.936.910	0.0712140	-0.1322430

10 References

(1) Gaussian 16, Revision C.01, M. J. Frisch, G. W. Trucks, H. B. Schlegel, G. E. Scuseria, M. A. Robb, J. R. Cheeseman, G. Scalmani, V. Barone, G. A. Petersson, H. Nakatsuji, X. Li, M. Caricato, A. V. Marenich, J. Bloino, B. G. Janesko, R. Gomperts, B. Mennucci, H. P. Hratchian, J. V. Ortiz, A. F. Izmaylov, J. L. Sonnenberg, D. Williams-Young, F. Ding, F. Lipparini, F. Egidi, J. Goings, B. Peng, A.Petrone, T. Henderson, D. Ranasinghe, V. G. Zakrzewski, J. Gao, N. Rega, G. Zheng, W. Liang, M. Hada, M. Ehara, K. Toyota, R. Fukuda, J. Hasegawa, M. Ishida, T. Nakajima, Y. Honda, O. Kitao, H. Nakai, T. Vreven, K. Throssell, J. A. Montgomery Jr., J. E. Peralta, F. Ogliaro, M. J. Bearpark, J. J. Heyd, E. N. Brothers, K. N. Kudin, V. N. Staroverov, T. A. Keith, R. Kobayashi, J. Normand, K. Raghavachari, A. P. Rendell, J. C. Burant, S. S. Iyengar, J. Tomasi, M. Cossi, J. M. Millam, M. Klene, C. Adamo, R. Cammi, J. W. Ochterski, R. L. Martin, K. Morokuma, O. Farkas, J. B. Foresman, D. J. Fox, Gaussian, Inc., Wallingford CT, 2016.

(2) 2016. GaussView, Version 6, Dennington, R., Keith, T. A., Millam, J. M., Semichem Inc., Shawnee Mission, KS.

(3) Bravin, C.; Badetti, E.; Puttreddy, R.; Pan, F.; Rissanen, K.; Licini, G.; Zonta, C. *Chem. Eur. J.* **2018**, *24*, 2936–2943.

(4) Anjum, A.; Paswan, S.; Kumar, M.; Ali, H.; Dubey, R.K. *Asian J. Chem.* **2019**, *31*, 943-950.
